# Tension experience induced by tonal and melodic shift at music phrase boundaries

**DOI:** 10.1038/s41598-022-11949-4

**Published:** 2022-05-18

**Authors:** Ning Zhang, Lijun Sun, Qiong Wu, Yufang Yang

**Affiliations:** 1 CAS Key Laboratory of Behavioral Science, Institute of Psychology, Chinese Academy of Sciences, Beijing, China; 2 Department of Psychology, University of Chinese Academy of Sciences, Beijing, China; 3 College of Art, Nanjing University of Aeronautics and Astronautics, Nanjing, China

**Keywords:** Perception, Human behaviour

## Abstract

Music tension is a link between music structures and emotions. As music unfolds, developmental patterns induce various emotional experiences, but the relationship between developmental patterns and tension experience remains unclear. The present study compared two developmental patterns of two successive phrases (tonal shift and melodic shift) with repetition condition to investigate the relationship with tension experience. Professional musicians rated on-line felt tension and EEG responses were recorded while listening to music sequences. Behavioral results showed that tension ratings under tonal and melodic shift conditions were higher than those under repetition conditions. ERP results showed larger potentials at early P300 and late positive component (LPC) time windows under tonal shift condition, and early right anterior negativity (ERAN) and LPC under melodic shift condition. ERSP results showed early beta and late gamma power increased under tonal shift condition, theta power decreased and alpha power increased under melodic shift condition. Our findings suggest that developmental patterns play a vital role in tension experiences; tonal shift affects tension by tonal shift detection and integration, while melodic shift affects tension by attentional processing and working memory integration. From the perspective of Event Structure Processing Model, solid evidence was given to specify the time-span segmentation and reduction.

## Introduction

When music unfolds over time, listeners’ emotions are modulated continuously and dynamically^1^. How does music induce emotion? It is well-acknowledged that tension is a bridge between sound and emotion. More specifically, the alternation between tension and relaxation plays a fundamental role in emotion generation^2^. Tension experience is associated with dissonance, conflict, instability and yearning for resolution^3,4^, and relies on the cognition of music structures through the processes of build-up, violation and fulfillment of expectation^5–8^.

As depicted by Generative Theory of Tonal Music (GTTM)^2^ and the Tonal Tension Model (TTM)^9^, music is highly hierarchical in harmonic and tonal structure. In harmonic progression, the tonic chord is the most stable one, while the dominant and subdominant chord are relatively less stable. The leading chord is unstable, and unstable chords could induce higher tension experience than stable chords^10,11^. Tonal hierarchy also affects tension experience. It was found that moving away from the tonal center increases the tension experience, while returning to the tonal center induces a relaxation experience^9–12^. The tension-resolution patterns are organized into prolongation structures, with local tension-resolution patterns embedded in a global context. This model mainly focused on music events from the perspective of tonal structure, for instance, a certain tone or chord.

McAdams and Bigand^13^ proposed the Event Structure Processing Model to specify the cognitive processing stages of real-time music perception. In this model, five stages follow successively: reading the acoustic surface, auditory image formation, time-span segmentation, time-span reduction and prolongation reduction. During the first two stages, music is processed in an elementary fashion, and during the time-span segmentation stage, music is segmented into chunks based on proximity rules and metric structuring. During the time-span reduction stage, event hierarchies are established, and information of the music is coded into a more abstract form, where the stable or important notes or chords are extracted. The musical surface is divided into groups or segments, and the events in each group are compared for their “relative stability”. The structural importance of events depends on rhythmic and melodic features and the way they interact with the tonal hierarchy. This model distinguishes tonal hierarchy (as in GTTM) from event hierarchy, with the former being an atemporal mental schema representing a system of culturally determined pitch relationships and the latter being a structure that listeners must infer from the ongoing temporal sequence of musical segments. Event hierarchy is established by considering an event’s tonal weight and its rhythmic and metric value within a given group of notes. In the last processing stage of prolongation reduction, the relationships between events within groups, between groups within sections, and between sections within the entire work are analyzed and established.

Lerdahl^14^ proposed three types of prolongation connections of music event structure, i.e., (1) strong prolongation, as when an event repeats; (2) weak prolongation, as when an event repeats in an altered version; and (3) progression, as when an event connects to a completely different event. The output of prolongation reduction is the hierarchy of tension and relaxation. However, the relationship between various prolongation connections and tension experience is not clarified. It is worth investigating whether tension levels vary with connections. Moreover, for the weak prolongation connection pattern, there could be various subtypes, for instance, event repeats in the pattern of tonal modulation or in other melodic patterns. The relationship between these developmental patterns and tension-relaxation experiences and the underlying mechanism still need further investigation.

Previous studies on music tension are mainly concerned with the role of music syntactic structures with violation and integration paradigm in the framework of predictive coding. The studies using tension rating after the ending of music phrase found that syntactic violations induced higher tension experiences, and larger early right anterior negativity (ERAN) and N5 were induced by syntactically violated chords than congruent chords^11,12,15–17^. Steinbeis and Koelsch^15^ explained that the former might reflect general attentional processing and the latter semantic processing of tension-resolution patterns. Lehne et al.^17^ compared different versions of two piano pieces by Mendelssohn and Mozart where dynamics, agogics, harmonic-syntactic features were original or controlled. The results showed that tension was mainly induced by harmonic-syntactic structure^18^. Sun et al.^8^ investigated tension experience modulated by nested structure, and revealed that double-nested structure (where the key shifted twice and returned to the first key, for instance, C-G-D-G-C) induced higher tension than single-nested structure (as in C-G-C), and larger late positive component (LPC) was induced at the ending chord under nested structures condition than non-nested structure condition, which suggested that the effect of prediction violation on music tension is dependent on the hierarchical level of music structures.

Event structure is different from syntax structure in that it is organized in terms of event relations, and more closely related to human experience or the schemas in listeners’ minds. Studies using classical music pieces as materials found that the event structure of music affected tension experience on a larger time scale. Krumhansl^16^ asked participants to rate the tension experience while listening to Mozart’s piano sonata K282 and found that tension was associated with the development of music ideas and modulated by the boundaries of music ideas, with tension increasing before the boundary and decreasing after the boundaries. This might be because listeners predicted the ending of an idea and expected a certain group of notes or chords to resolve the tension induced by music progression, and when the expected notes were presented, tension experience decreased immediately, suggesting that tension was modulated by the positions of boundaries in event structure.

How is tension experience affected by developmental patterns, and what underlying cognitive processes are involved? The Event Segmentation Model^19^ proposed that when processing the large-scale structure of an event, for instance, a movie or a story, the observer attempts to predict the following information as an ongoing part of perception^20–22^. When the following information violates the prediction, existing event models are modified to fit the newly processed information. When prediction error increases, observers update their working models based on the currently available sensory and perceptual information, and the new working model would be effective in processing the newly present information^22^. This model focuses on the segmentation of various events and the cognitive processes involved in the transformation between events.

The current study investigated how tension experience was modulated by music developmental patterns, through comparing tonal shift (TS) and melodic shift (MS) conditions with repetition condition (RP). In music composition theory, it is established that changes in tonality and melodic patterns are common manipulations to intensify tension and to convey complex meaning, emotion and even aesthetic evaluation^23–28^. Phrases selected from the classical music repertoire were repeated twice to serve as the baseline condition. Then, the key of the first phrase under the repetition condition was shifted into another key, serving as the TS condition. The materials under the MS condition were original periods, consisting of two successive phrases with different melodic patterns. Since the second phrases under the three conditions were identical, variations in tension experiences during the second phrases would reveal the effect of developmental patterns on music tension. Real-time ratings of the tension experiences were recorded. Using EEG technique, we explored the electrophysiological mechanism of tension experiences induced by different developmental patterns.

Tonal shift may increase tension experience, and thus provides yearning of returning to the original key^2,26^. Sun et al.^4^ found that higher tension experience was induced by tonal shift at phrase and period boundaries, with larger N5 component and decreased alpha band power induced at period boundary than at phrase boundary. This suggested that tonal shift between music events affected tension experiences. As mentioned above, most studies tried to reveal the tonal violation processing in the realm of musical syntax, it still needs further research to uncover the effect of tonal shift in music event structure on tension experiences.

For melodic prediction, Miranda and Ullman^29^ investigated the familiarity effect in melodic prediction and found that violations of melodic prediction (unexpected but in-key notes) induced larger right anterior negativity (RAN) and P600 in familiar melodies than those in unfamiliar melodies, which suggested that attentional reorientation and integration processing might be involved in melodic prediction. A follow-up analysis of that study demonstrated that the P3a component was induced by out-of-the-key notes in the melodic sequences. Similarly, Calma-Roddin and Drury^30^ found that ERAN was delayed in unfamiliar melodies compared with familiar ones, and Fogel et al.^31^ found that melodic prediction depended on local pitch intervals and large-scale melodic patterns by using a cloze probability task. These findings provide solid proof that prediction of musical features (tonal and melodic) plays a vital role in music perception. However, the relations between music tension experiences and the shift of features in music sequences remain unsettled.

We hypothesized that changes in tonality and melodic patterns increase tension experiences and that the time courses and neural oscillations of the tonal and melodic shift effects to be different. Tonal and melodic shift would induce higher tension experience than the RP condition with distinct mechanisms due to their differences in cognitive load (e.g., working memory load) under these conditions. For EEG results, we predicted that tonal shift would induce P3a for tonal shift detection and P600/LPC for integration processing of the incongruent tonality during the two phrases. Other components such as RAN/ERAN might be less likely induced because RAN/ERAN would usually be induced by unexpected and in-key events. Meanwhile, under MS condition, change of rhythmic-melodic features would induce RAN/ERAN due to prediction violations, and P600/LPC due to integration of the two melodic patterns. The detection of rhythmic-melodic feature violation might be earlier than that of tonal violation.

In addition, we also suspected the likelihood of the N5 component under the TS condition and the N400 component under the MS condition. Previous research demonstrated that unexpected chords in harmonic progression induced N5 as a marker for tonal and harmonic structure processing^8,12,15^. However, Sun et al.^4^ also found N5 at phrase and period boundaries with tonal shift, and the authors deemed that N5 indicated both prediction violations and integration processing of incongruence. Therefore, we could not rule out the possibility of N5 under the TS condition. For the MS condition, the N400 component was also an alternative. N400 was induced by melodic prediction violation in the studies by Miranda and Ullman^29^ and Calma-Roddin and Drury^30^. The two studies used familiar melodies and explained that the music-induced N400 might indicate access/retrieval of long-term memory and integration of incongruent melodies. Even though our materials are different, we still cannot exclude the possibility of N400 under MS condition.

## Results

### Behavioral results

**Figure 1:**
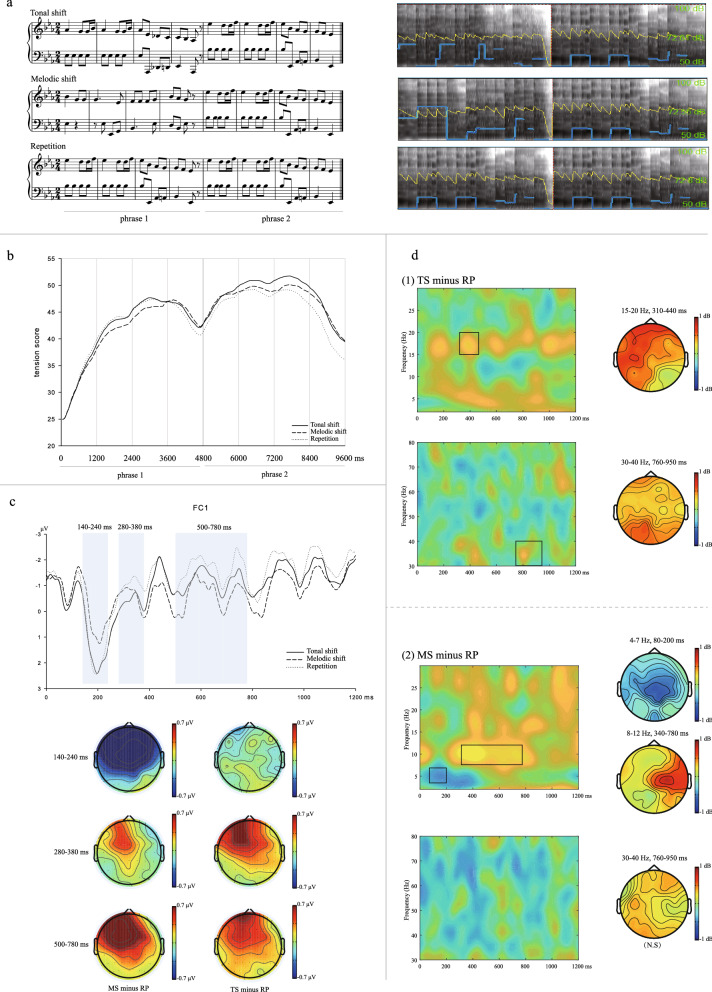
(**a**) Examples of scores (right) and spectrums (left) of the music sequences. (**b**) Mean tension curves under three conditions. (**c**) Grand-average ERP waveforms at FC1 for three different developmental patterns. The map shows the distribution of the effect (MS minus RP and TS minus RP) in the time windows of 140–240 ms, 280–380 ms and 500–780 ms, respectively (top) and the topographies at corresponding time windows (bottom). (**d**) (1), Difference of ERSP of TS minus RP condition at 4–30 Hz (top) and 30–80 Hz (bottom) bands at F3 electrode, with topographies in the corresponding band frequencies and time windows (right); (2), Difference of ERSP of MS minus RP condition at 4–30 Hz (top) and 30–80 Hz (bottom) bands at C3 electrode, with topographies in the corresponding band frequencies and time windows (right). Black squares represent where difference was significant. (Note that no effect was found in 30–40 Hz during the time window of 760–950 ms, therefore no black square was labeled).

The behavioral data selection was based on the criterion as follows: (1) data of four participants were excluded because they did not follow the experimental instructions, with tension curves in more than half of the trials were in a slope or straight line pattern without apparent dynamic fluctuations; (2) the trials (not exceeding half of the total trials of each person) in which the tension curves not have apparent fluctuations within one phrase were excluded; (3) data deviated more than 3 SD at each time point (192 time points each trial) were excluded. The remaining data were averaged across participants under the three conditions at each time point. Figure 1b shows the average tension curves under the three conditions.

This study aimed to test the dynamic change in tension experiences induced by the second phrase under various developmental patterns following the first phrase; thus, the averaged tension scores during the time windows of each beat (600 ms) of the second phrase were analyzed using one-way ANOVA, taking the developmental pattern as a within-subject factor. The results showed that the main effect of developmental pattern was significant during all time windows except the first measure of the second phrase: mm1beat1 (first beat of the first measure), *F* (2, 60) = 1.901, *p* = 0.322, *η*_*p*_^*2*^ = 0.050; mm1beat2, *F* (2, 60) = 1.436, *p* = 0.392, *η*_*p*_^*2*^ = 0.058; mm2beat1, *F* (2, 60) = 3.984, *p* = 0.023, *η*_*p*_^*2*^ = 0.134; mm2beat2, *F* (2, 60) = 7.012, *p* = 0.002, *η*_*p*_^*2*^ = 0.217; mm3beat1, *F* (2, 60) = 8.809, *p* < 0.001, *η*_*p*_^*2*^ = 0.263; mm3beat2, *F* (2, 60) = 11.360, *p* < 0.001, *η*_*p*_^*2*^ = 0.295; mm4beat1, *F* (2, 60) = 15.112, *p* < 0.001, *η*_*p*_^*2*^ = 0.362; mm4beat2, *F* (2, 60) = 17.674, *p* < 0.001, *η*_*p*_^*2*^ = 0.391.

Post hoc tests were further performed at each time window of eight beats of the second phrase. The results showed that during the time window of the first beat of the first measure, the difference in the ratings between the MS and RP conditions was not significant, *p* = 0.142, nor was the difference between the TS and RP conditions, *p* = 0.187; during the second beat: difference of the ratings between MS and RP conditions was not significant, *p* = 0.335, but was significant between the TS and RP conditions, *p* = 0.271. During the two beats of the second measure, the difference in ratings between the MS and RP conditions was not significant, *p*s ≥ 0.232, but was significant between the TS and RP conditions, *p* = 0.021 and 0.007 respectively. During the third measure, the difference in the ratings between the MS and RP conditions was not significant during the first beat, *p* = 0.379, but significant during the second beat, *p* = 0.029; the difference of the ratings between TS and RP condition was significant during both beats, *p*s < 0.001. During the fourth measure, the difference in the averaged tension score between the MS and RP conditions was significant during both beats, *p*s < 0.001, as was the difference between the TS and RP conditions, *p*s < 0.001. All the *p values* were FDR-corrected and are the same hereinafter.

We also conducted paired comparisons between the ratings of the second phrases under the two tonal modulation directions. Since we manipulated the register of the first phrases while the register of the second phrases was similar, the tension scores of each beat between the two conditions were not significant, *p*s ≥ 0.402. Moreover, to rule out the effect of the first phrase on the second one, we further analyzed the ratings of the last measure during the first phrase and conducted one-way ANOVA, with developmental pattern being an independent variable. The results showed that the main effect of developmental pattern is not significant, *F*s (2, 60) ≥ 0.71, *p*s ≥ 0.534, *η*_*p*_^*2*^s ≥ 0.031.

### ERP results

The grand average of ERPs in the time window of 0–1200 ms (first measure of second phrase) of 31 participants under three conditions is presented in Fig. 1c.

In the time window of the ERAN component (140–240 ms), the main effect of developmental pattern was significant, *F* (2, 62) = 7.246, *p* = 0.002, *η*_*p*_^*2*^ = 0.206; the interaction between developmental pattern and anteriority was significant, *F* (4, 124) = 5.867, *p* < 0.001, *η*_*p*_^*2*^ = 0.173; the interaction between developmental pattern and hemisphere was marginally significant, *F* (4, 124) = 2.001, *p* = 0.099, *η*_*p*_^*2*^ = 0.067; the interaction between developmental pattern, hemisphere and anteriority was significant, *F* (8, 248) = 2.501, *p* = 0.013, *η*_*p*_^*2*^ = 0.082; and the main effect of hemisphere and anteriority and their interaction were all significant, *F*s ≥ 5.234, *p*s < 0.001, *η*_*p*_^*2*^s ≥ 0.157. Specifically, a three-way ANOVA was performed at each ROI, and the results showed that, the main effect of developmental pattern was significant at all frontal and middle ROIs, *F*s ≥ 5.166, *p*s ≤ 0.009, *η*_*p*_^*2*^s ≥ 0.156. Paired comparison results showed that the difference between the mean amplitude of the RP and MS conditions was significant at all frontal and middle ROIs, *p*s ≤ 0.017, but not significant between the RP and TS conditions, *p*s ≥ 0.535.

In the time window of 280–380 ms, the main effect of developmental pattern was not significant, *F* (2, 62) = 1.412, *p* = 0.252, *η*_*p*_^*2*^ = 0.048; the main effect of hemisphere was significant, *F* (2, 62) = 3.849, *p* = 0.027, *η*_*p*_^*2*^ = 0.121; the interaction between hemisphere and anteriority was significant, *F* (4, 124) = 5.541, *p* < 0.001, *η*_*p*_^*2*^ = 0.165; the interaction between developmental pattern, hemisphere and anteriority was marginally significant, *F* (8, 248) = 1.793, *p* = 0.079, *η*_*p*_^*2*^ = 0.060; and no other main effect or interaction was significant, *F*s ≤ 1.884, *p*s ≥ 0.118, *η*_*p*_^*2*^s ≤ 0.063. Paired comparison was performed at each ROI, and the results showed that the difference between the RP and TS conditions was significant at frontal ROIs and marginally significant at the central-middle ROI, *p*s ≤ 0.055, but the difference between the RP and MS condition was not significant at all ROIs, *p*s ≥ 0.171.

In the time window of LPC (500–780 ms), the main effect of developmental pattern was significant, *F* (2, 62) = 4.834, *p* = 0.011, *η*_*p*_^*2*^ = 0.135; the main effect of anteriority was significant, *F* (2, 62) = 9.334, *p* < 0.001, *η*_*p*_^*2*^ = 0.231; the main effect of hemisphere was significant, *F* (2, 62) = 10.529, *p* < 0.001, *η*_*p*_^*2*^ = 0.254; the interaction between developmental pattern and anteriority was significant, *F* (4, 124) = 4.013, *p* = 0.004, *η*_*p*_^*2*^ = 0.115; the interaction between developmental pattern and hemisphere was significant, *F* (4, 124) = 2.756, *p* = 0.031, *η*_*p*_^*2*^ = 0.082; and no other effects were significant, *F*s ≤ 0.414, *p*s ≥ 0.912, *η*_*p*_^*2*^s ≤ 0.013. Paired comparison was performed at each ROI, and the results showed that the difference between the RP and MS conditions was significant at all frontal ROIs and in the left-middle and central-middle areas, *p*s ≤ 0.033, and the difference between the RP and TS conditions was significant in the left-frontal, middle-frontal and central-middle areas, *p*s ≤ 0.048.

### ERSP results

Figure 1d shows the differences in ERSP between the TS minus RP condition and the MS minus RP condition in the time window of the first measure of the second phrase (0–1200 ms).

As it can be inferred that the effects induced by tonal and melodic shift may be different, in the analysis of ERSP data, we performed only paired comparisons using permutation tests between the TS and RP conditions and between the MS and RP conditions separately, rather than three-way analysis.

Permutation tests showed that, compared with the RP condition, the TS condition induced beta-band power increases (*p* = 0.032, 15–20 Hz) in the time window of 310–440 ms after the onset of the second phrase and gamma band power increases (*p* = 0.039, 30–40 Hz) in the time window of 760–950 ms. However, no effect was found in these frequencies and time windows between the MS and RP conditions. Meanwhile, compared with the RP condition, the MS condition induced theta band power decreases (*p* = 0.034, 4–7 Hz) in the time window of 80–200 ms after the onset of the second phrase, and alpha band power increases (*p* = 0.028, 8–12 Hz) in the time window of 340–780 ms. No effect was found in these frequency bands and time windows between the TS and RP conditions.

## Discussion

This study investigated the effects of developmental patterns between two successive phrases on tension experiences and the underlying cognitive mechanisms. It was found that the second phrases under both the TS and MS conditions induced higher tension experiences than the RP condition. The tension ratings of the second phrases under the TS condition were higher than those under the other two conditions, with the lowest under the RP condition. EEG responses induced by these developmental patterns had different time courses and neural oscillations. Melodic shift induced ERAN and LPC, while tonal shift induced P300 and LPC. Moreover, the former induced decreased theta and increased alpha band power, while the latter induced increased beta and gamma band power. It is suggested that different developmental patterns affected tension experience but with different underlying cognitive neural mechanisms. As proposed by McAdams and Bigand^13^ and Lerdahl^14^, the TS and MS conditions could be regarded as two types of weak prolongation connections, while the RP condition was a strong prolongation connection. Listeners established an event model of tonal or melodic features based on the context of preceding phrases and predictions upon subsequent music events, and prediction violations would induce higher tension.

### Tonal shift effect on music tension experience

Compared with the RP condition, tonal and melodic change induced higher tension ratings nearly from the second measure, rather than the beginning of the first measure of the second phrases. Tension ratings of the 2–4 measures during the second phrase under the TS condition were the highest among the three conditions, and the difference remained toward the ending point of music piece. Moreover, the slope of the growth curve was higher than that under the other conditions. In our study, the two phrases under the RP condition were identical except that the ending of the first phrase contained a 300-ms rest to intensify the saliency of phrase boundaries. Therefore, tonal modulation might be the main reason for the increased tension. Lehne et al.^17^ found that when listening to sonatas, higher tension experiences were associated with tonal modulations, while lower tension with repetition of the same chords. Our findings were consistent with those of previous research. Repetition of the same element, whether a chord or phrase, is easy to process and induces less anticipation or emotional experience. In contrast, tonal shift of the same phrase induces higher tension, even when the rhythmic-melodic features of the two phrases are identical. It was not difficult for the musicians to recognize that the two phrases were identical except the tonality, but it still induced the highest tension experience, which indicated that tonal shift was an effective developmental pattern to intensify the emotional expression of music.

Violation of tonal prediction leads to increased tension ratings both at the induction and resolution stages. The study on nested structure^8^ found a tonal shift effect, but no evidence of the EEG data at the tonal shift boundaries, where tension curves under different conditions started to separate. Rather, in the time window of the ending chords (resolution stage), different nested structures showed different LPC amplitudes, where tension ratings showed no difference. The underpinning cognitive processing mechanism at the crucial time points of tonal shift is still unclear. Therefore, our study locked the time windows of EEG analysis to the onset of the second phrases to provide direct evidence for the tonal shift effect on tension experiences. Thus, this study made a crucial contribution to elucidating tension processing at the induction stage. Together with previous findings, a more complete picture of the mechanism of tension experience at the induction and resolution stages was presented.

Early detection of tonal change induced P300, which is a typical component in the odd-ball paradigm and is associated with a low probability of stimulus and task relevance^32^, and it reflects attentional orienting toward the stimuli^33,34^. Music perception studies found that P300 was induced by unpredicted cadences or incongruent tonal context^35–37^. In our study, the listeners perceived the first phrase in a stable tonal feature for 4.8 s; therefore, tonal shift in the second phrase also induces a prediction error. More attentional resources need to be allocated to process inconsistent tonality. After detection of the violated tonality, listeners integrate the current tonality with the first phrase. LPC is an ERP component correlated with integration processing^8,38,39^. The relationship between the two phrases would be evaluated from the onset of the second phrase. Consistent with previous findings^8^, integration processing of the tonality induced LPC at a late stage. It is worth noting that our study focused on prediction violations of the relationships between phrases rather than harmonic structures. Therefore, no typical N5 component was found. The results of Sun et al.^4^ showed that N5 may be induced by tonal shift at phrase boundaries could because the materials were self-written chords sequences, and listeners might analyze the harmonic structure rather than the event structure. Our results proved that the cognitive mechanisms of tonal modulations within the harmonic structure and event structure were different.

The ERSP results showed that tonal shift induced increased beta and gamma band power with topographies of right lateralization. Beta power increase might reflect prediction processing. In the study of Chang, Bosnyak and Trainor^40^, unpredicted deviant pitch induced increased beta at approximately 200–300 ms with right lateralization, which suggested the significance of beta oscillations for sensory prediction. Our task was more difficult, leading to a later time window (310–440 ms) for the effect. Late gamma band oscillation is usually concerned with task difficulty and prediction violations. In the study of Weiss et al.^41^, a complex syntactic structure induced increased gamma band power compared with a simple syntactic structure. Similarly, in our study, task difficulty was raised by tonal integration. At the phrase boundary, the prediction model established during the first phrase could not fit the second phrase; therefore, listeners might change their processing strategy from a top-down to a bottom-up^42,43^ pattern.

### Melodic shift effect on music tension experience

Our results showed moderately higher tension ratings under the MS condition. The tonal features of the two phrases were identical, but the melodic and rhythmic features were different (see in “Method”). Therefore, the processing difficulty, especially the working memory load is higher than that under the TS and RP conditions. We speculated that task difficulty inhibited tension experience, thus the tension ratings under MS condition were lower than TS condition. Recognition of melodic patterns may not be easy as on/off beat or tonal perception. The ERAN induced by MS mainly reflected early rhythmic-melodic detection. Researchers considered ERAN to reflect detection of rule-based violations^44^ and the automatic cognitive processing of musical events in the temporal domain^45–48^. In the study of Sun et al.^48^, rhythmic violation was presented by lengthening the penult chord to the last one, thus forming an off-beat condition, and larger ERAN was induced. The brain could make accurate predictions of the upcoming rhythmic event, even when the event was absent^49–52^. When the melodic pattern changes, more attentional resources are required to modulate the representation of melodic pattern established during the first phrase^53,54^.

Additionally, comparison between the different melodic patterns of the two phrases may require more cognitive resources. Consistent with the findings of the study of Sun et al.^47^, where irregular rhythmic patterns induced a larger P600 with left frontal topographic distribution, our results showed LPC with similar topography. LPC/P600 is an ERP marker of integration processing. Processing a new melodic pattern leads to higher working memory load. Therefore, integration of the two melodies elicited a larger LPC. The relationship between the two phrases under the MS condition was relatively complicated due to the change in both rhythmic and melodic features. Evaluation of the relationship between the two phrases would be evaluated in a different way from that in the TS condition. Our results did not find N400 as that in the study of Miranda and Ullman^29^ and Calma-Roddin and Drury^30^. It might be due to the familiarity of the music sequences used in the experiments. Familiar sequences were selected in those studies and unpredicted melodies induced larger N400, and the mechanism of the prediction processing was different from that of the current study, since familiarity was not a manipulated factor for comparison.

ERSP results showed decreased theta band power and increased alpha band power. Frontal oscillations within the theta frequency band are associated with the detection of novelty, conflict, punishment, and error in incoming sensory feedback stimuli, and are an electrophysiological signature of increased demand for top-down cognitive control^55^. The mid-late alpha band power increase, however, might reflect a follow-up processing. Alpha band oscillations are regarded as relevant to attention and working memory. Previous studies found that alpha power was associated with the complexity of music syntactic structures^56,57^ and working memory load^58–60^. Our results are consistent with previous findings. Overall, the melodic shift effect was different from the tonal shift effect not only in the time courses but also in the underpinning cognitive mechanism.

### Event structure processing and music tension

The Event Structure Processing Model^13^ has specified the five cognitive stages of processing musical events. Before the event hierarchy is established, events must be segmented, which might be processed according to Gestalt principles. Those with similar features are more likely to be grouped into one segment. When the tonality or melodic patterns change, Gestalt principles guide listeners to separate them into different events. After segmentation of an event, the time-span reduction proceeds, during which the important elements of the events are extracted and features of the two events are compared or integrated. In our study, LPC could be a marker of time-span reduction processing, since the topographic distributions under MS condition were similar to those under TS condition. Moreover, the ERPS results suggested that the time-span reduction might be complex. Integration processing induced the same ERP component (LPC), but different ERSP effects, which suggested that integration processing of the two developmental patterns might have different cognitive mechanisms. Our results showed that changes in tonal features induced increased beta and gamma oscillations, which mainly reflected prediction processing and integration^40,41^. Meanwhile, changes in rhythmic-melodic features induced decreased theta and increased alpha oscillations, which mainly reflected attentional, working memory and arousal processing^56–60^. Additionally, integration might contain different substages, and further research on the specific processing mechanism of integration is needed.

For the prolongation reduction stage, it is rather late and involves the whole event hierarchy. When processing music in real time, it is possible that prolongation reduction is processed after time-span segmentation and time-span reduction. However, the exact time course of prolongation reduction might not be specified easily because this stage provides an in-depth analysis of the relationships between events within groups, between groups within sections, and between sections within the entire music piece^13,14^. If time-span reduction was to integrate the various features of the two events, then prolongation reduction would integrate the whole music passage. All tension-relaxation patterns were established after integrating all perceived music sequences^13,14^.

Different from previous studies in the realm of music syntactic structure processing, event structure focuses on the relations between events (for instance, phrases or periods). Typical manipulation on syntactic violations is by using unpredicted chords at phrase or period boundaries, which could induce a sudden and local violations. However, prediction violations in event structure would be in a larger scale and the effect on tension experiences could last for the duration of the whole phrases, leading to different processing mechanism from music syntax. Further research is needed to investigate the relationship between music tension and event structure by using more refined and specified materials.

## Limitations

The current study used real music sequences as experimental materials. Compared with previous studies using self-written chord sequences, our ecological validity was higher. However, there were some effects that could not be controlled for or excluded. Since the second phrases were identical under the three conditions, the first phrase had to have different musical features. Therefore, it would be difficult to rule out the effect of the differences in tension experiences during the first phrase on the second phrase. As our tension rating results showed, the first phrase under the MS condition induced lower tension experiences during the second and third measures. Whether the effect of this difference on the processing of the second phrase could be ruled out was unclear. Further research in which different developmental patterns could be designed without inducing varied tension experiences in the musical context is needed.

## Conclusion

This study examined the effect of music developmental patterns on tension experiences. Compared with repetition, tonal shift and melodic shift induced higher tension ratings during the second phrase. EEG results demonstrated the neural mechanisms of tonal and melodic shift effects. Tonal shift elicited P300 and LPC and increased beta and gamma band power, while melodic shift elicited ERAN and LPC, decreased theta band power and increased alpha band power. From the perspective of music event structure processing, our study provided evidence for the effect of developmental patterns on time-span reduction processing. Overall, music developmental patterns play a crucial role in tension-resolution experiences.

## Method

### Participants

Thirty-five musicians participated in the study (Mean_age_ = 19.54 years, SD = 1.42, 14 males). All participants had received at least eight years of professional music training on at least one Western instrument and were highly proficient in Western tonal music. The instruments they played were piano, violin, viola, cello, trumpet, tuba and marimba, and they practiced the instrument for at least one hour each day. All were neurologically healthy and right-handed and had normal or corrected-to-normal vision. Informed and written consent was obtained from the participants before the experiment and they were paid for their participation. The study was approved by the Institutional Review Board of the Institute of Psychology, Chinese Academy of Sciences, and was conducted in accordance with the ethical principles of the Declaration of Helsinki.

### Stimuli

Thirty original music sequences in 2/4 m and major keys were selected from the piano works composed of Haydn, Mozart and Beethoven. Each one was a period containing two phrases, and each phrase contained four measures. The melodic patterns were different between the two phrases, with the rhythmic and pitch features being distinct from each other. Selected sequences were modified under the principles as follows: the key, harmonic progression and the highest and lowest pitch remained original, and other voices were deleted; all appoggiatura, ornament, expressions and articulations were deleted. A modified version of the sequences was used as the materials in MS. The second phrases were repeated twice, as the materials in RP. For TS, the key of the first phrase of the repetition condition was shifted into its dominant key, while the second phrase remained unchanged; thus, the tonal change between the two phrases was from dominant key to tonic key, for instance, from G major to C major or from C major to F major. We calculated the rhythmic density (note number), pitch intervals (sum of intervals of pitch skip of all two successive notes in the melodic voice) and melodic contour variation (frequency of change of ascending or descending direction) of the two phrases under the MS condition. T test results showed that the two phrases had significant differences: rhythmic density, *t* (118) = − 4.93, *p* < 0.001; pitch intervals, *t* (118) =  − 2.79, *p* = 0.006; melodic contour variation, *t* (118) =  − 4.13, *p* < 0.001, which suggested that melodic features of the two phrases were significantly different. Three sequences originating from one piece formed one set of materials, with the second phrases being identical. Thirty sets of sequences were selected, and all 90 sequences were transposed into another key, thus forming 180 sequences in total as the final materials. At the end of the first boundary, there was quaver rest in both voices. Examples of the materials (scores and spectra) are shown in Fig. 1a.

All scores of the 180 sequences were written using Sibelius 7.5 software (Avid Tech. Incorporated) and were exported in mid format. Using Cubase 5.1 software, the mid files were set at a constant velocity of 100 and were archived with a Yamaha piano timbre at a tempo of 100 beats per minute in .wav format, the same method as used by Sun et al.^4,8^. Each sequence lasted 9.6 s, and the quaver rest at the end of the first phrase was 300 ms. All scores of the sequences used in the current study were presented in Supplementary Information.

### Design

This experiment had a one-variable design, with the independent variable being the developmental pattern between the two phrases, and contained three conditions, RP, TS and MS. All 180 trials of the three conditions were presented in a pseudorandom order, following two rules: no more than three trials of the same condition were presented in succession and consecutive trials did not originate from the same original sequence. All trials were arranged in three blocks, each containing 60 trials.

### Apparatus

This experiment was conducted using E-prime Professional Software (Version 2.0) in a quiet, acoustically and electrically shielded room. The participants were tested 70 cm from a 19-in. CRT computer screen. Music sequences were presented through Bose QuietComfort 35 II headphones. The EEG was recorded using AC amplifiers via the Neuroscan 4.5 system (Neuroscan SynAmps).

### Procedure

Real-time and continuous tension experience was recorded through the E-Prime interface, where participants had to move the mouse horizontally while music sequences were played. Tension scores were recorded at a sampling rate of 20 Hz. Before the beginning of each trial, a horizontal sliding bar was presented in the center of the screen, ranging from 0 to 100. The participants had to click the left mouse button, then the music sequences started to play, and the mouse could be moved to the score according to the felt tension when listening to the music sequences. The initial position of the slider was set to 25 to prevent reaching maximal values. Loudness was set by the participants at a comfortable level. Eight sequences were performed as practice procedures before the experimental session to familiarize the participants with the stimuli and procedure. The methods in this study were consistent with those of Sun et al.^4,8^. Between each block of the experimental session, the participants had a three-minute rest.

### EEG recording and analysis

EEG signals were recorded with 64 electrodes secured in an elastic cap according to the extended 10–20 system. The left mastoid was used as a reference. Two electrodes, each placed on the right and left external canthus, were used to record the horizontal EOG (HEOG). The vertical EOG (VEOG) was recorded with the other two electrodes placed above and below the left eye. All of the electrode impedances were kept below 5 kΩ. A bandpass from 0.05 to 70 Hz was applied to amplify the EEG signals. The sample rate was set as 500 Hz.

### ERP analysis

The EEGLAB toolbox, based on MATLAB, was used for the following procedure for preprocessing the EEG signals. All trials of each subject were manually screened to exclude those trials contaminated with eye movement, electrode drifting, amplifier blocking and electromyogram (EMG) artifacts. The signals were filtered with a high-pass cutoff point of 0.1 Hz and a low-pass cutoff point of 30 Hz. We rereferenced the data to the left mastoid, and EEG epochs were extracted for each epoch extending from − 5 s preceding to 1.2 s following the onset of the second phrase (i.e., − 0.2 s preceding to 6 s following the onset of the first phrase). Then baseline correction (200-ms pre-first-phrase being baseline) was applied. Epochs with the criteria of − 100 μv to 100 μv were rejected. Then independent component analysis (ICA) was performed on the segmented data to remove the components with artifacts, such as blink or muscle movements. Manual artifact rejection was then applied again on the remaining epochs to ensure that no contaminated trials remained.

Data from 31 participants were included in the ERP analysis. The remaining epochs (83% of all trials) were resegmented from 0 to 1200 ms after the onset of the second phrase and then averaged by condition. Mean amplitudes under three conditions were extracted in the time window of each 10 ms within the 1200-ms-epoch (0–10 ms, 10–20 ms, 20–30 ms, etc.). Nine ROIs were chosen for subsequent analysis, i.e., left-anterior (F3, F5, FC3), middle-anterior (Fz), right-anterior (F4, F6, FC4), left-central (C3, C5, CP3), middle-central (Cz), right-central (C4, C6, CP4), left-posterior (P3, P5, PO3), middle-posterior (Pz), and right-posterior (P4, P6, PO4). Then, we performed a three-way ANOVA, with three factors being developmental pattern (TS, MS and RP), hemisphere (left, central, and right) and anteriority (anterior, middle, and posterior). The *p*-values of the main effect of developmental pattern and interactions between developmental pattern and hemisphere and/or anteriority were FDR-corrected. Once there were three or more successive time windows obtaining significant main effect or interactions (corrected *p values* < 0.05), there were merged into a larger time window, similar to Zhang and Zhang^61^. By this method, we finally chose three time windows for subsequent analysis, i.e., 140–240 ms, 280–380 ms and 500–780 ms. Three-way ANOVAs were conducted separately for the three time windows on the mean amplitudes (same factors as above), and all *p values* of three three-way ANOVAs were FDR corrected. The Greenhouse–Geisser correction was applied when appropriate.

### ERSP analysis

Event-related spectral perturbation (ERSP) was applied to characterize the cerebral oscillatory patterns. This was performed by convolving the complex Morlet wavelets with the single-trial EEG data, and the low- and high-frequency ranges were computed separately. In the low frequency range, we used wavelets with frequencies ranging from 2 to 30 Hz, in steps of 1 Hz, and with widths ranging from 2 to 8 cycles; in the high-frequency range, we used wavelets with frequencies ranging from 30 to 80 Hz, with widths ranging from 10 to 20 cycles. The convolution window used a 10-ms step size. Power were averaged across trials and normalized as the change relative to the mean power in a baseline interval from − 5100 to − 4700 ms (pre-first-phrase). Then, the data were transformed into decibel scale (10 * log10 of the signal), yielding the ERSP.

A cluster-based random permutation test was conducted in the Fieldtrip toolbox^62^. The permutation test was performed in a step of approximately 50 ms over 60 electrodes. A simple dependent-samples *t test* was performed for every data point, and adjacent samples exceeding a preset significance level (0.05%) were grouped into clusters. Cluster-level statistics were calculated by taking the sum of the *t*-values with each cluster. The Monte Carlo method with 1000 random iterations of shuffling the condition labels was used within subjects to calculate the significance probability of the clusters.

## Supplementary Information

Supplementary Information 1.

Supplementary Information 2.

Supplementary Information 3.

## Data Availability

The datasets analyzed in the current study are available from the corresponding author on reasonable request.
