# Supplementary figures and images for: Tension experience induced by tonal and melodic shift at music phrase boundaries
Source: Sci Rep. 2022 May 18;12:8304. doi: 10.1038/s41598-022-11949-4 (PMC9117266; doi:10.1038/s41598-022-11949-4)

1


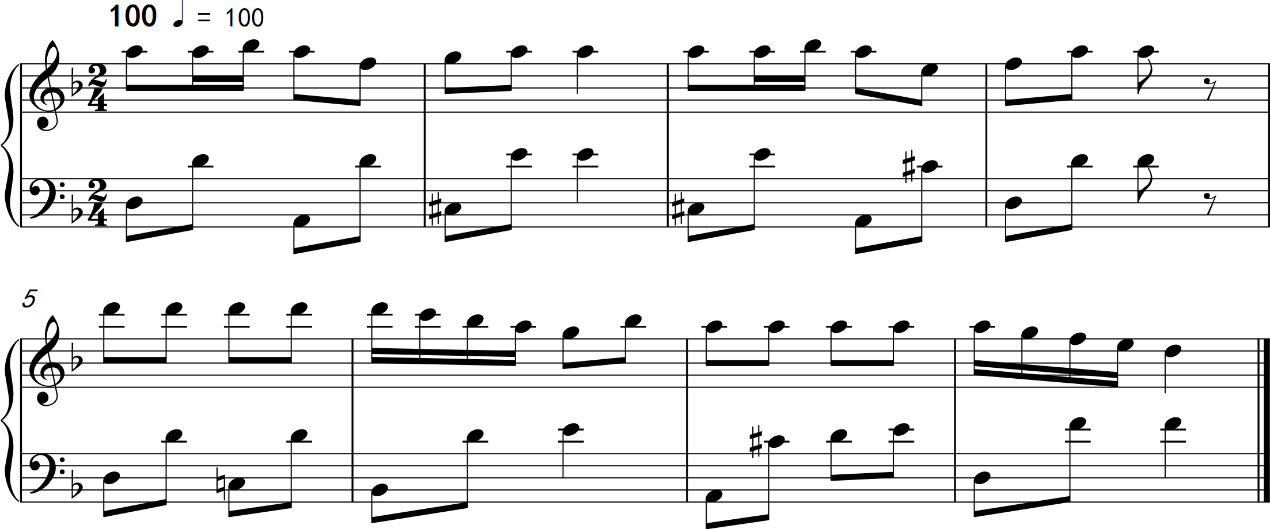


2
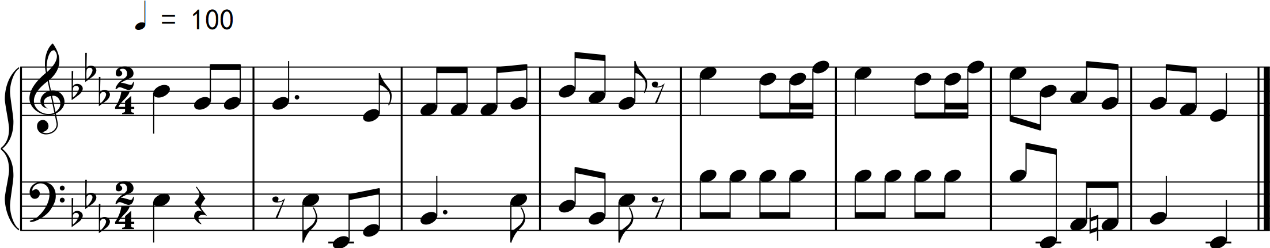
3
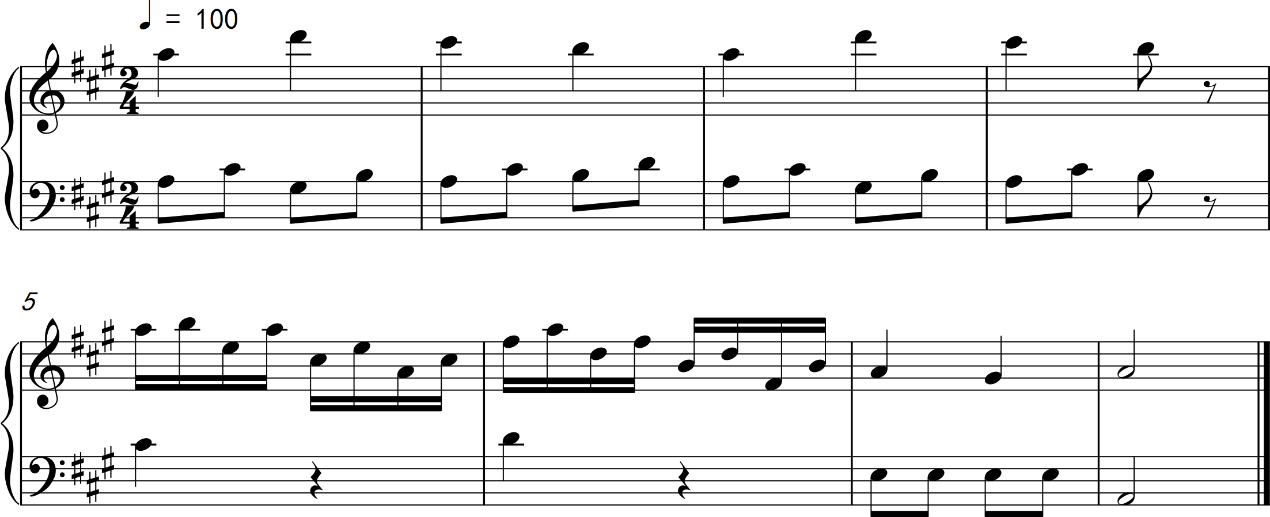
4
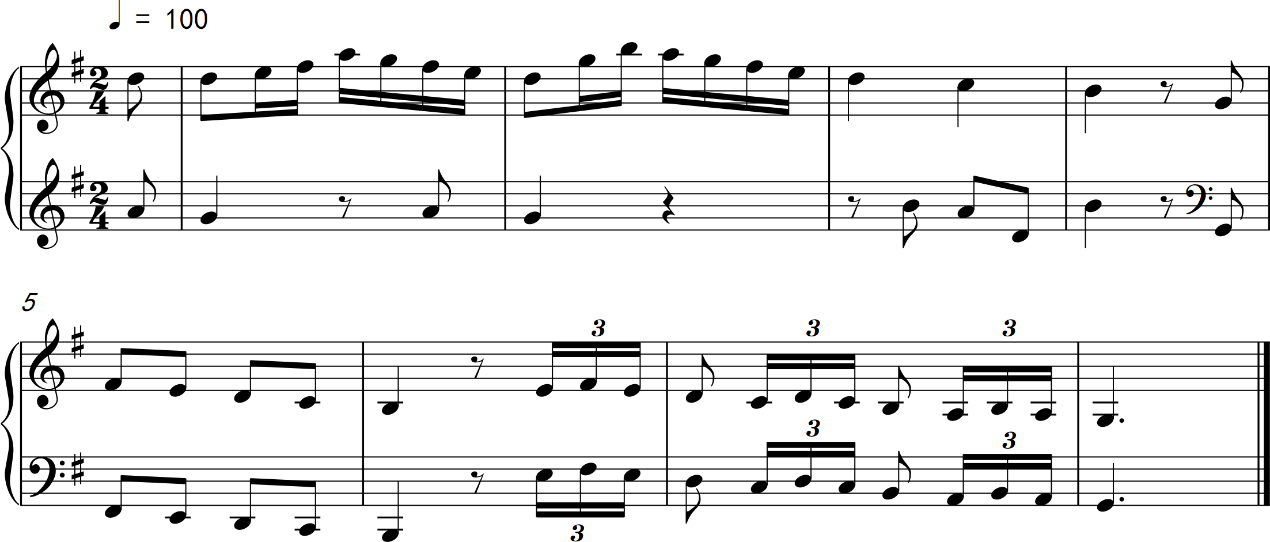
5
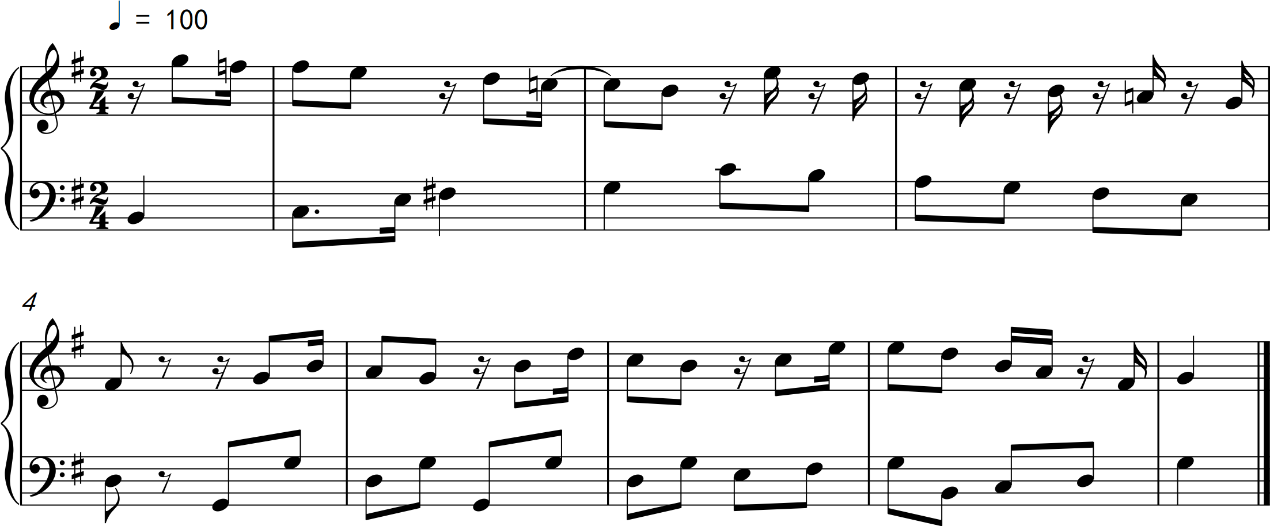
6
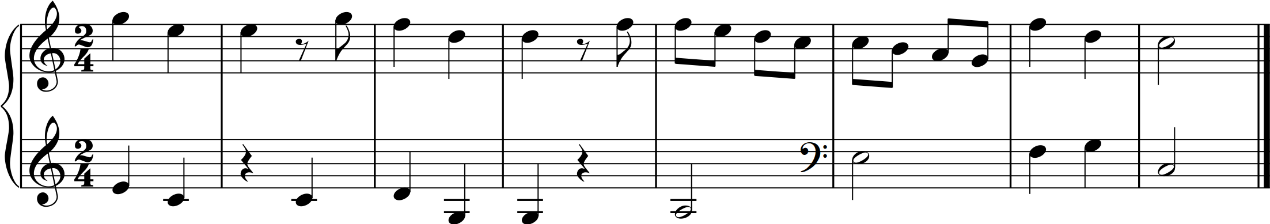
7
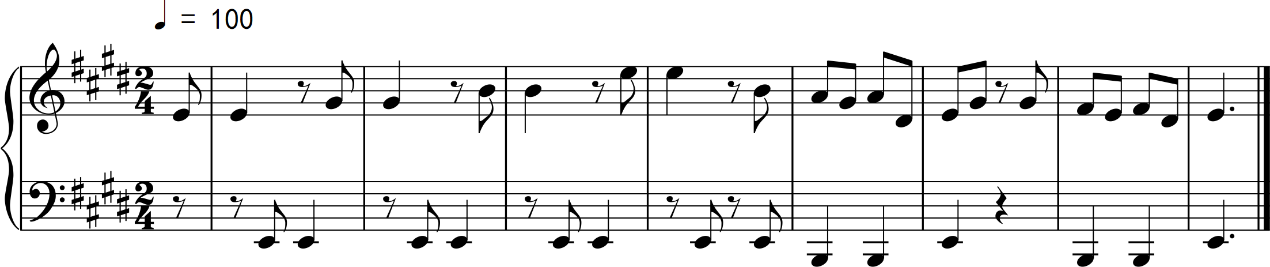
8
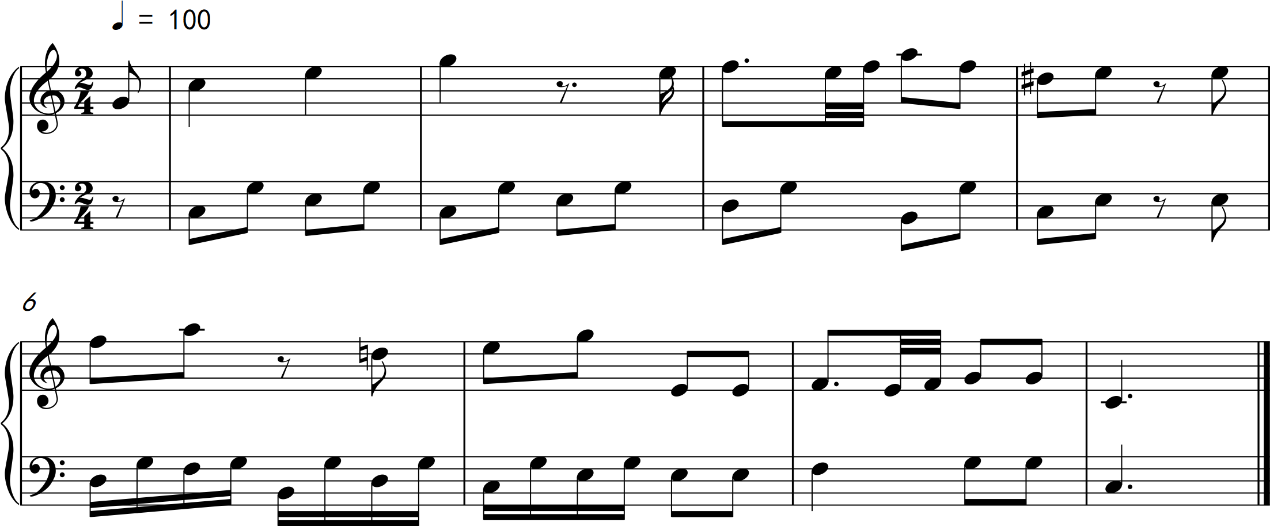
9
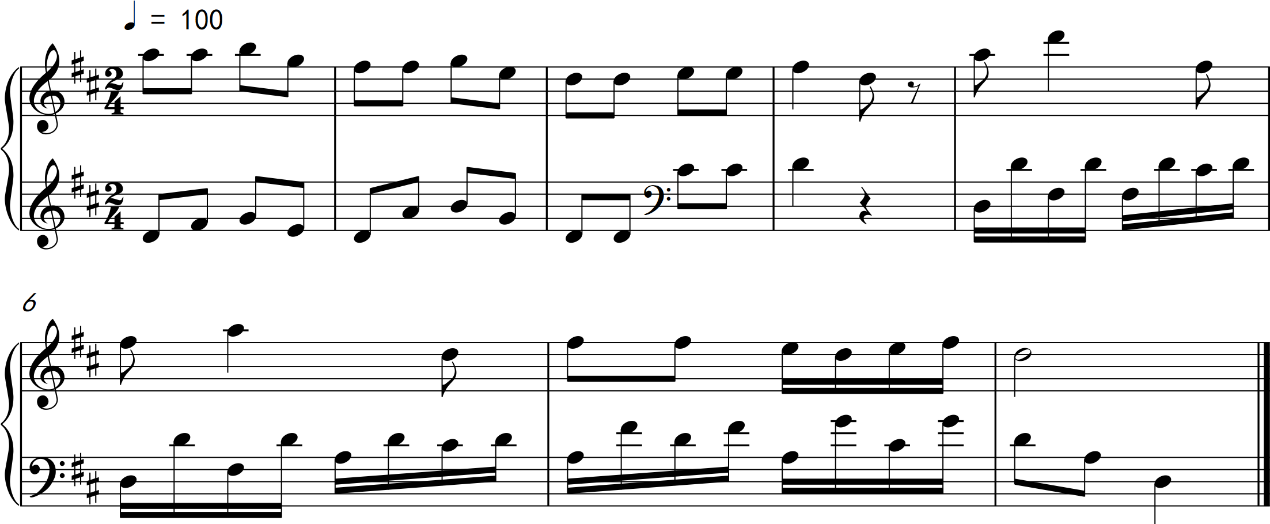
10
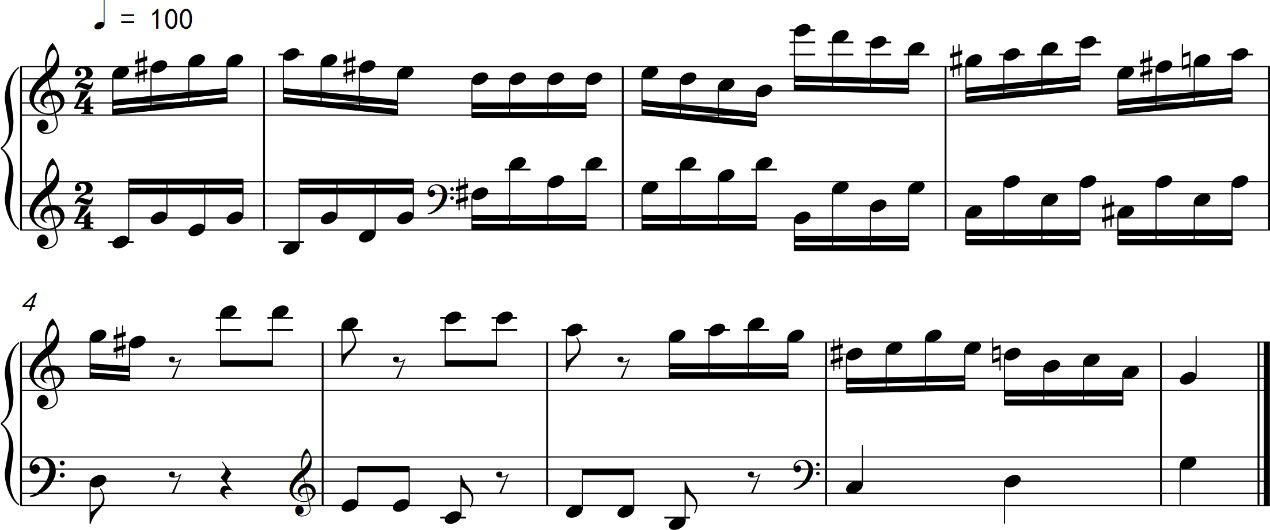
11
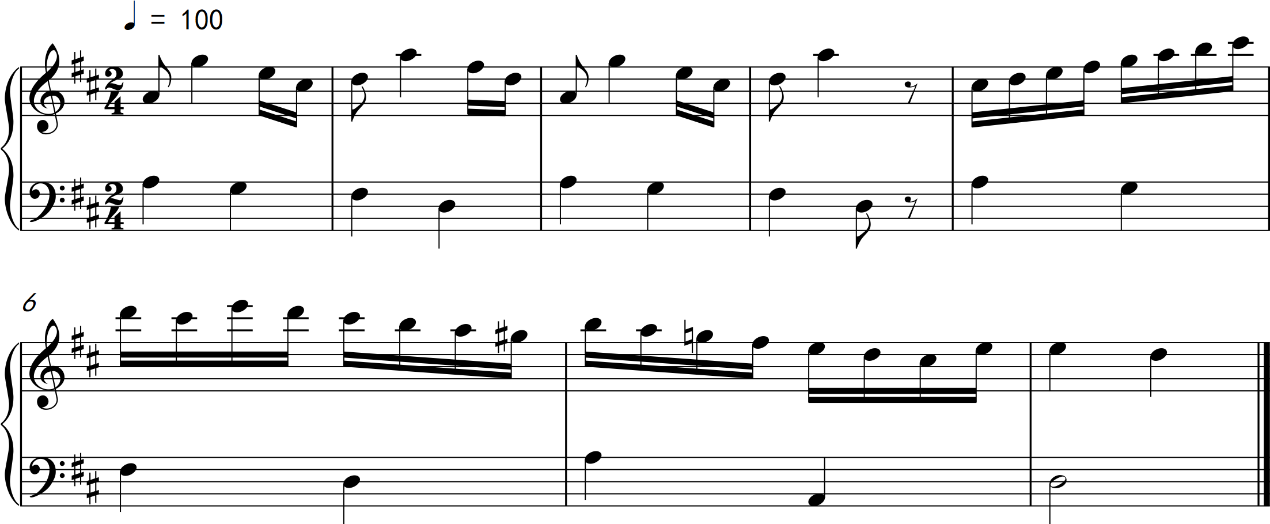
12
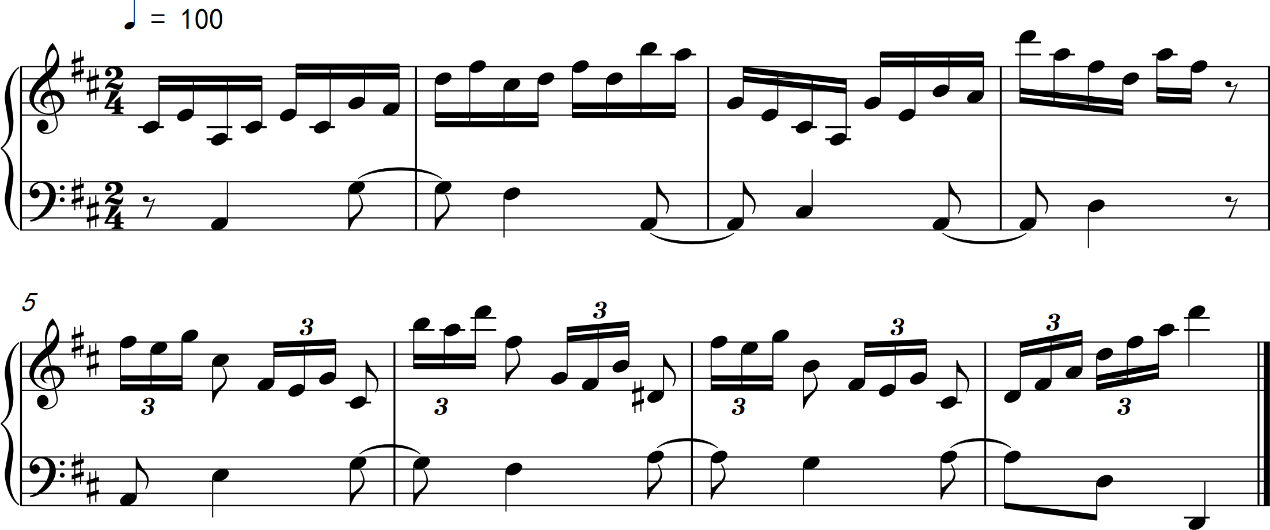
13
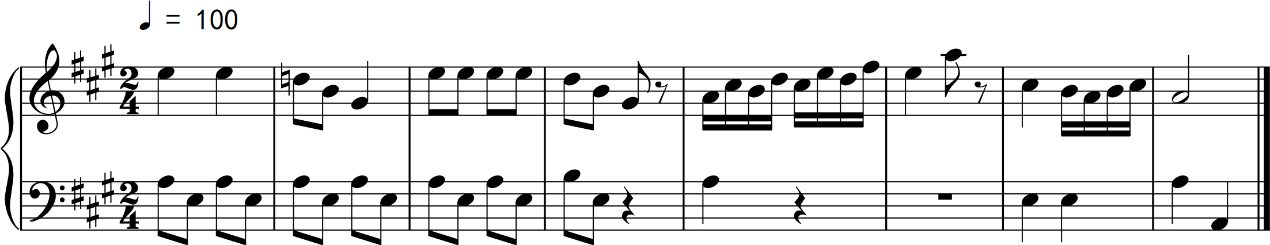
14
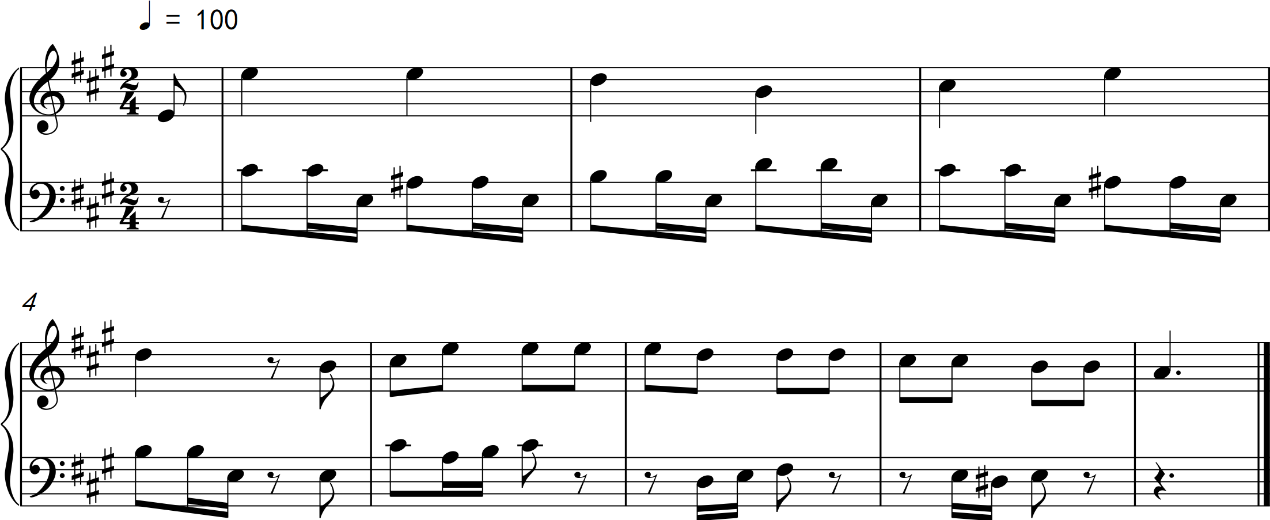
15
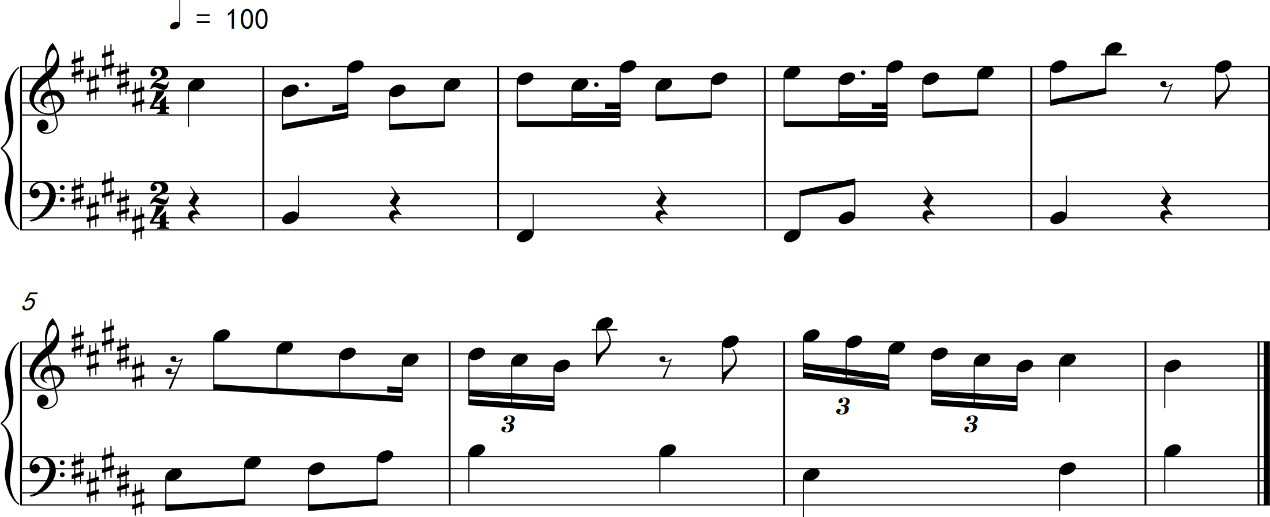
16
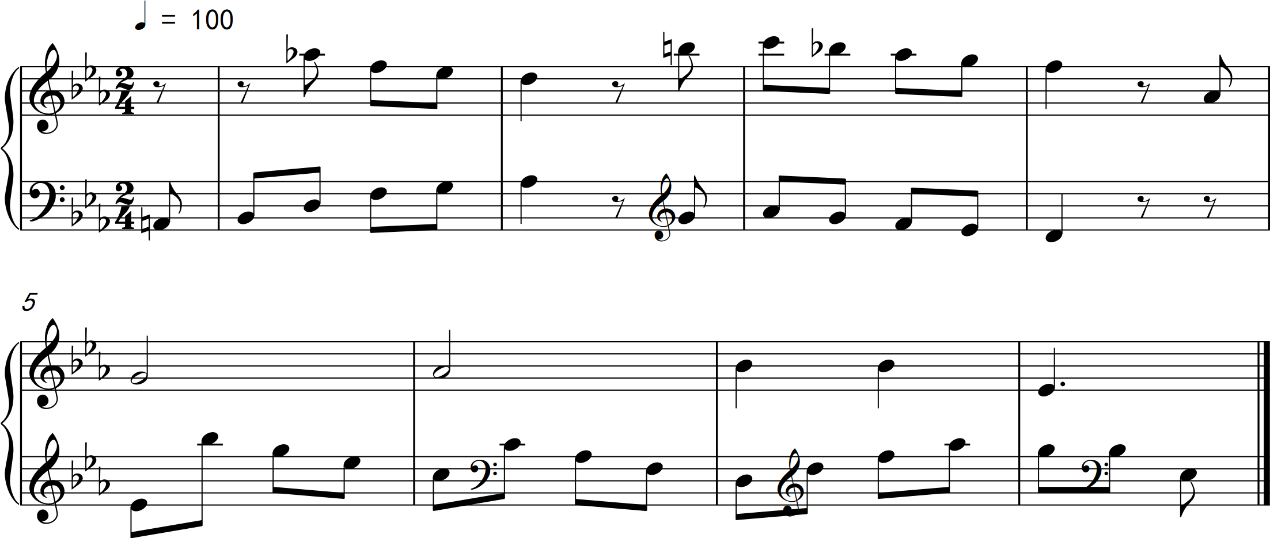
17
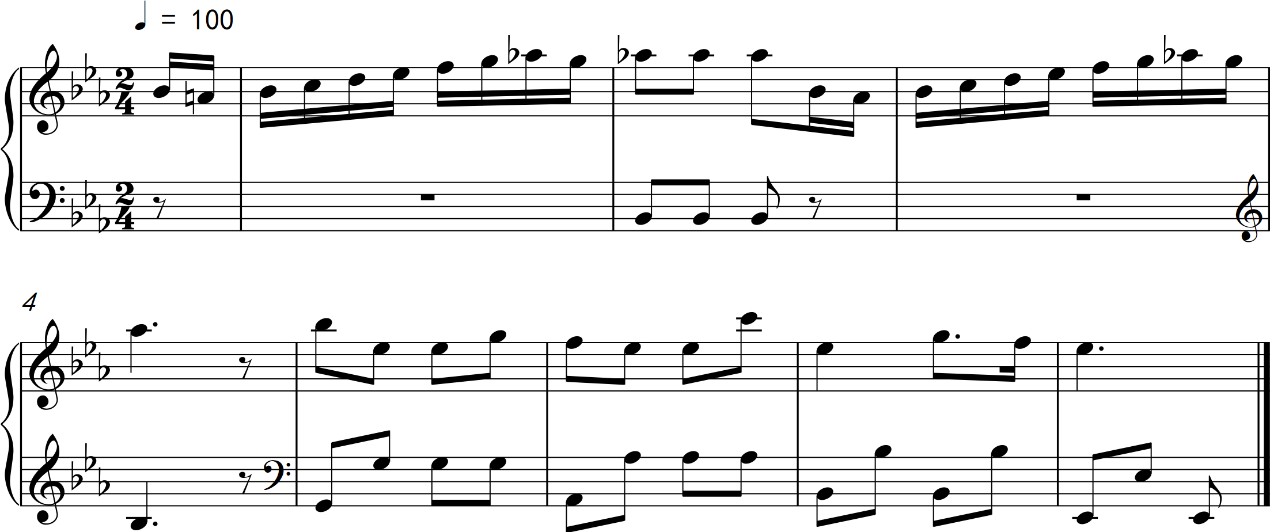
18
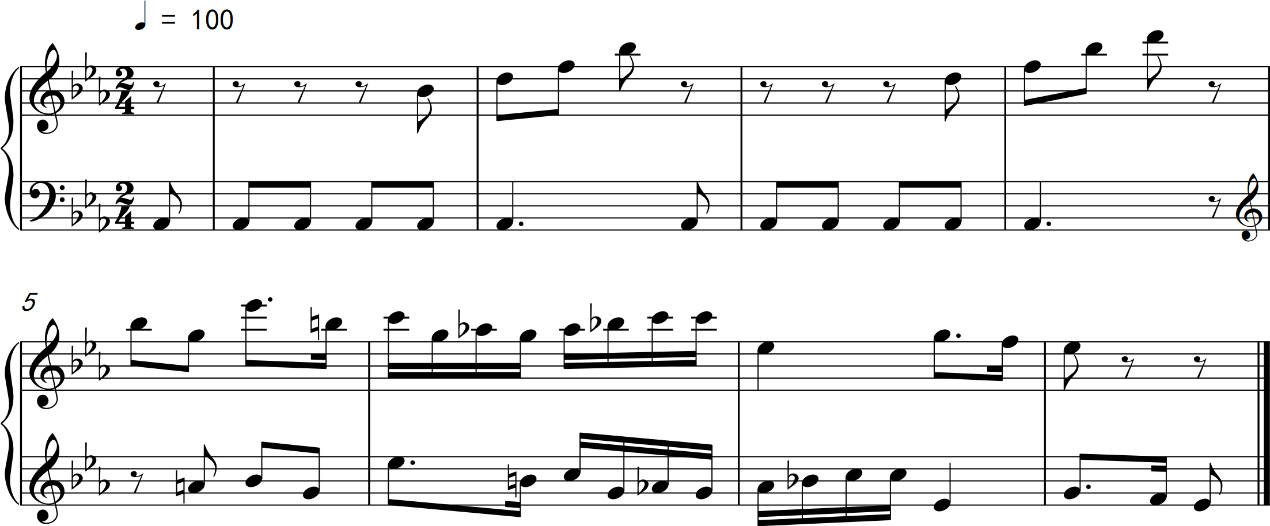
19
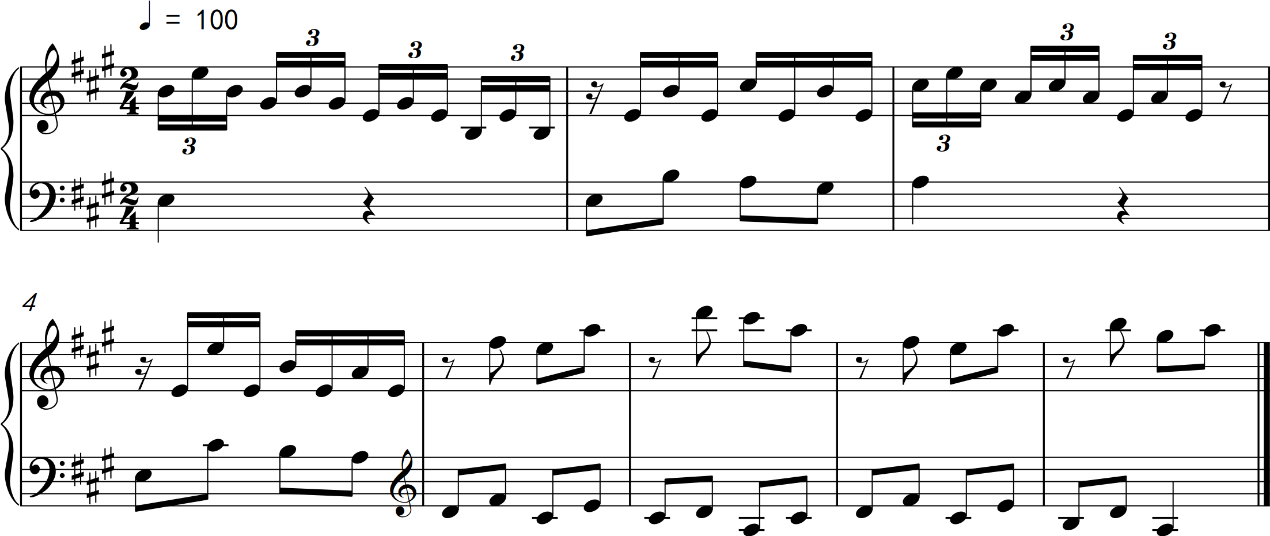
20
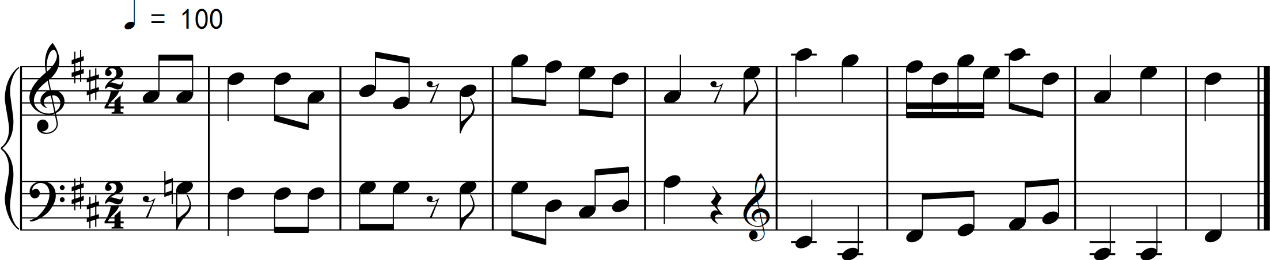
21
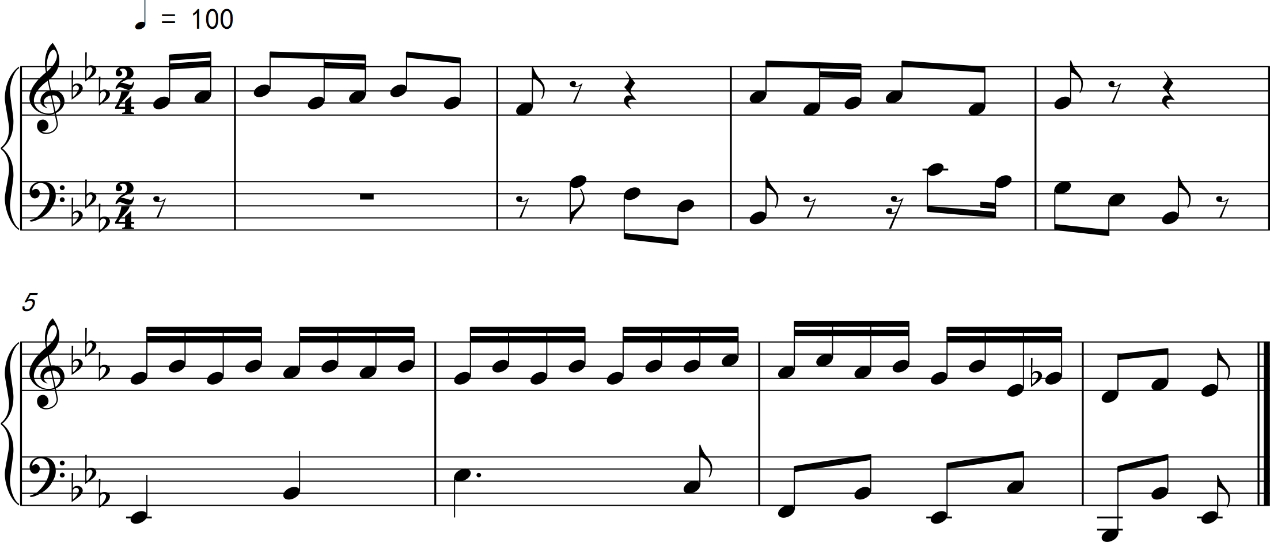
22
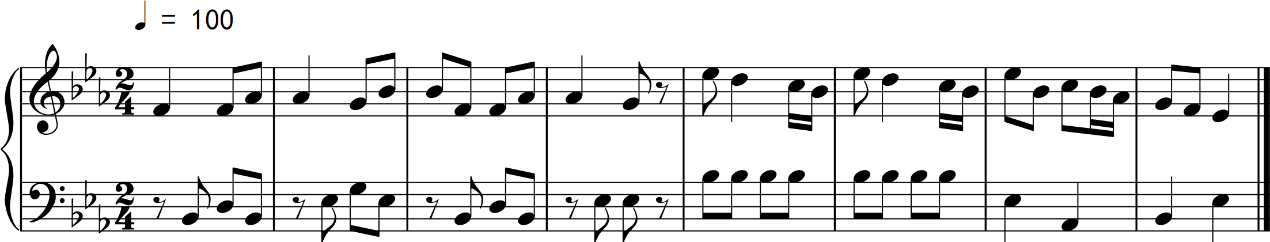
23
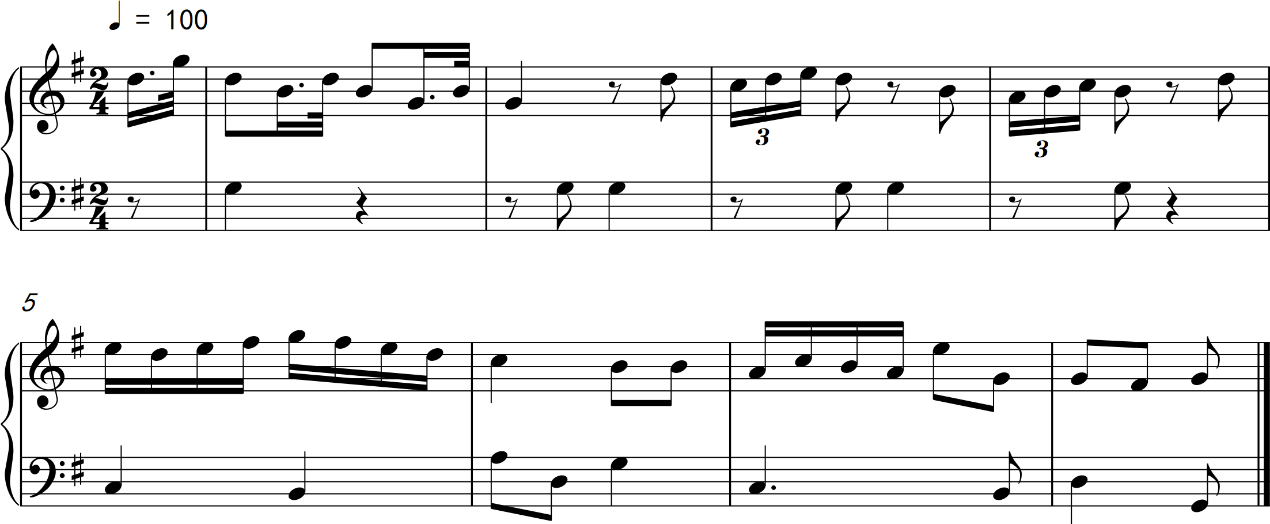
24
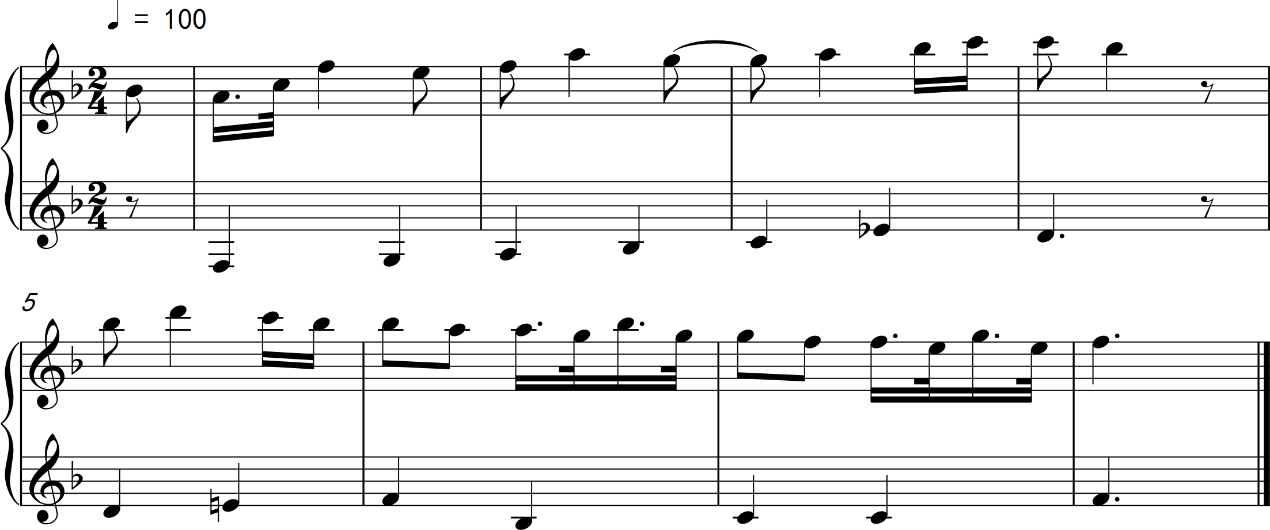
25
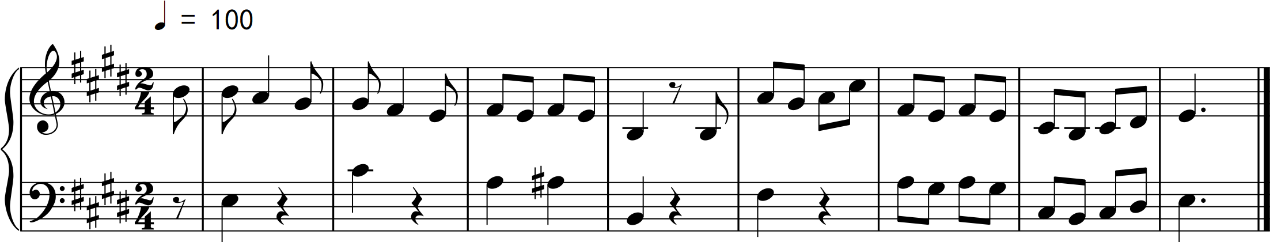
26
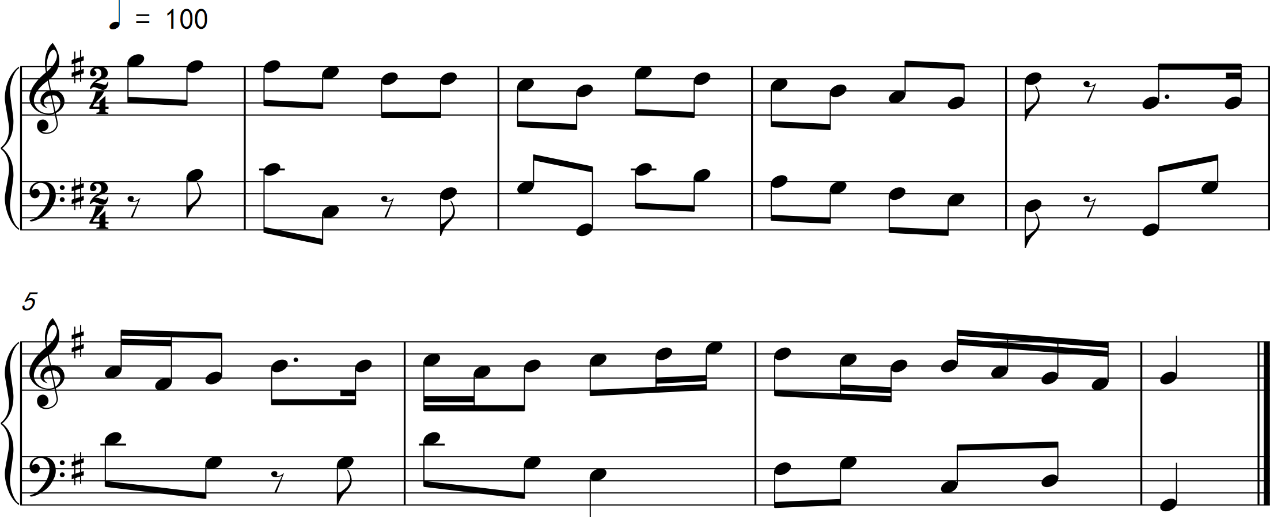
27
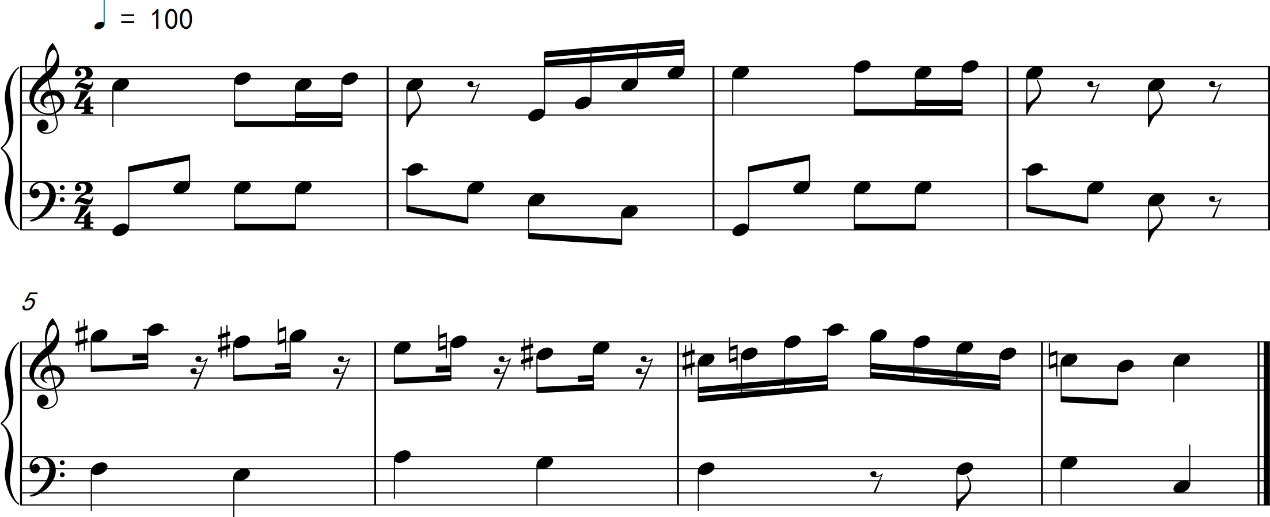
28
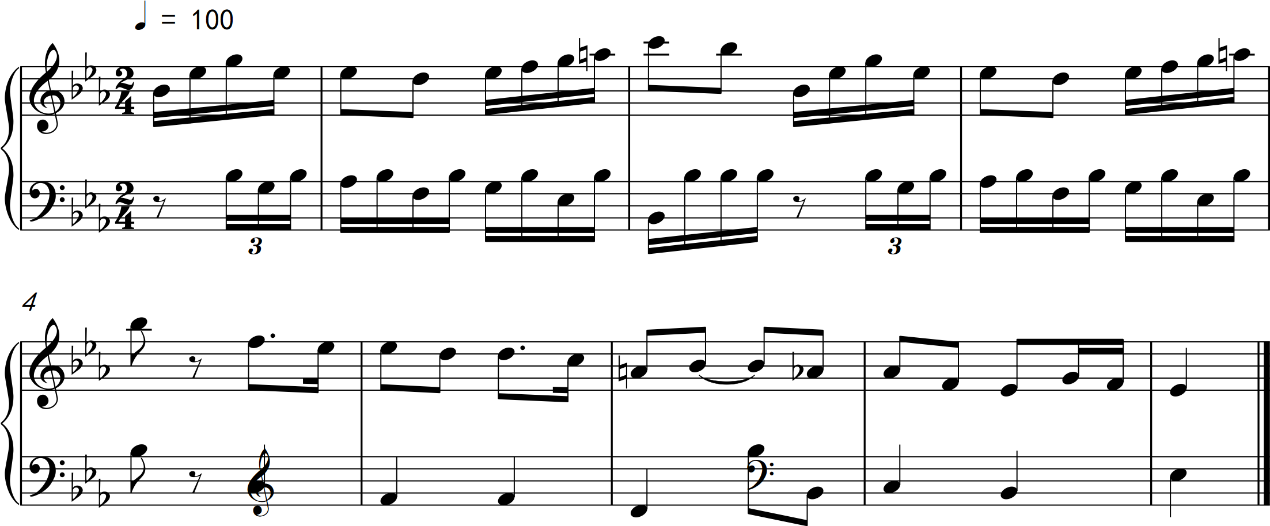
29
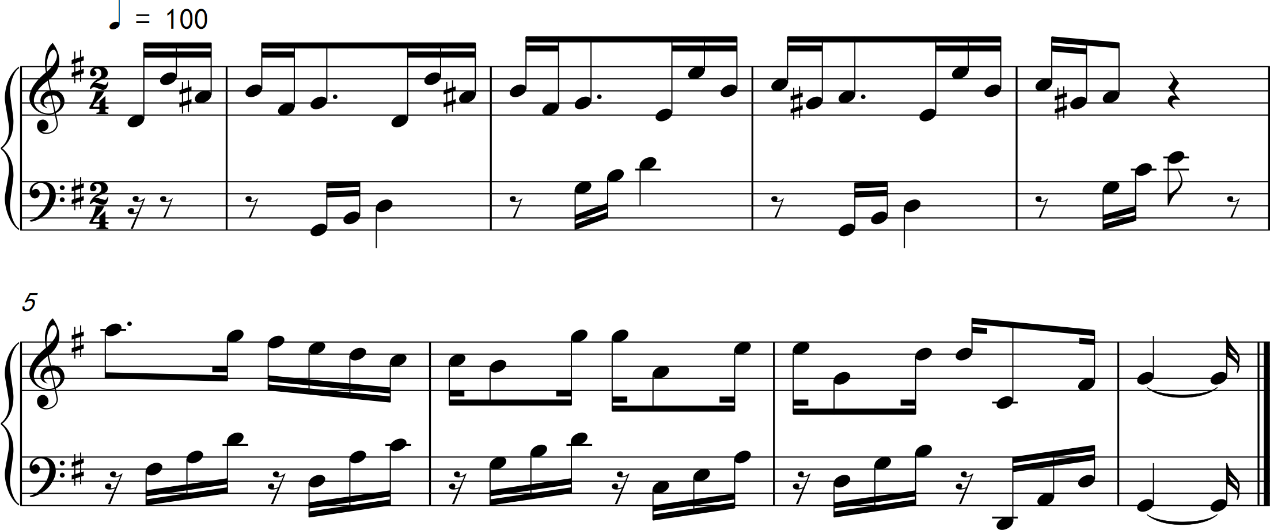
30
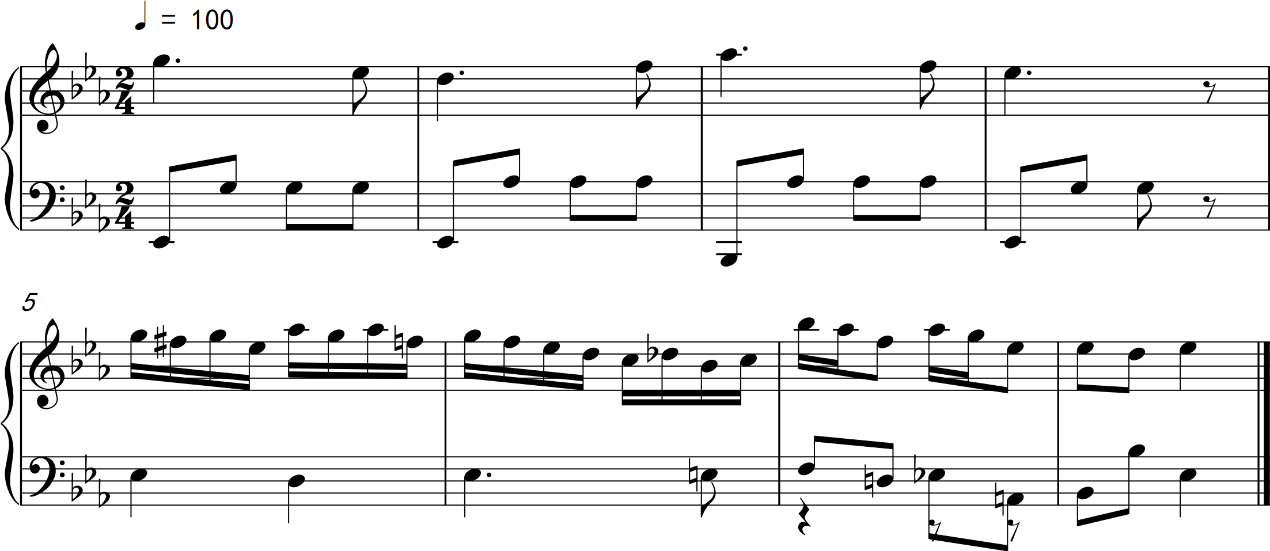
31
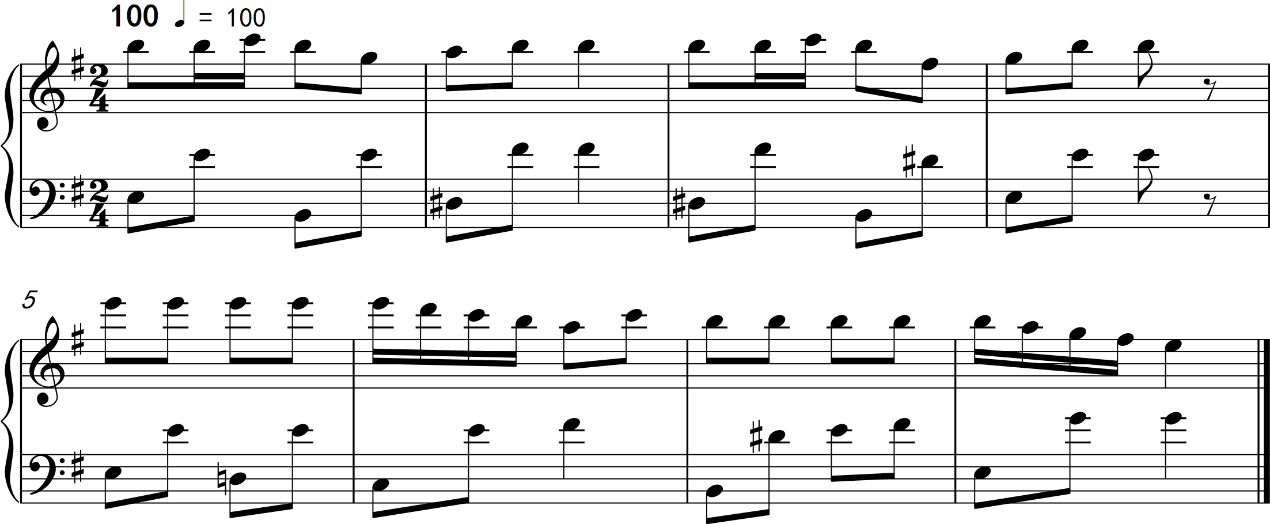
32
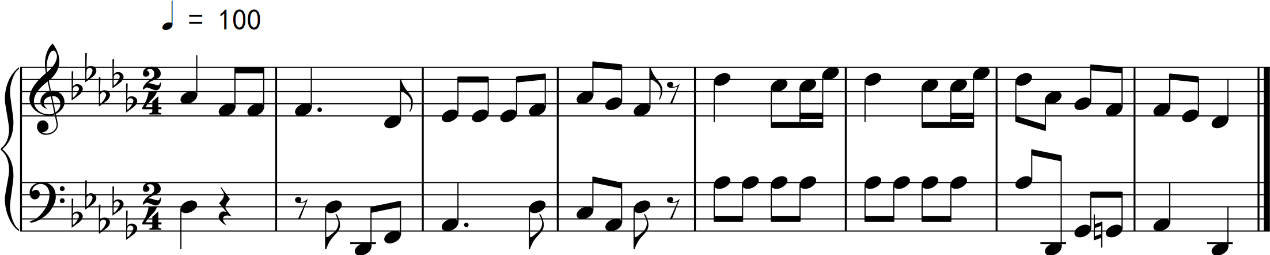
33
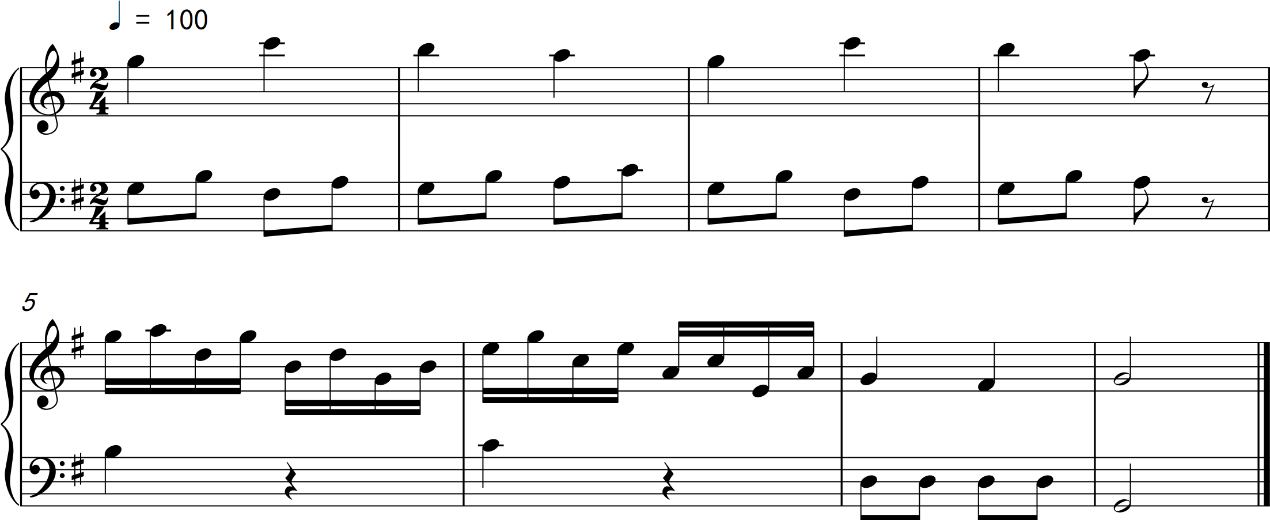
34
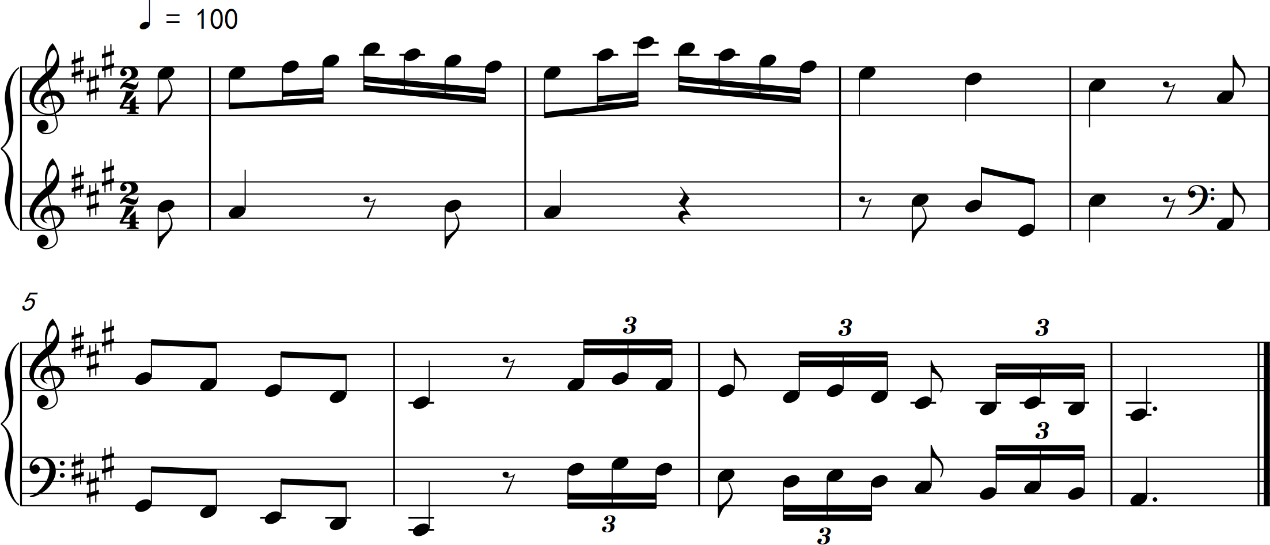
35
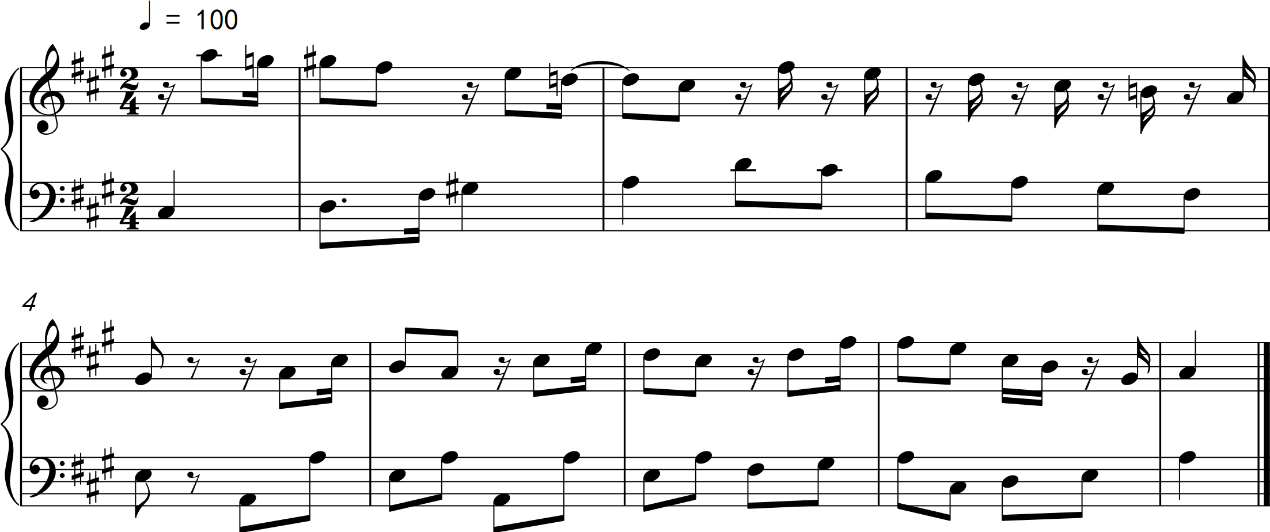
36
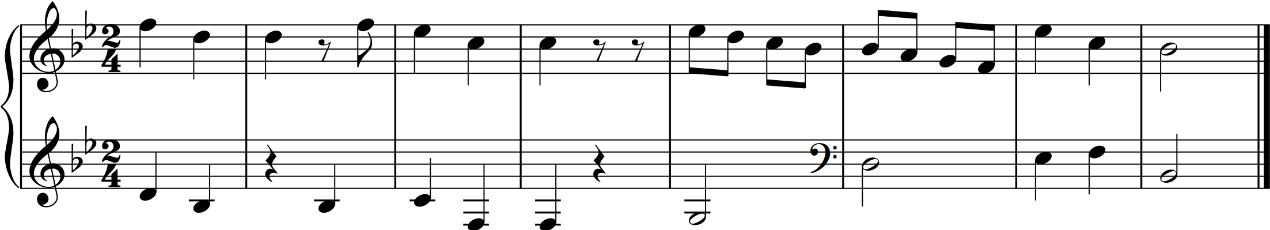
37
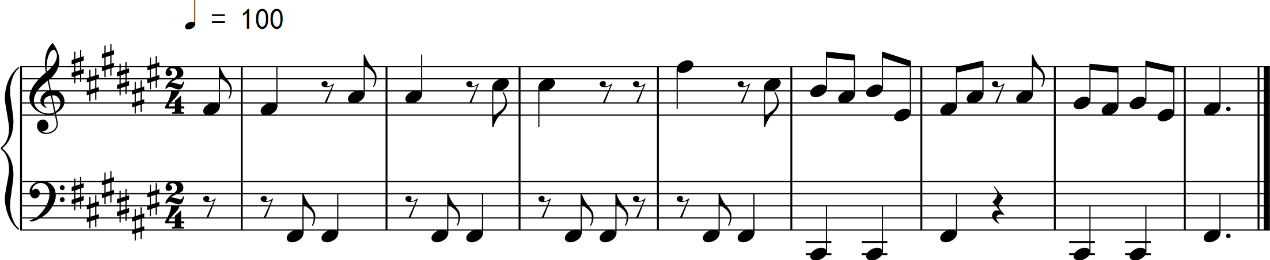
38
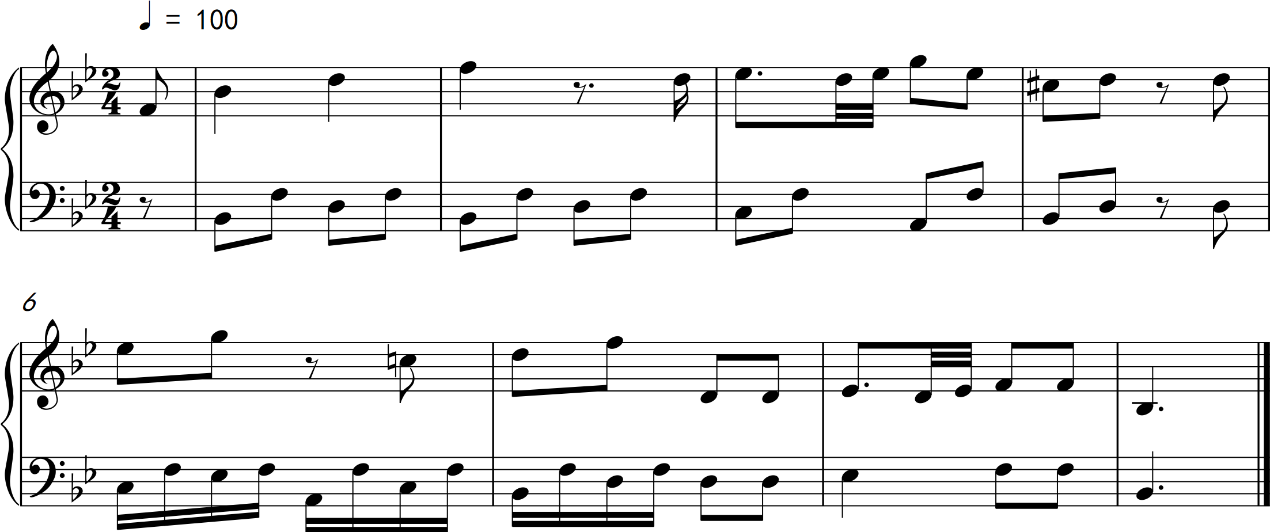
39
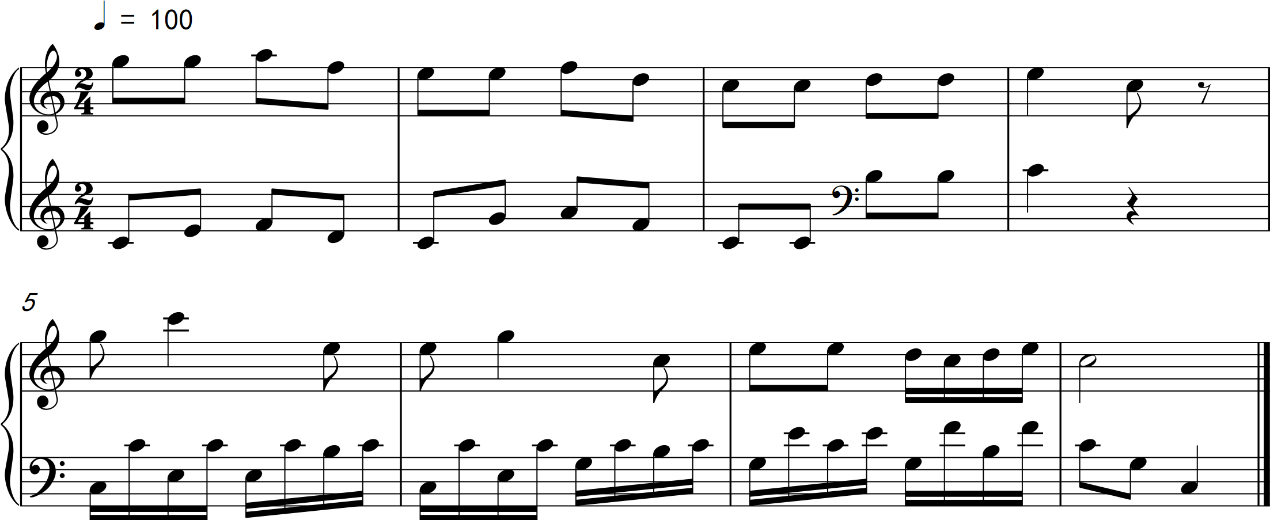
40
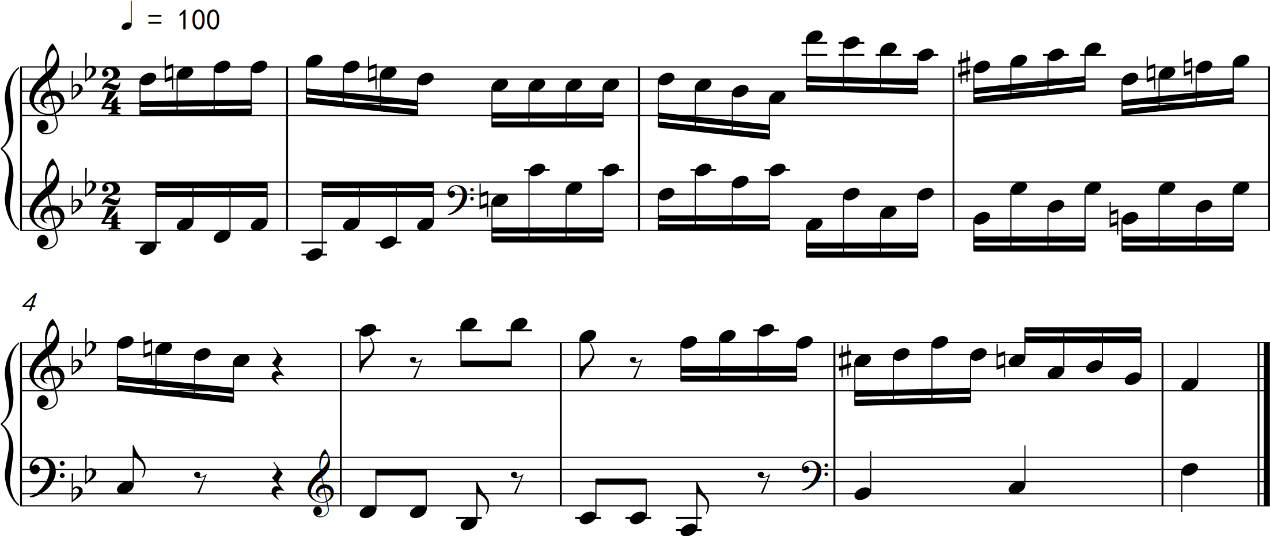
41
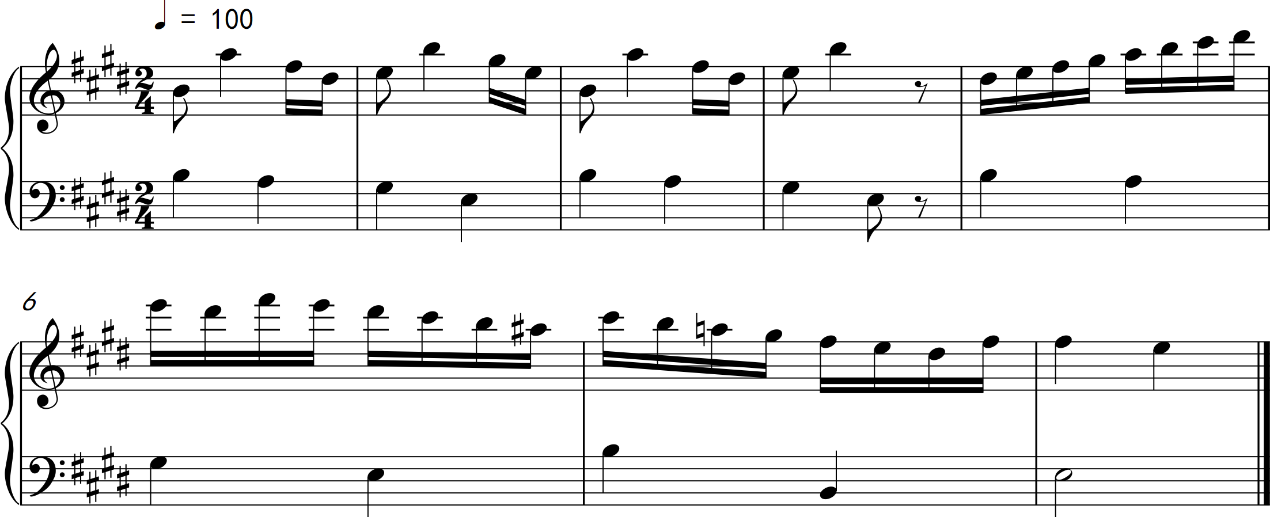
42
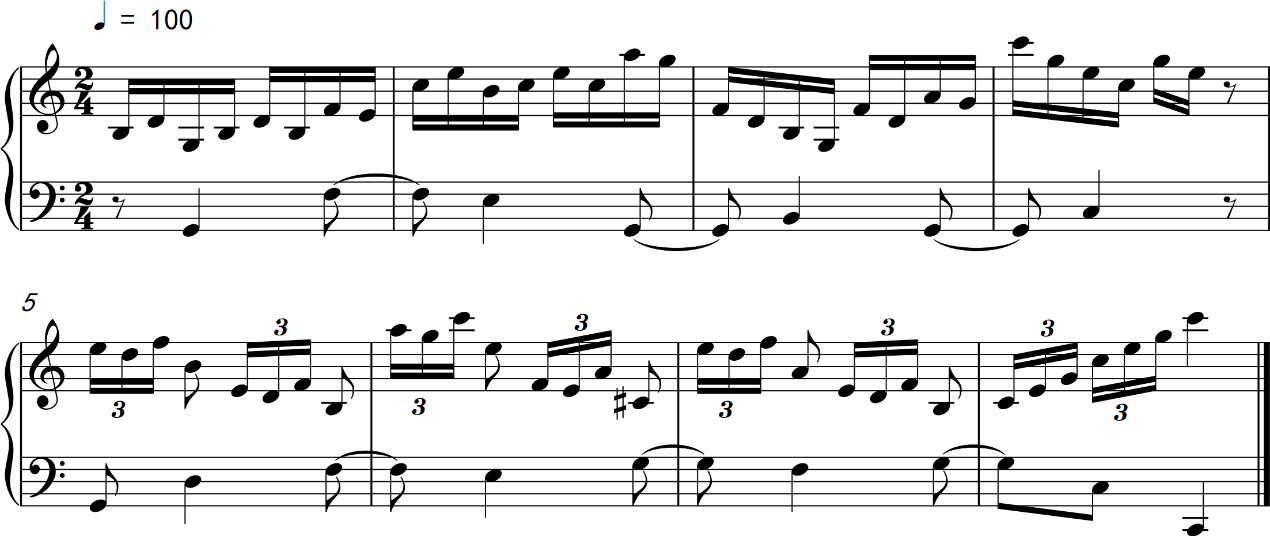
43
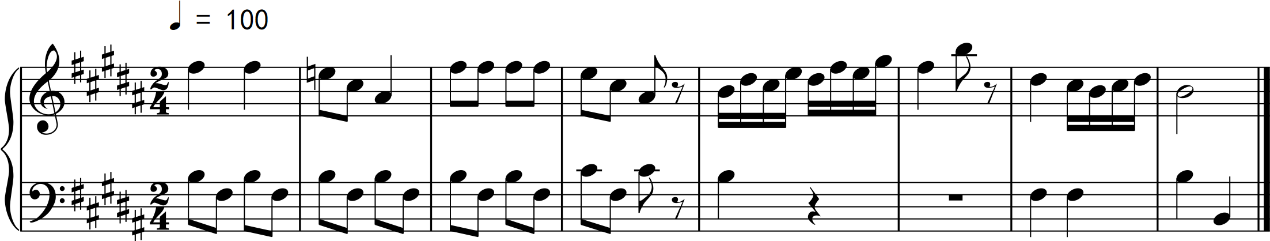
45
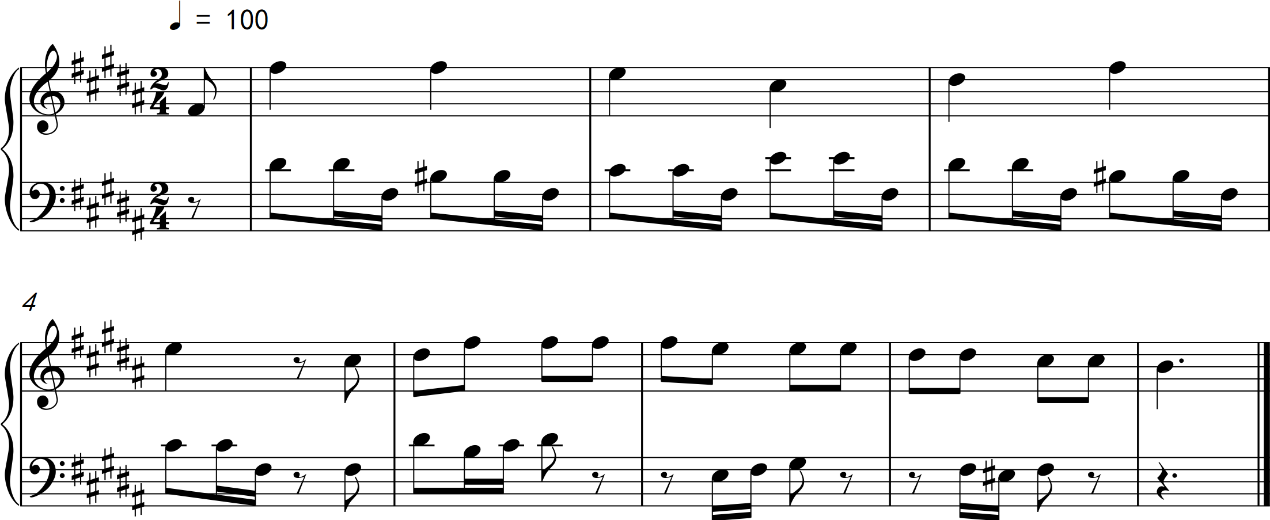
45
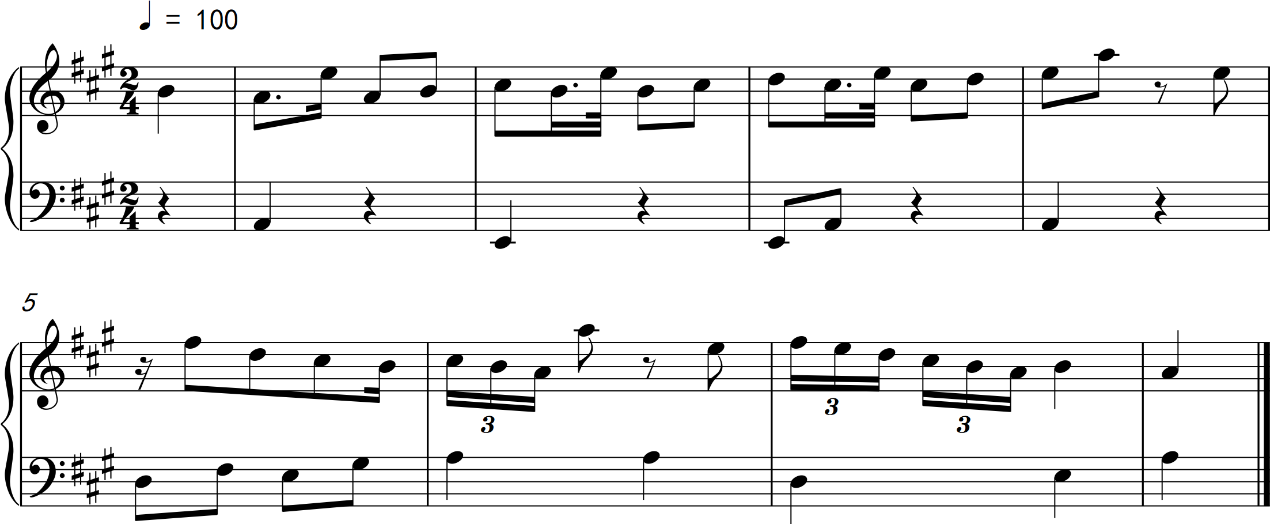
46
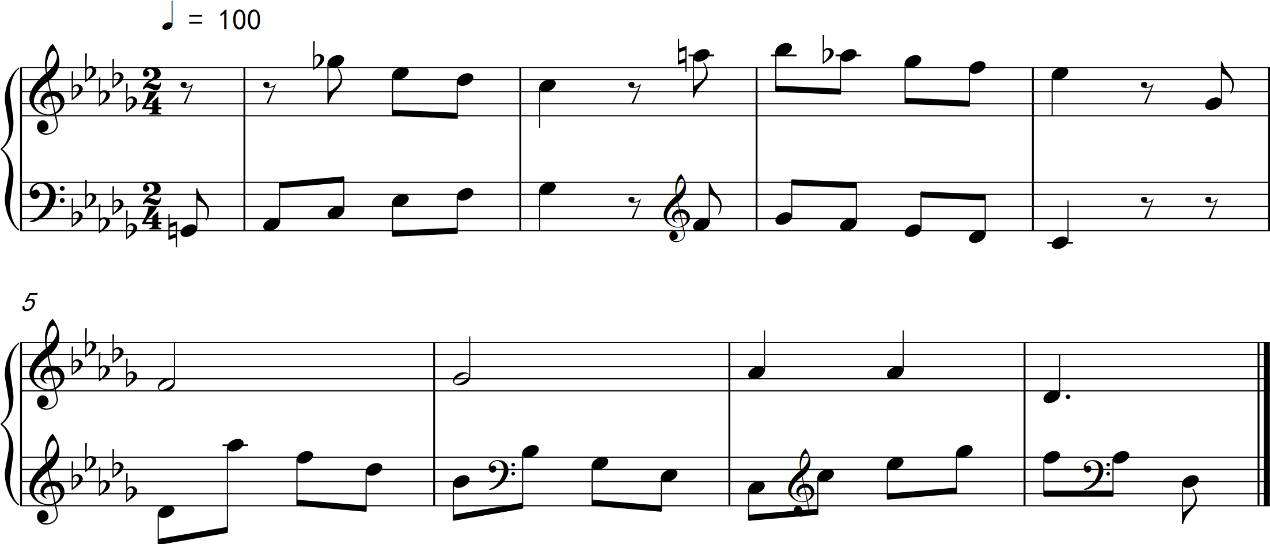
47
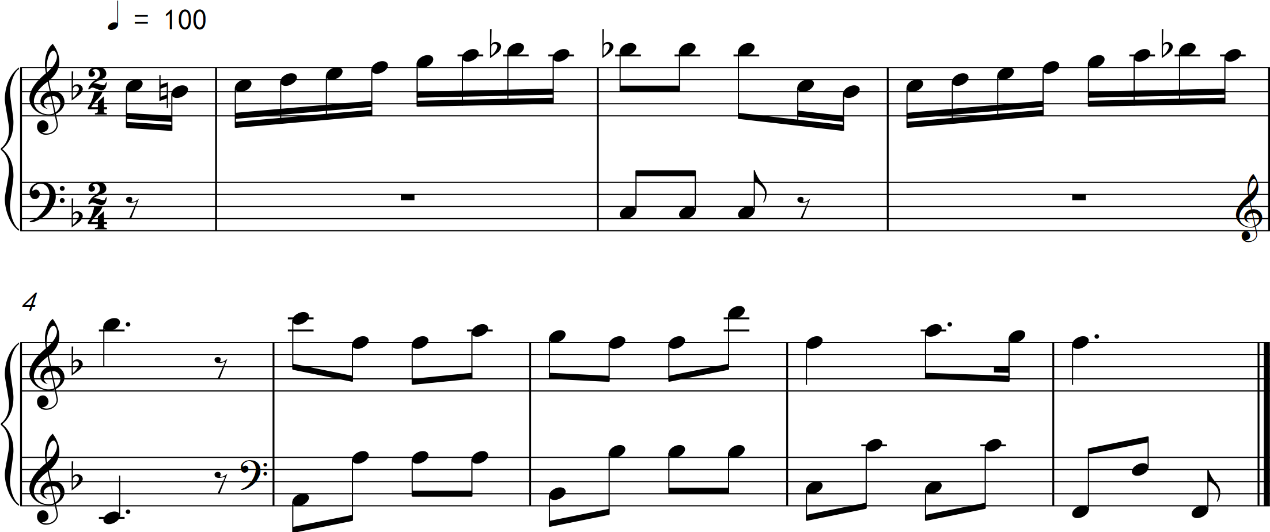
48
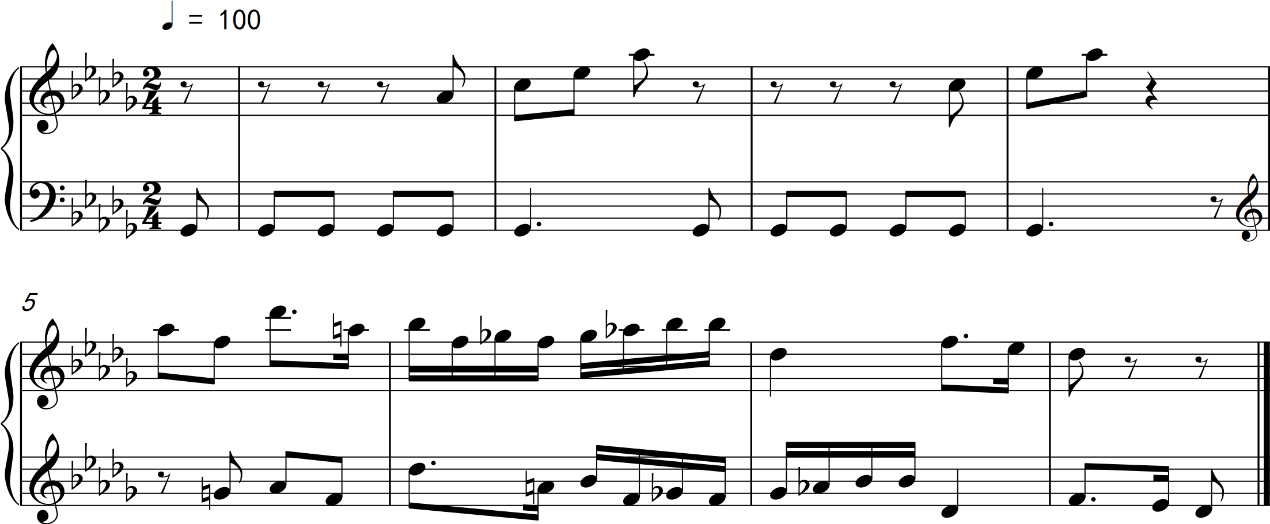
49
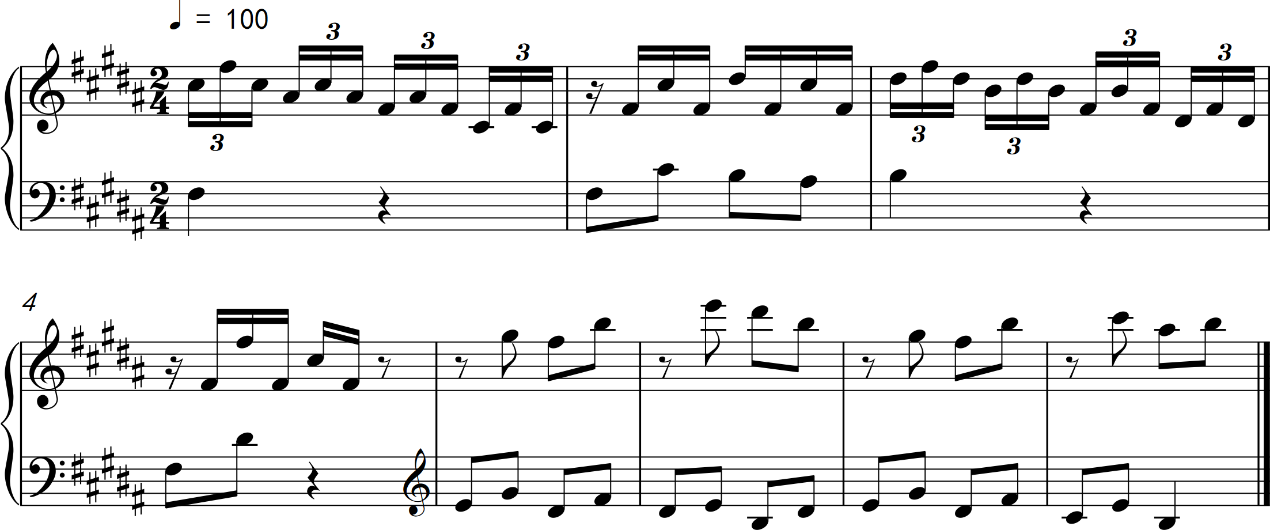
50
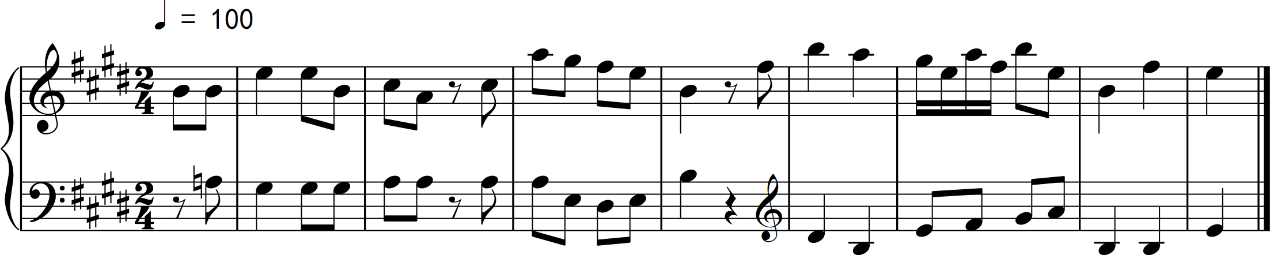
51
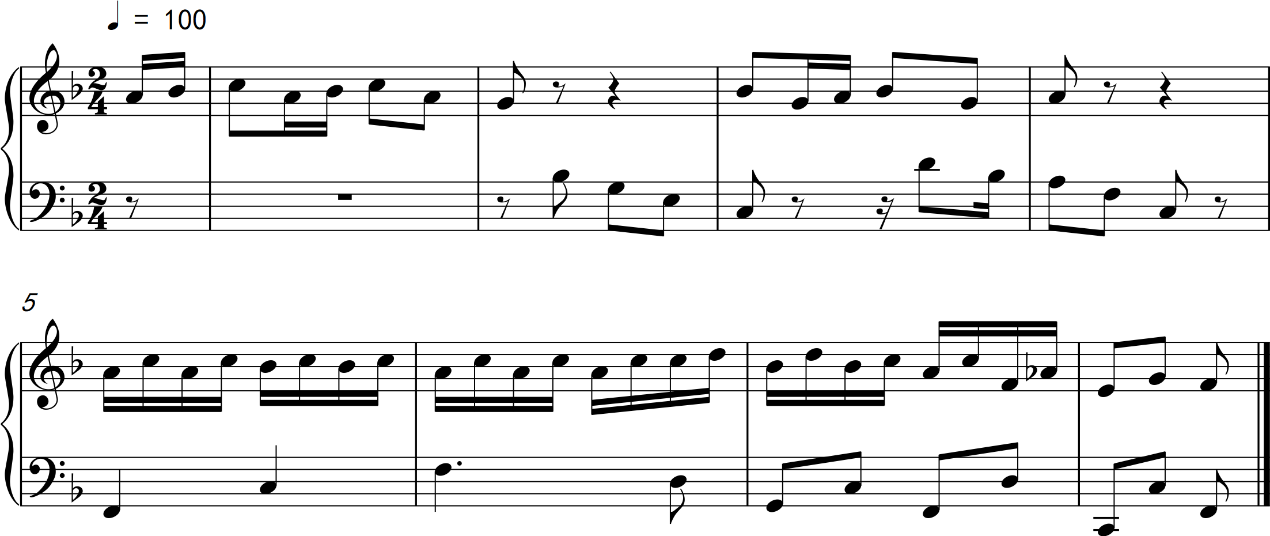
52
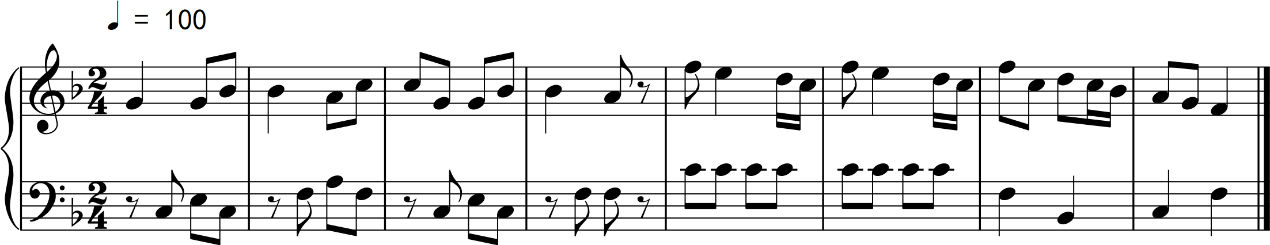
53
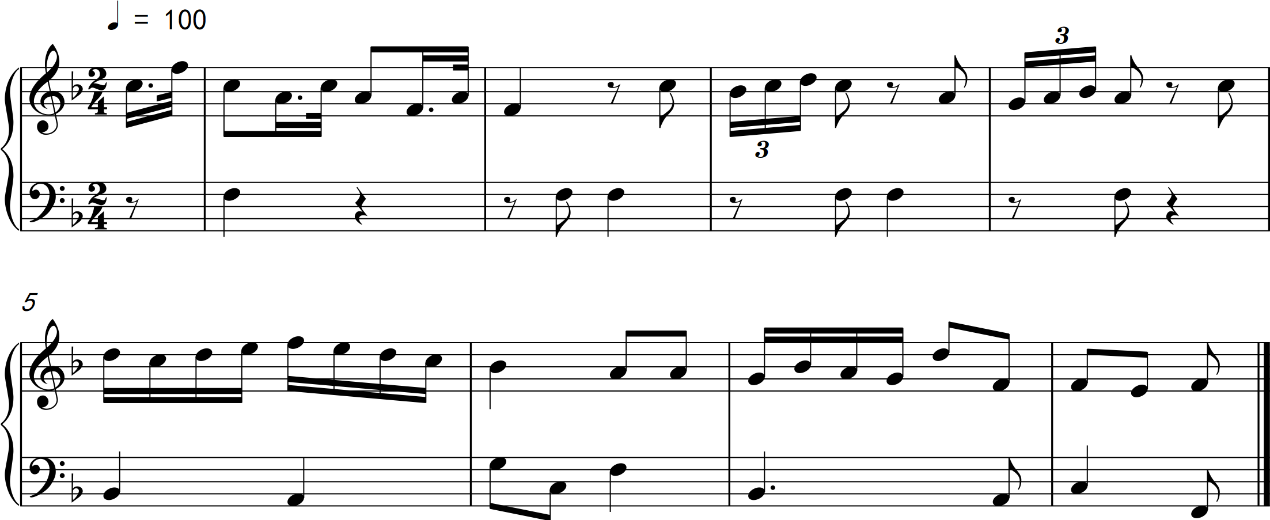
54
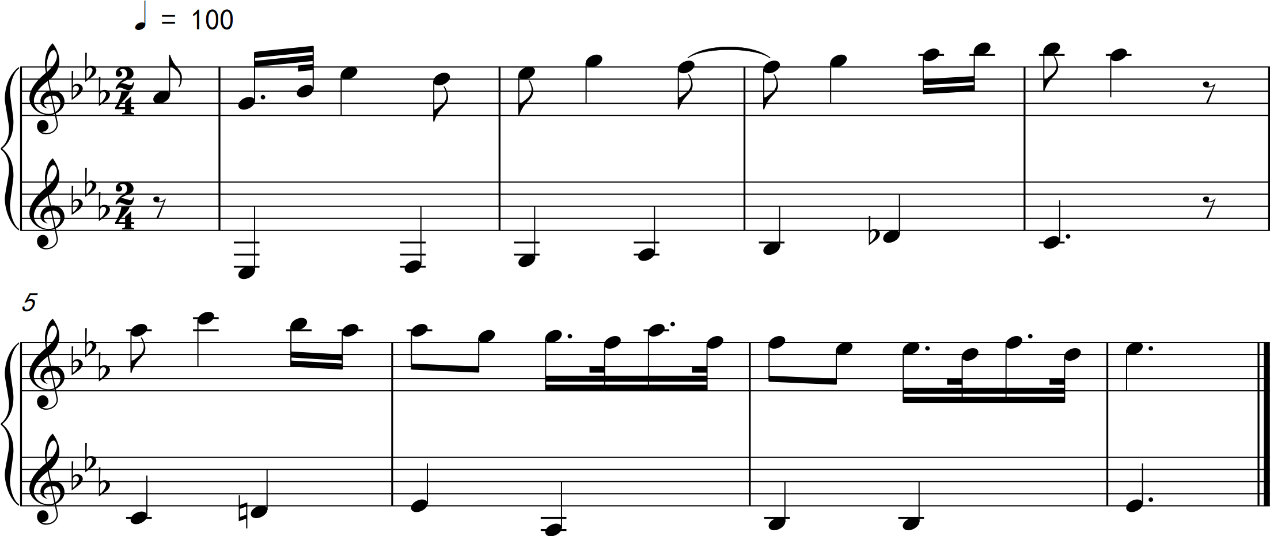
55
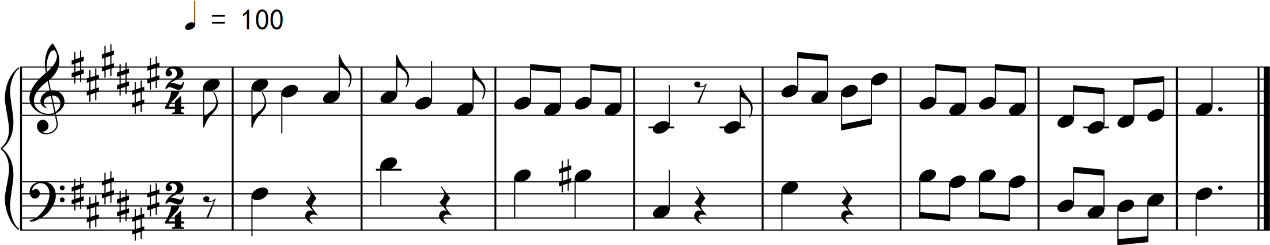
56
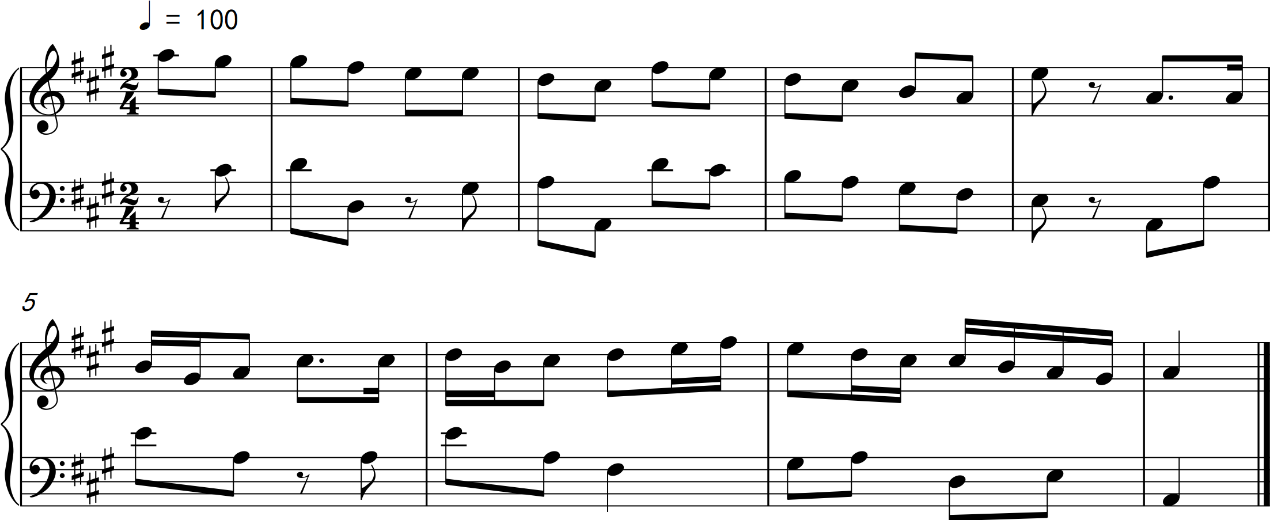
57
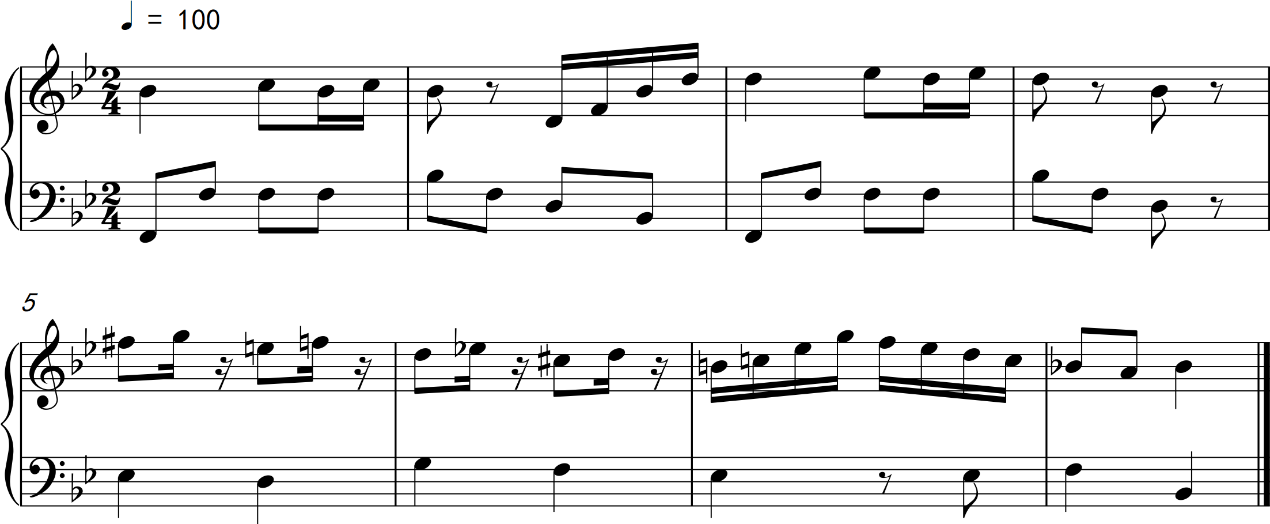
58
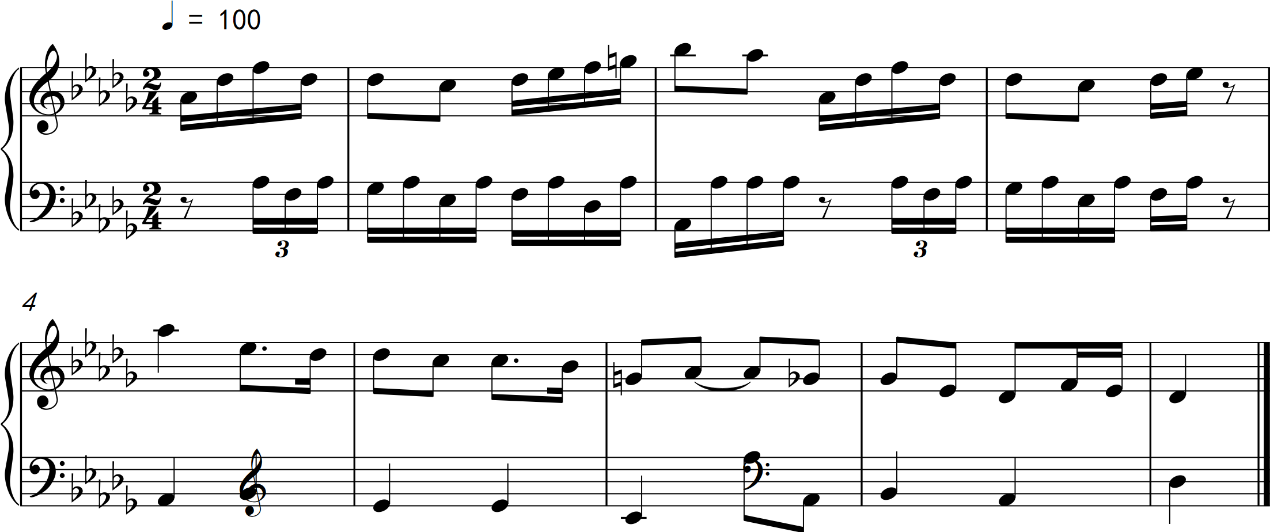
59
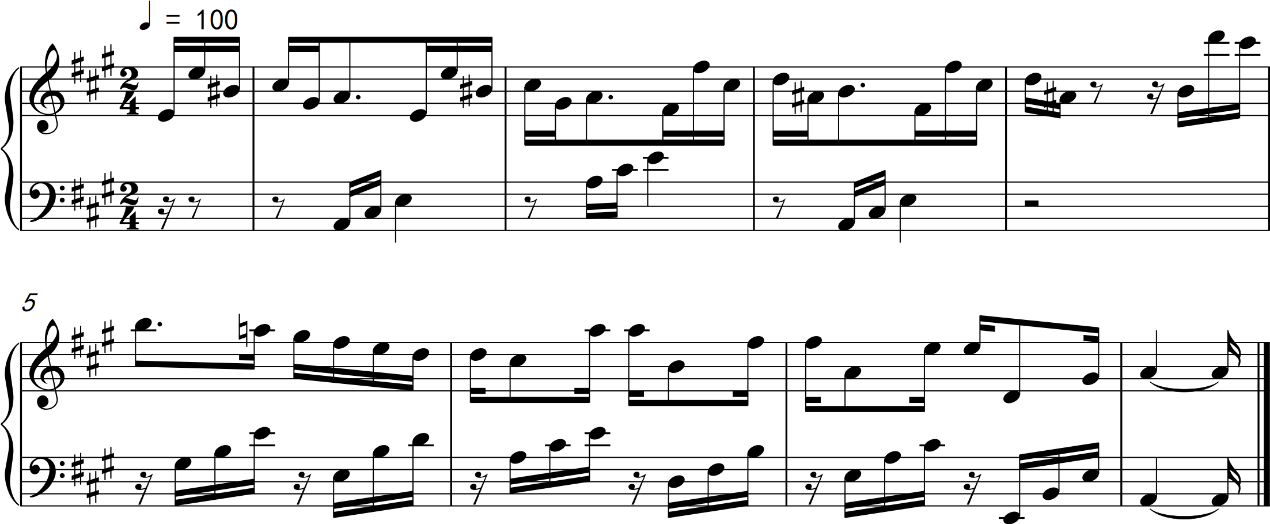
60
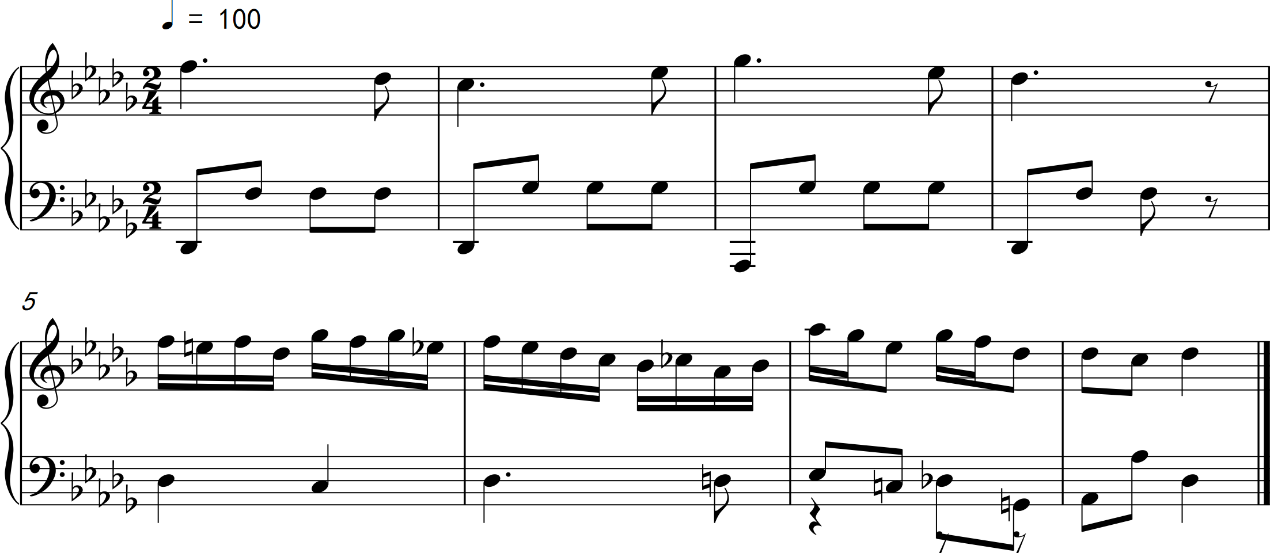

Supplement: Supplementary file 1 — Supplementary Information 1. [file 41598_2022_11949_MOESM1_ESM.docx]

1
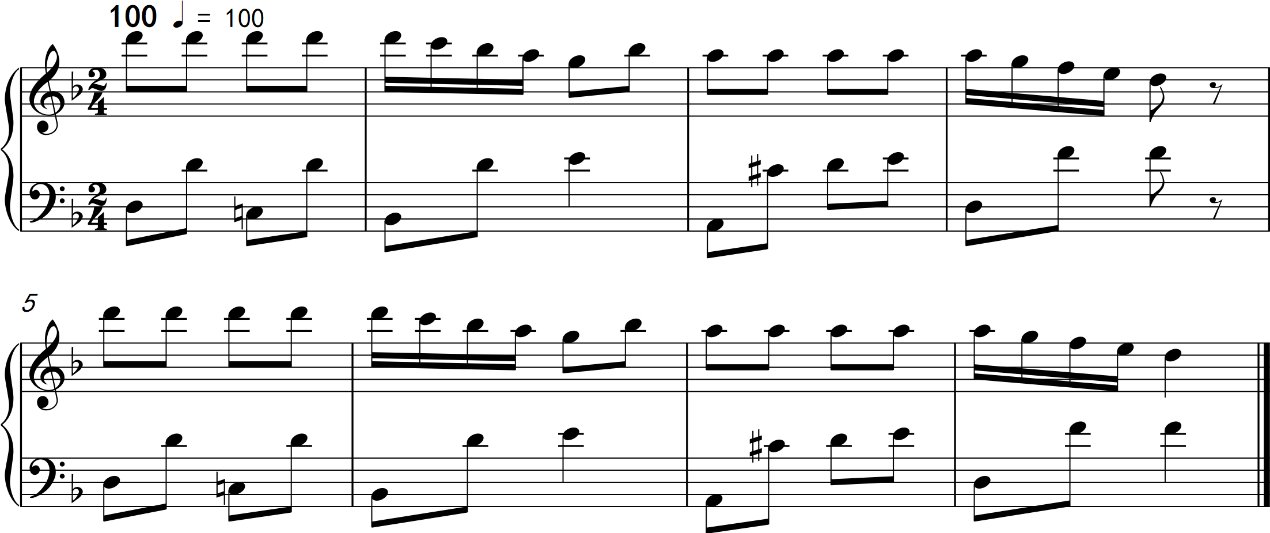
2
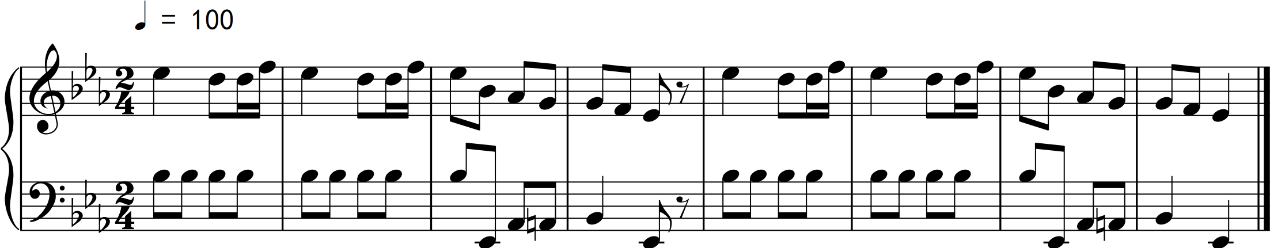
3
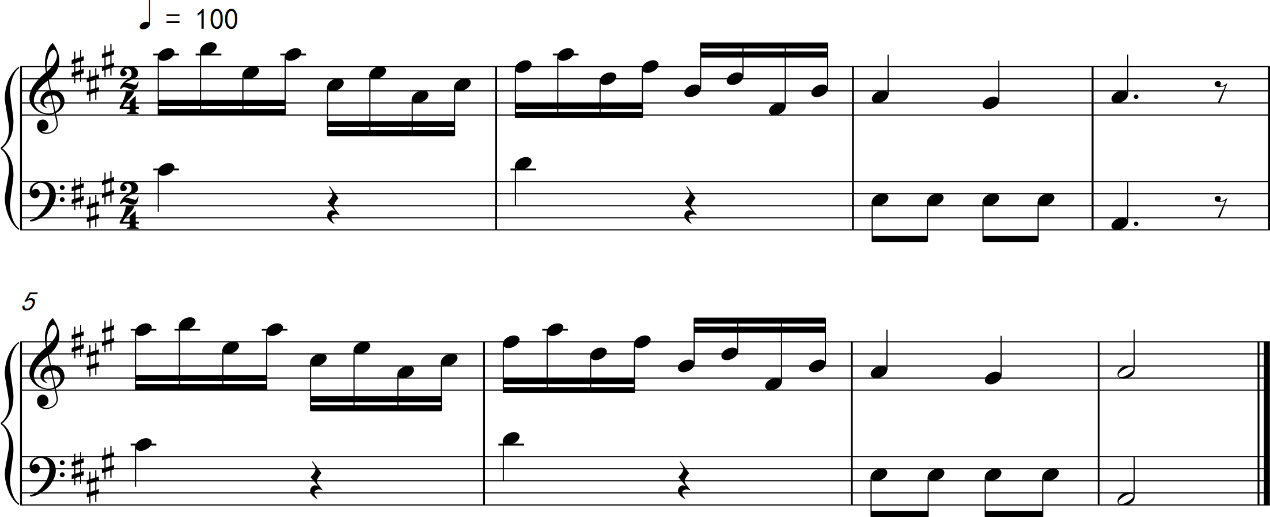
4
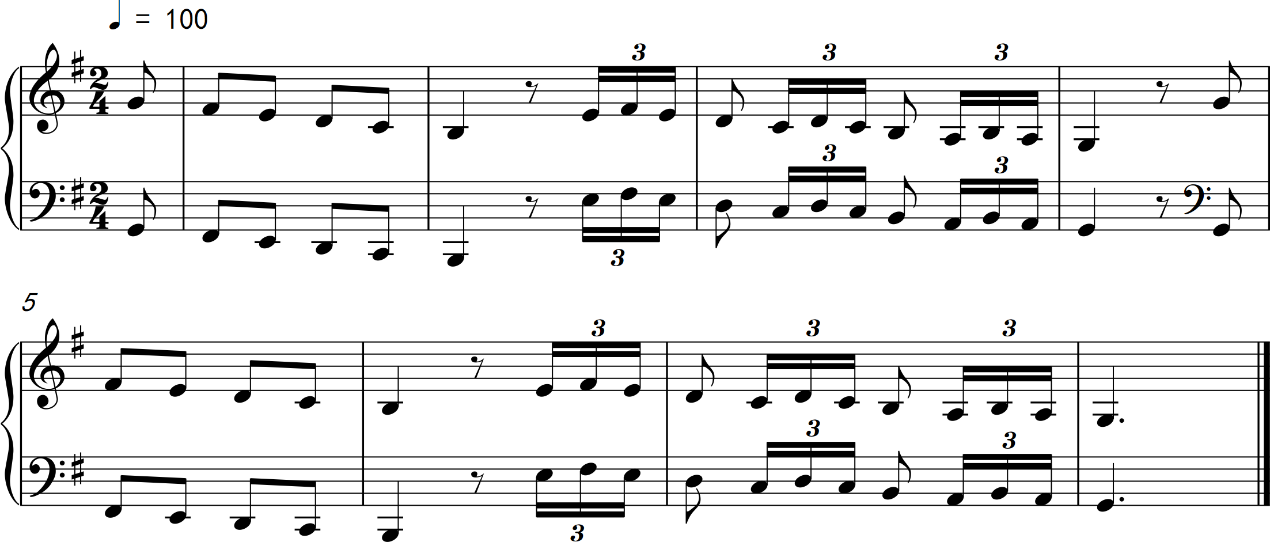
5
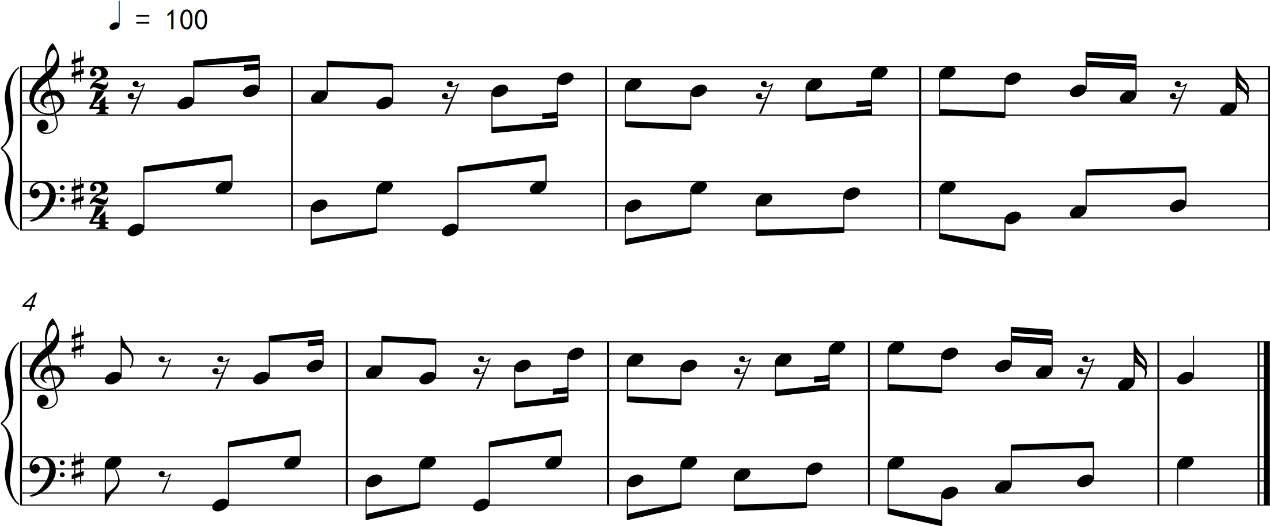
6
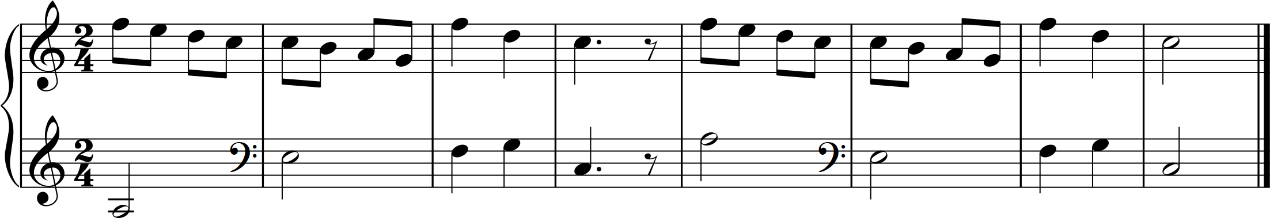
7
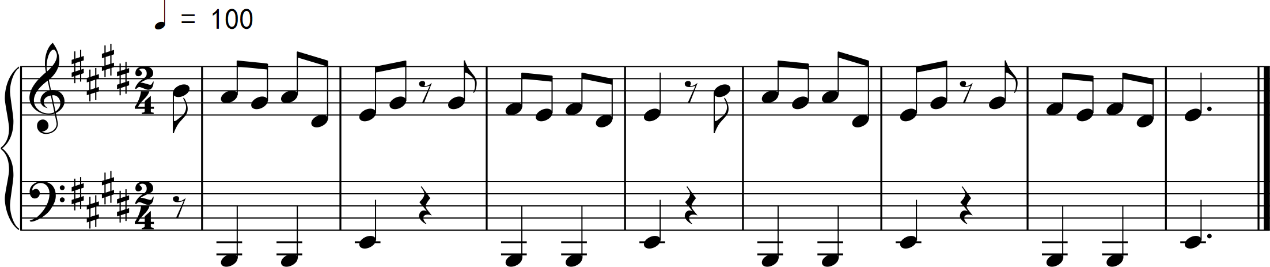
8
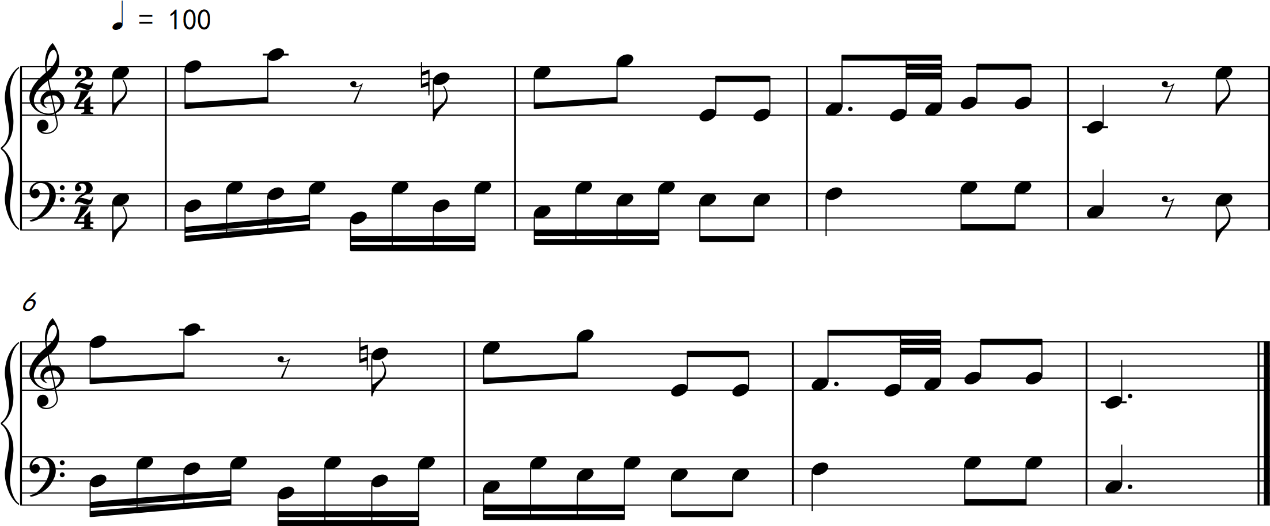
9
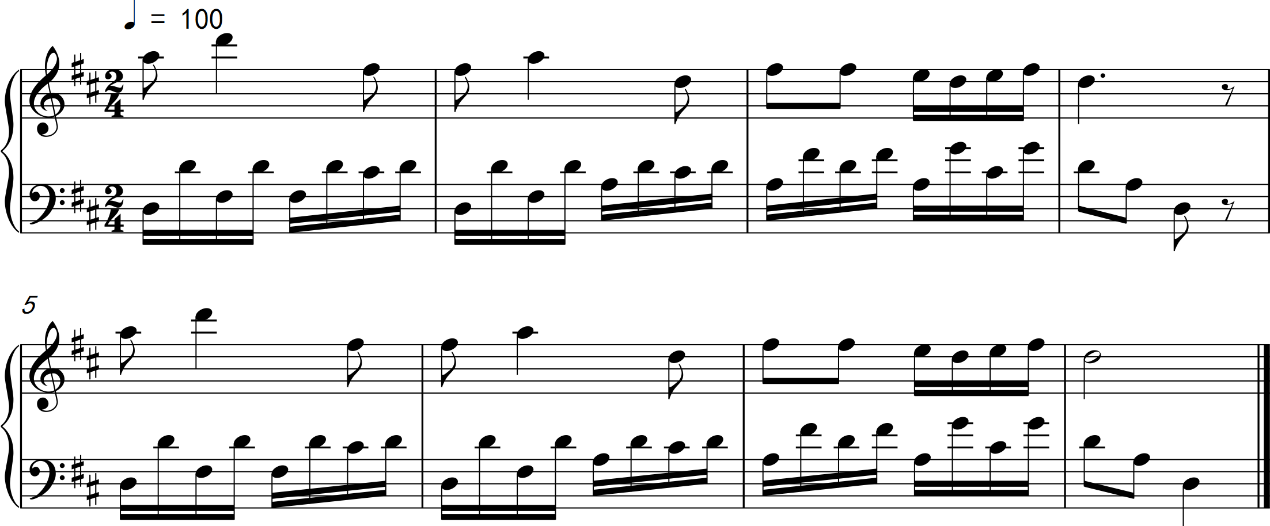
10
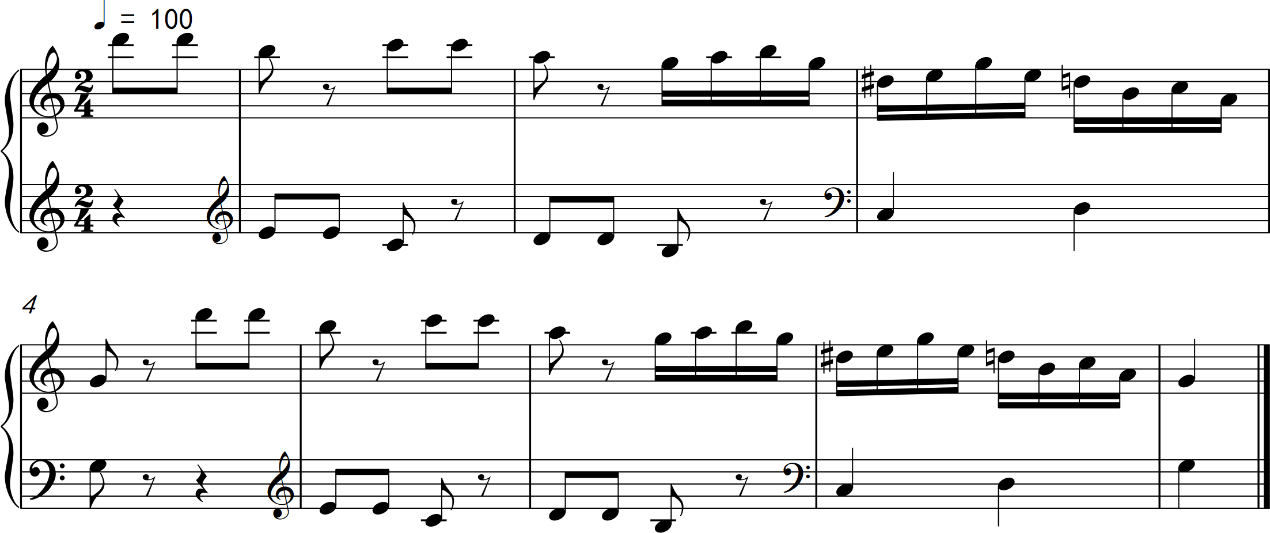
11
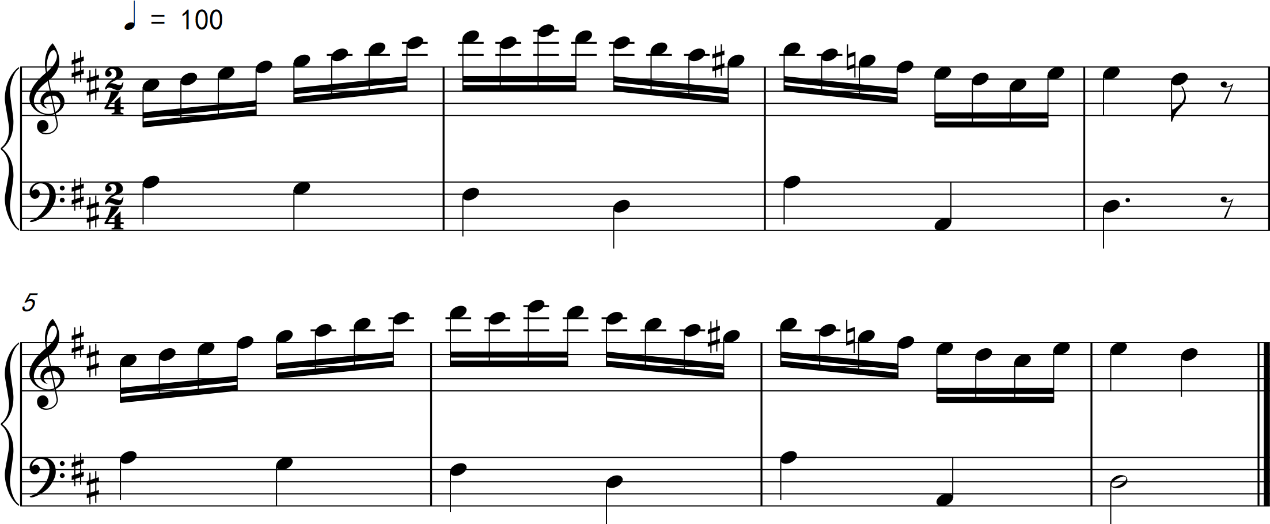
12
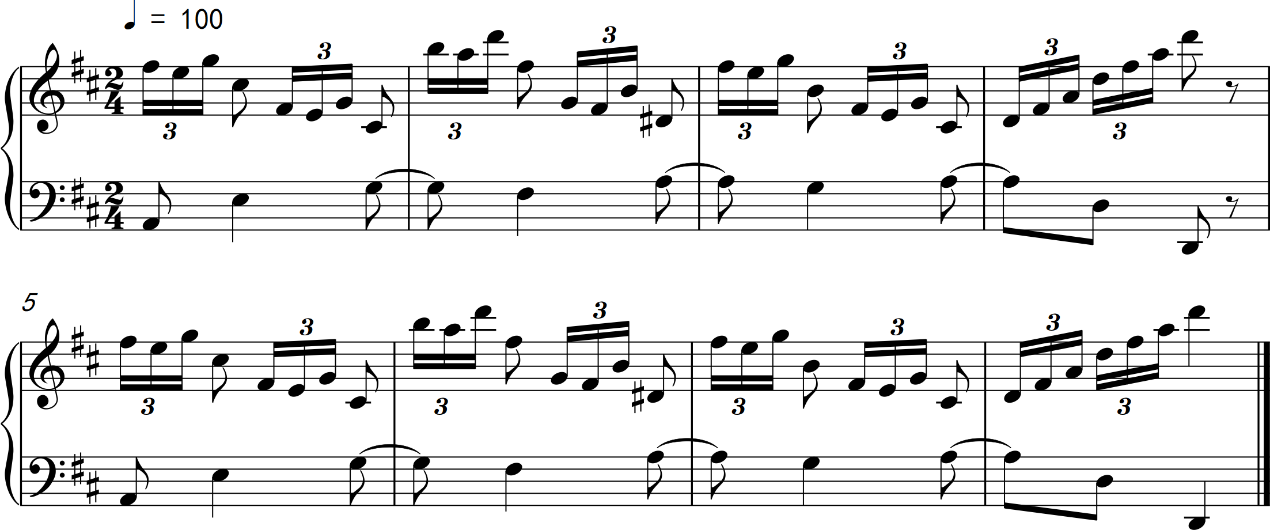
13
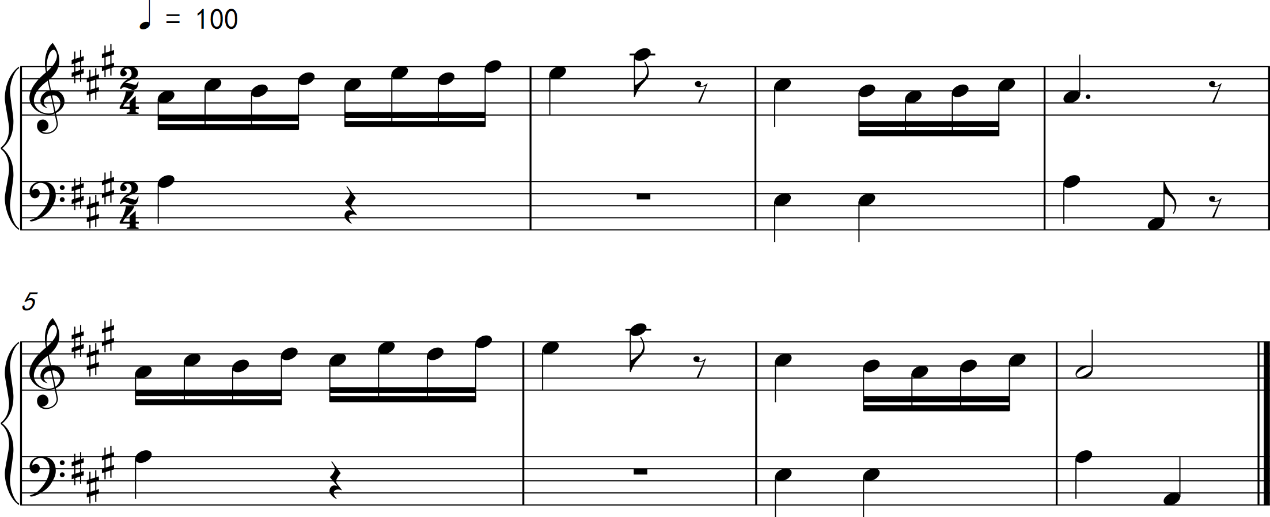
14
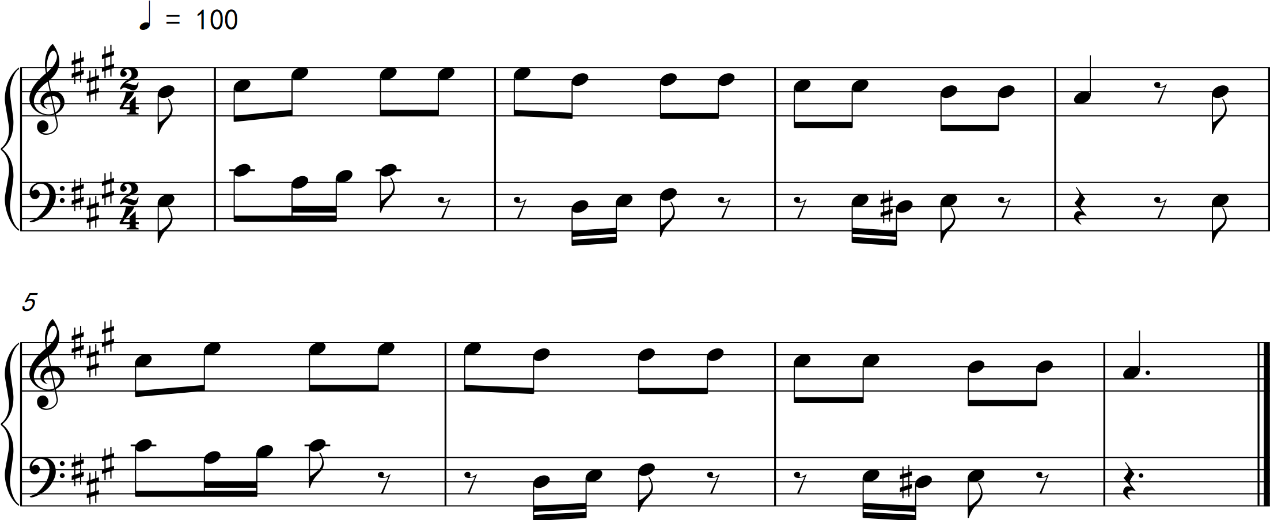
15
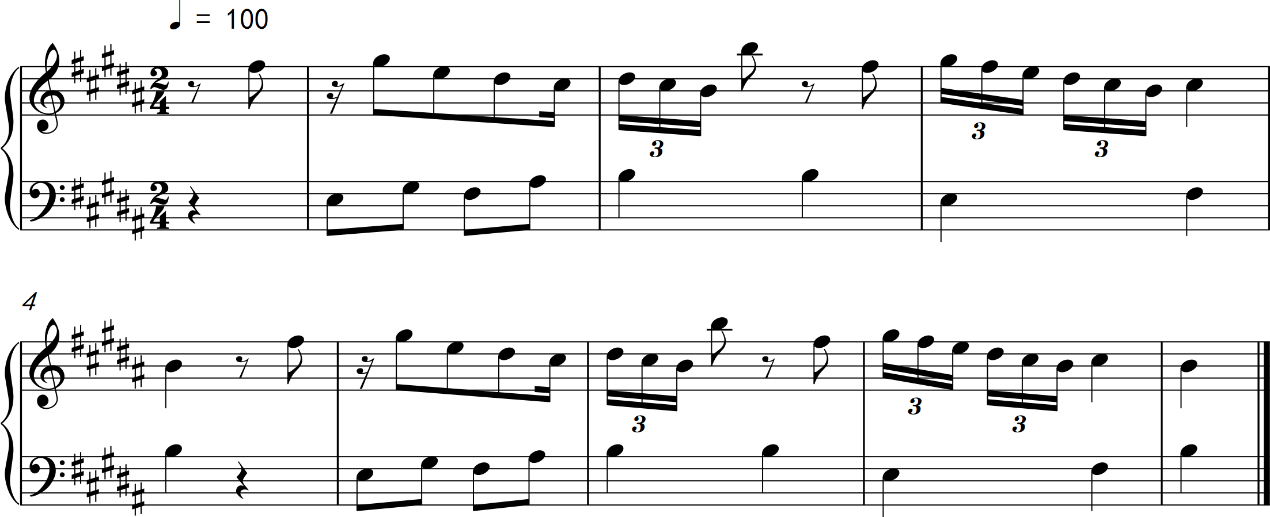
16
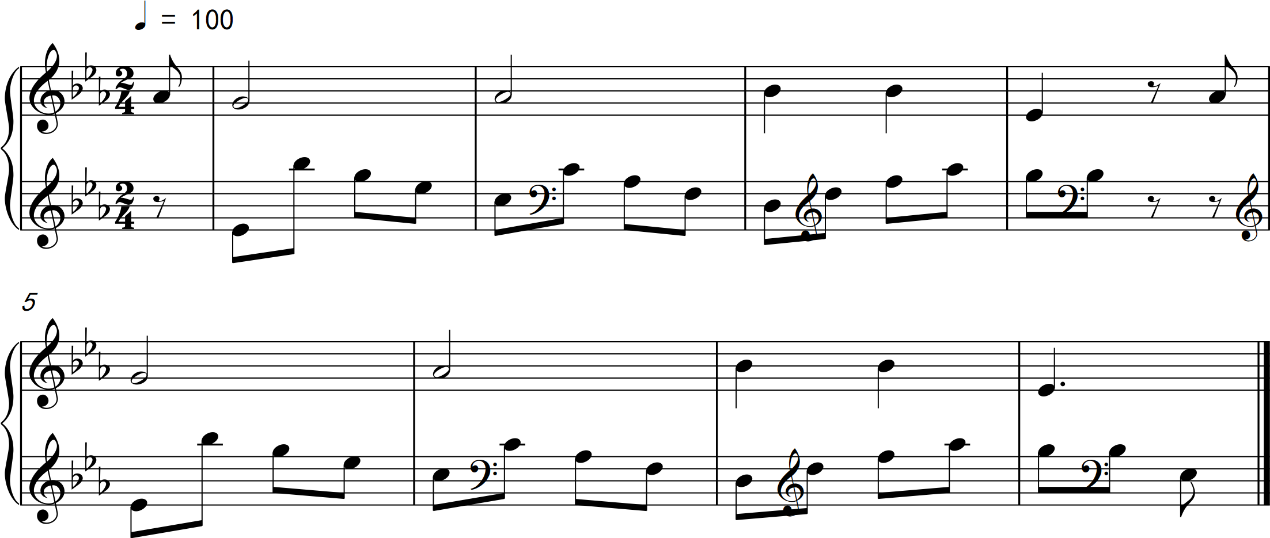
17
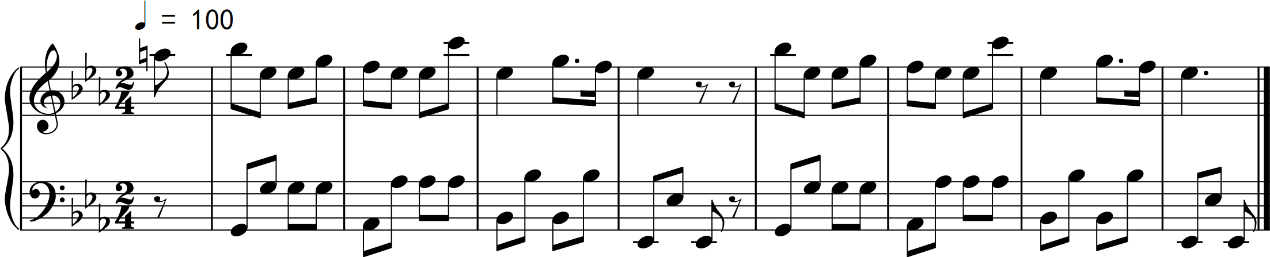
18
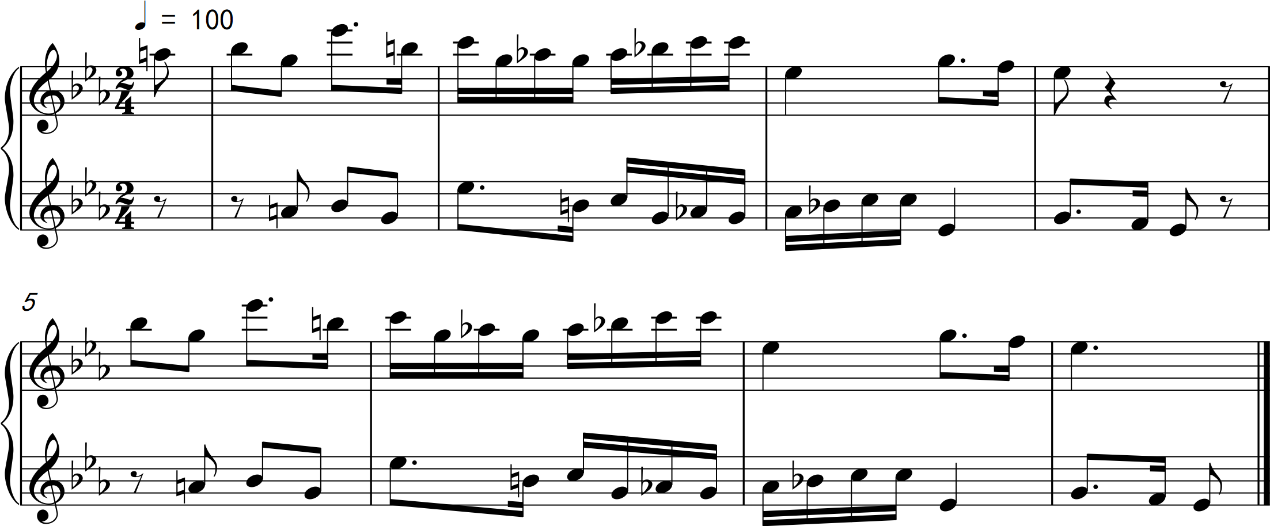
19
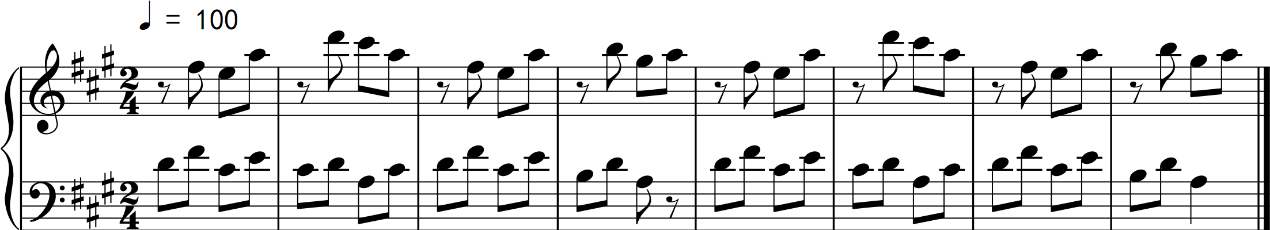
20
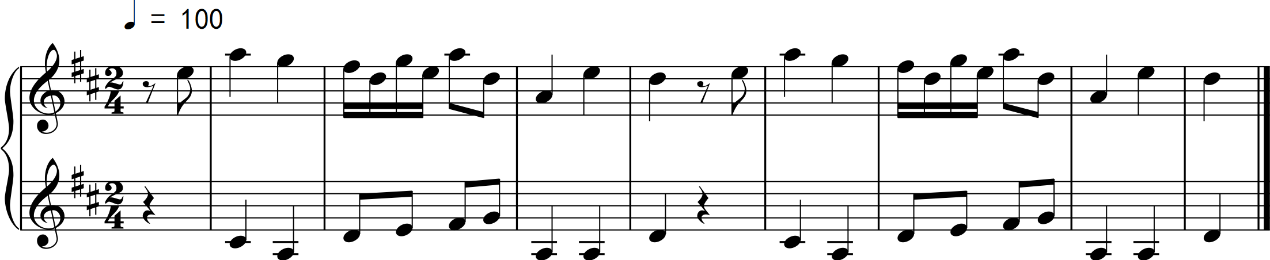
21
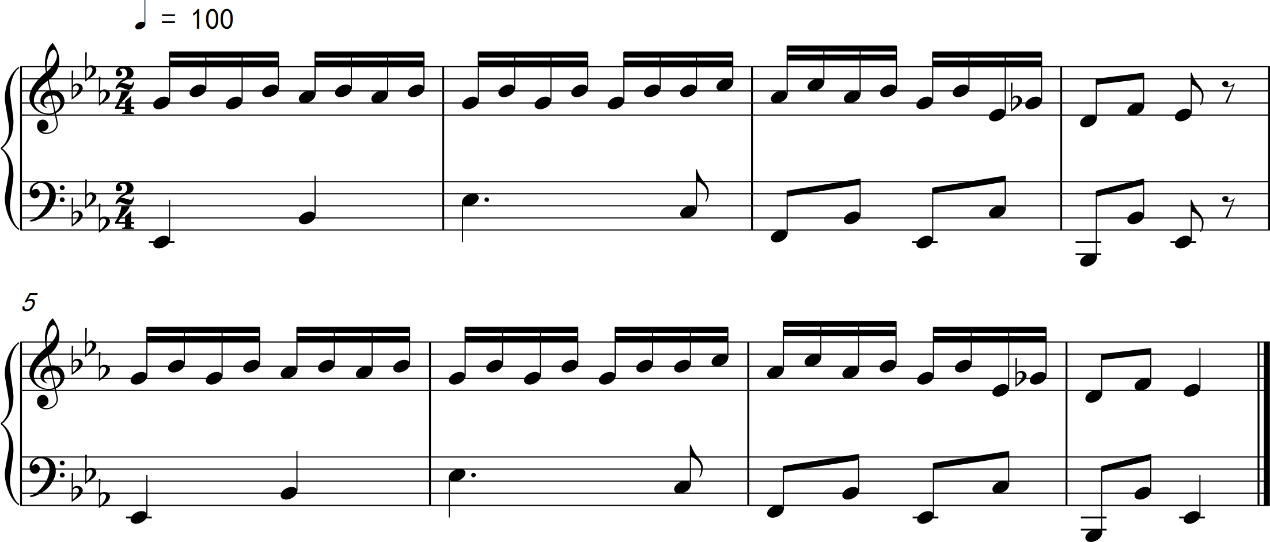
22
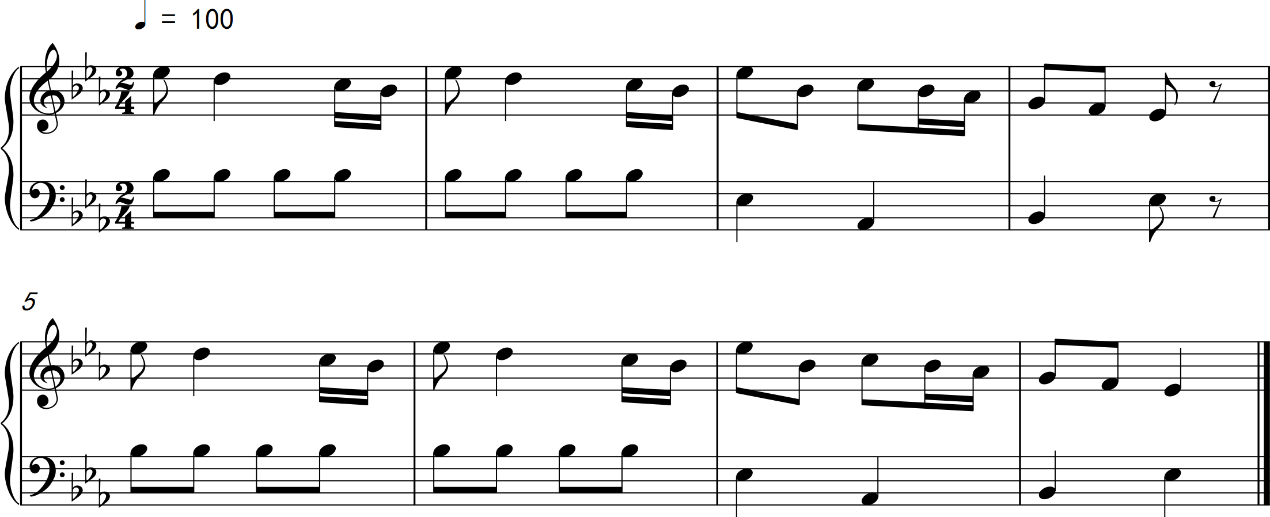
23
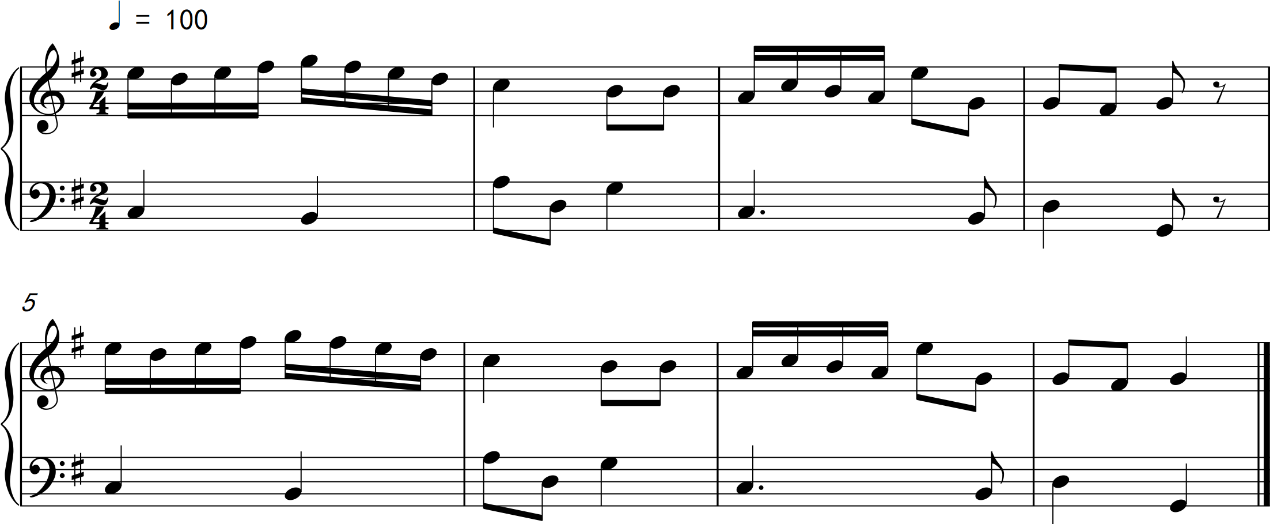
24
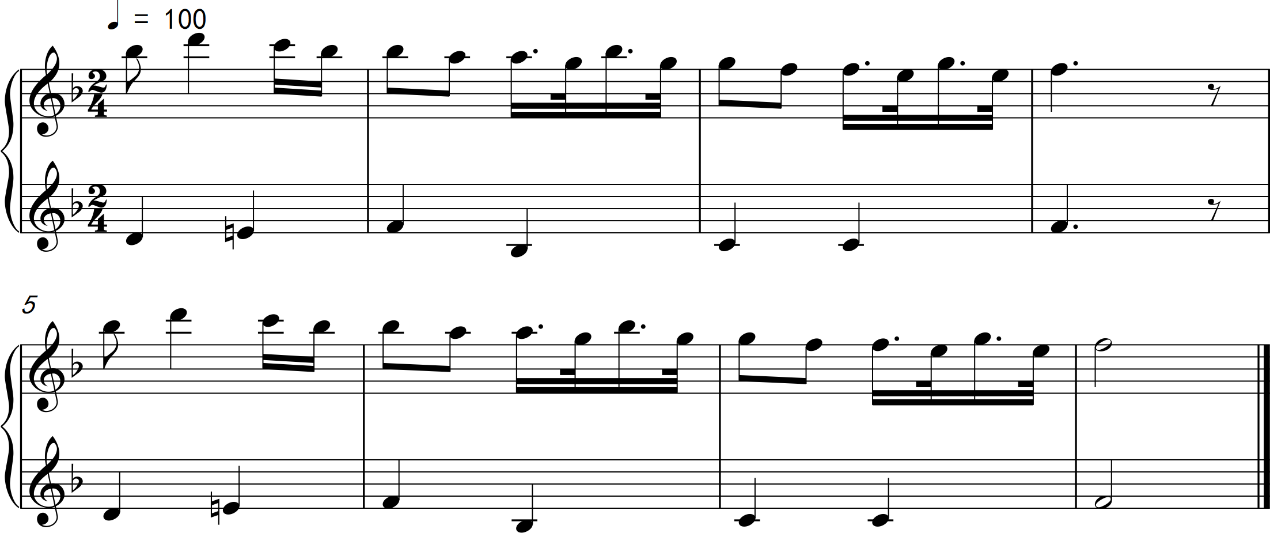
25
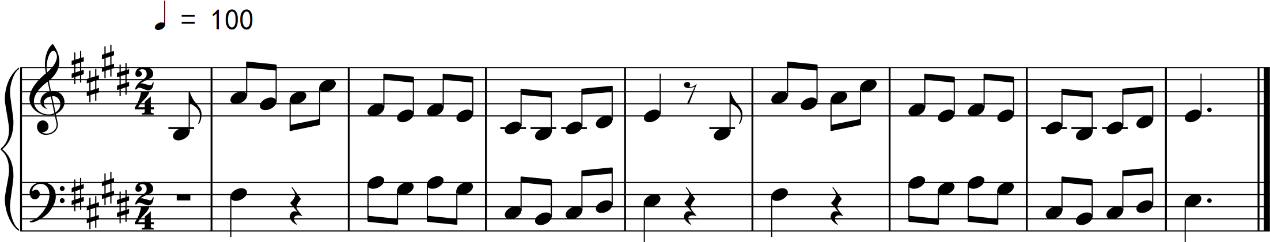
26
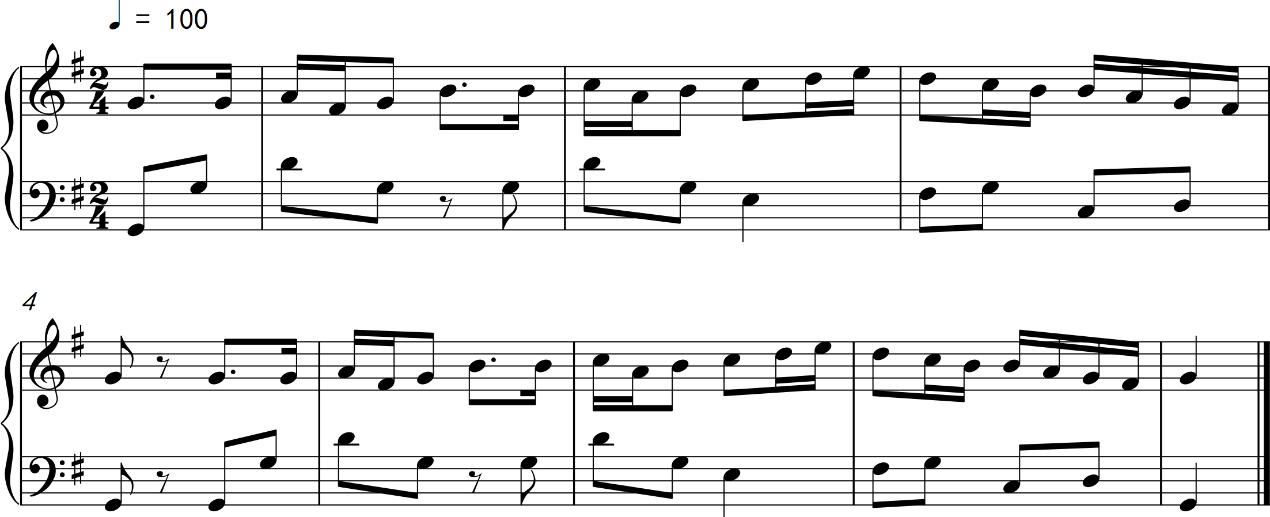
27
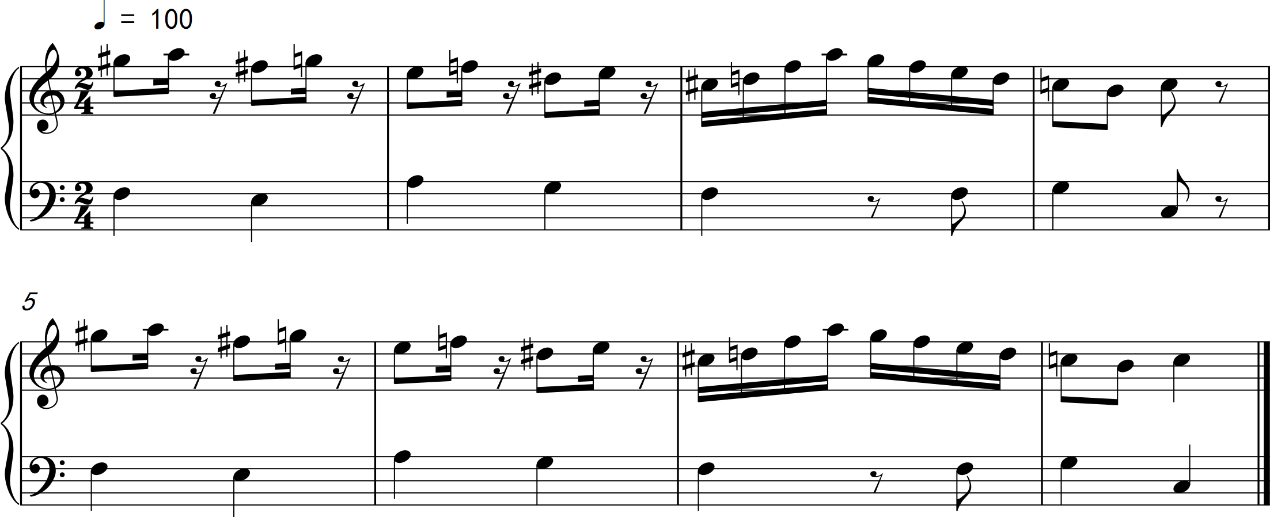
28
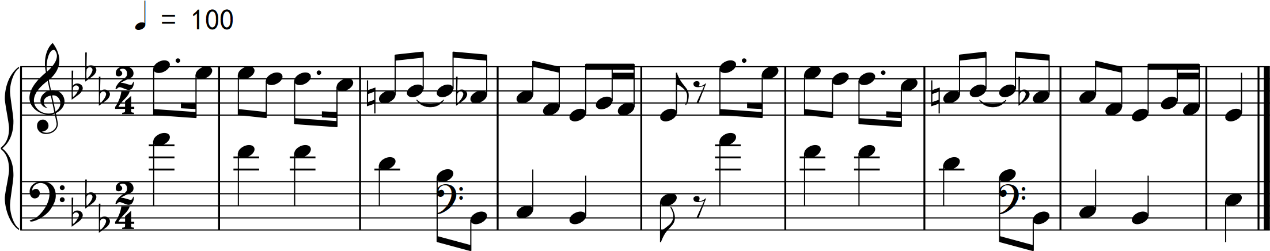
29
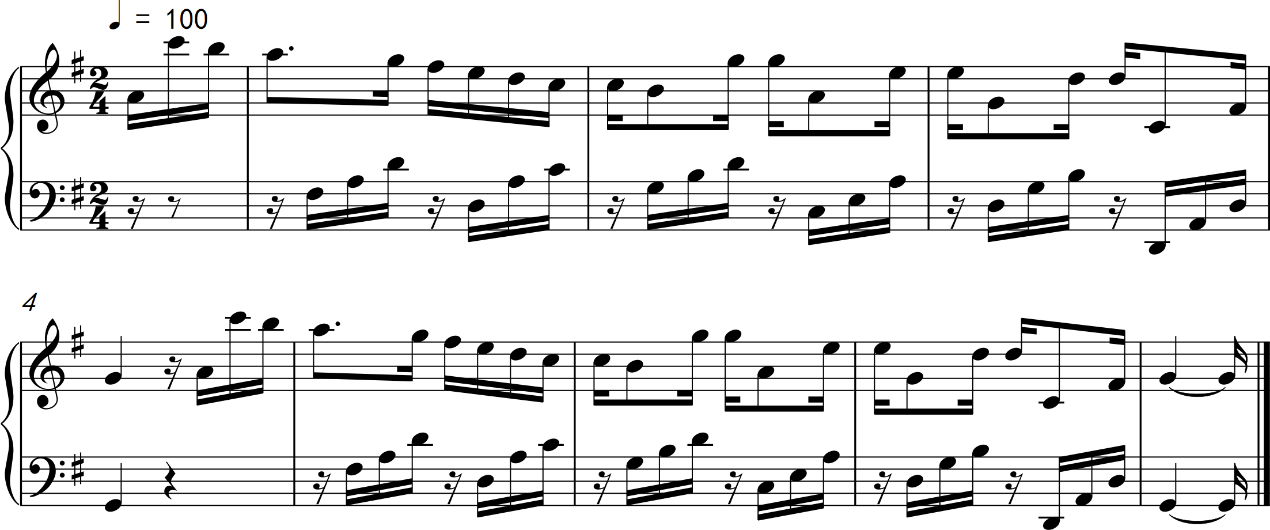
30
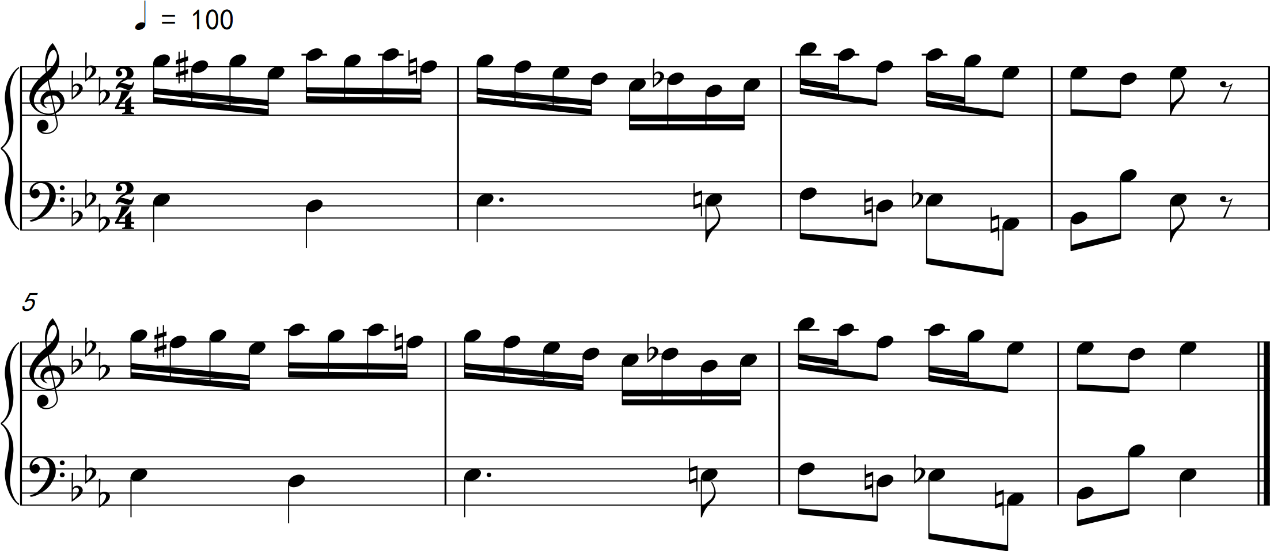
31
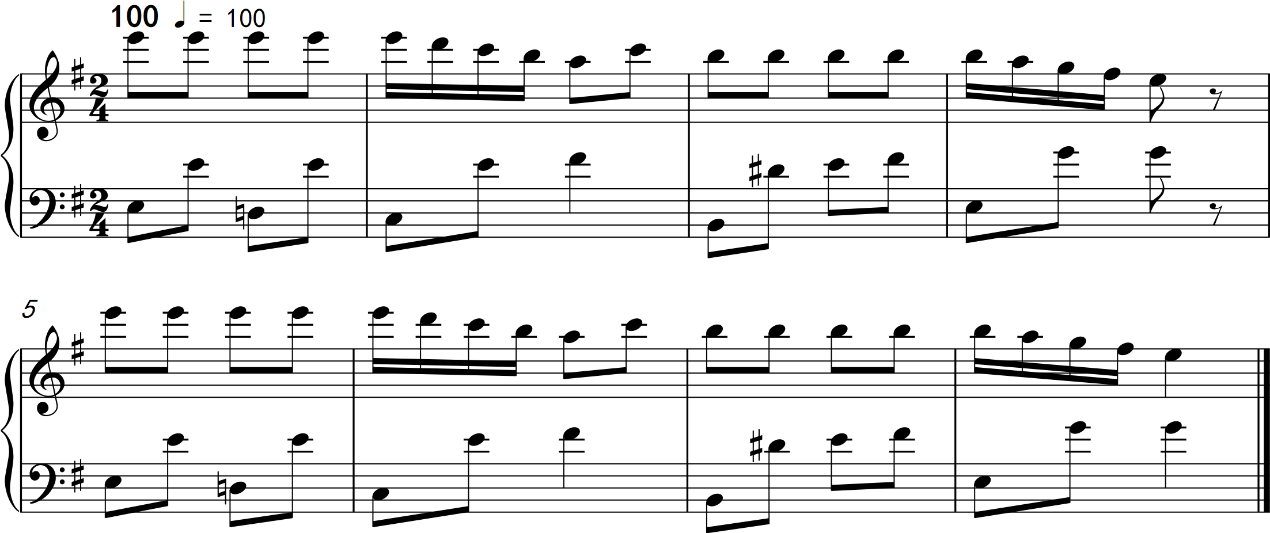
32
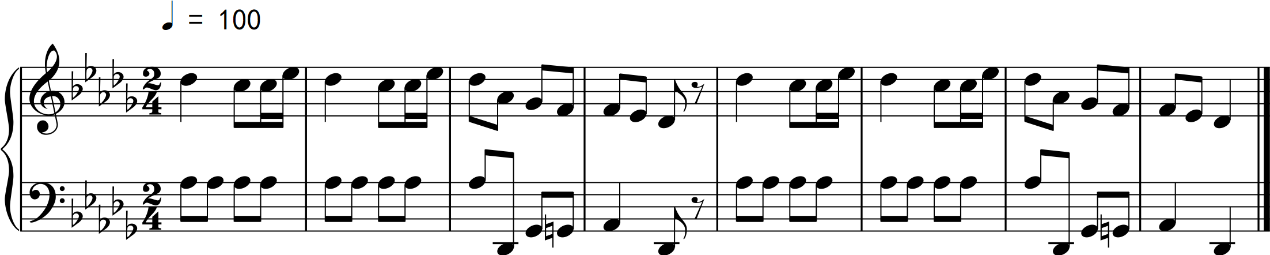
33
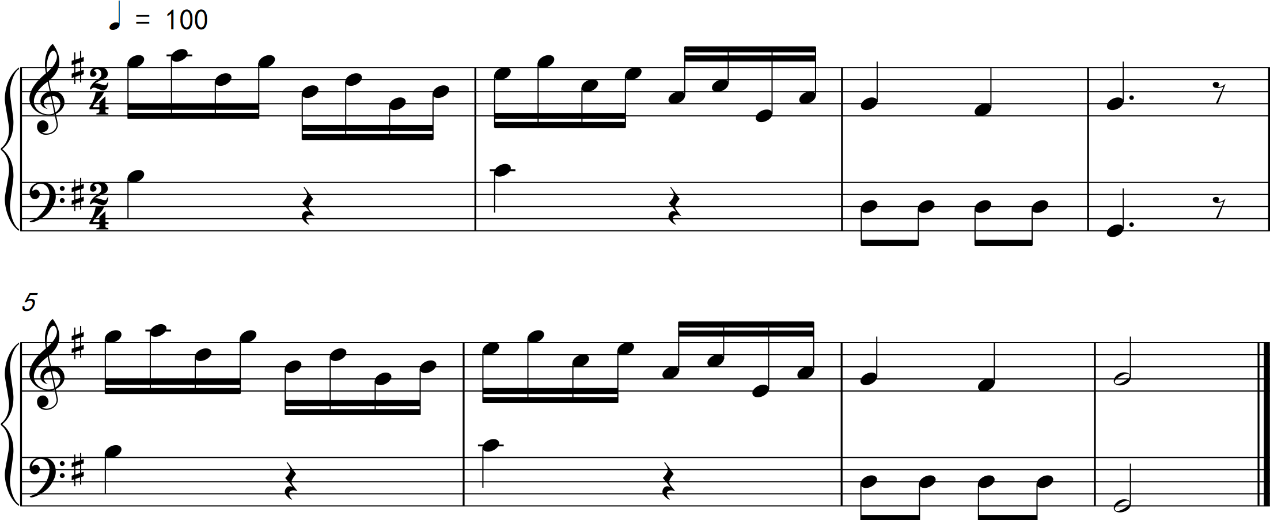
34
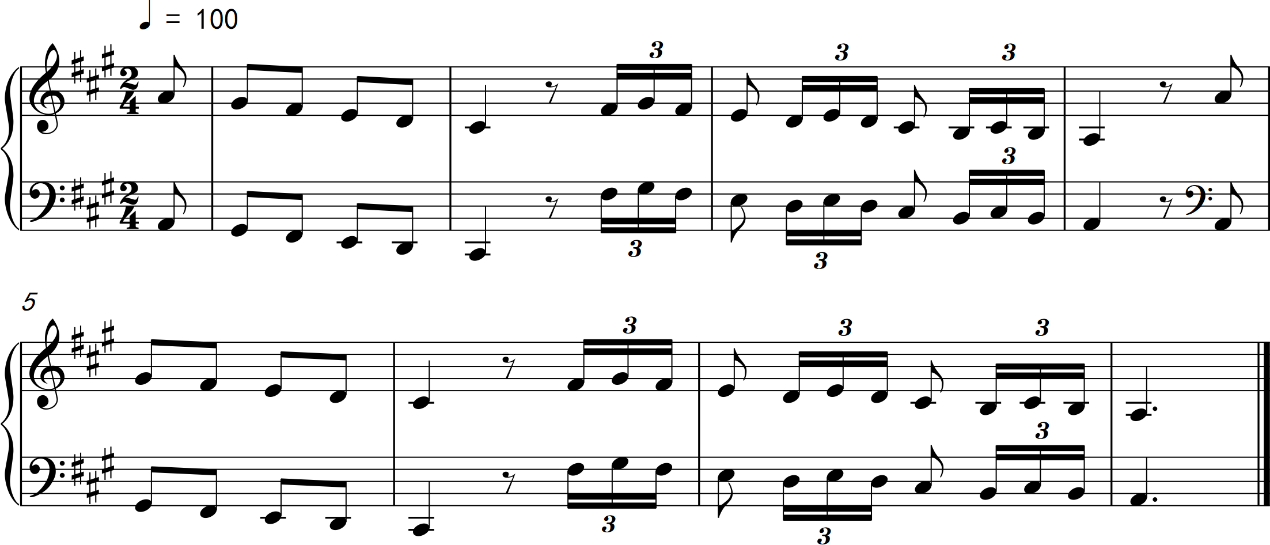
35
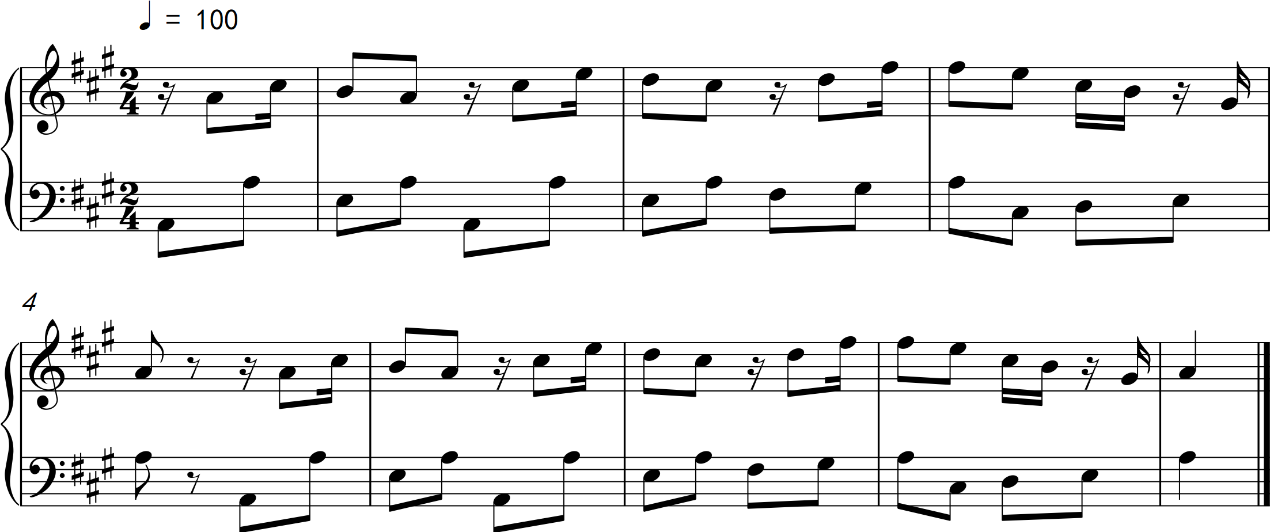
36
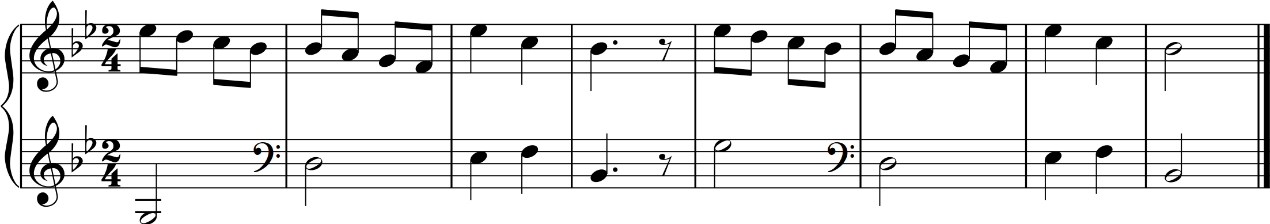
37
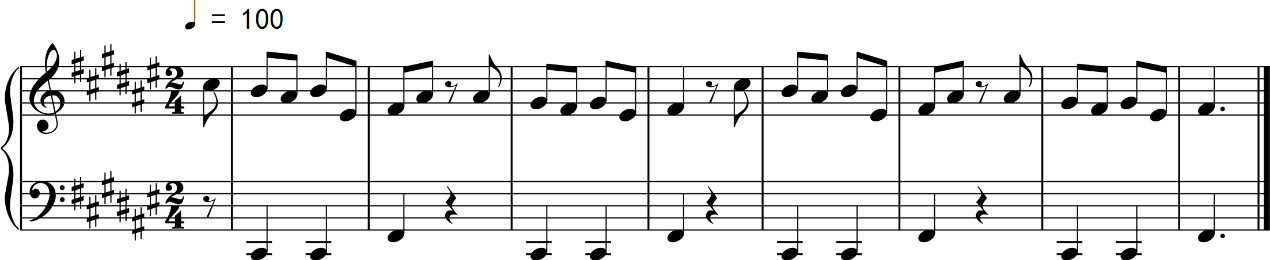
38
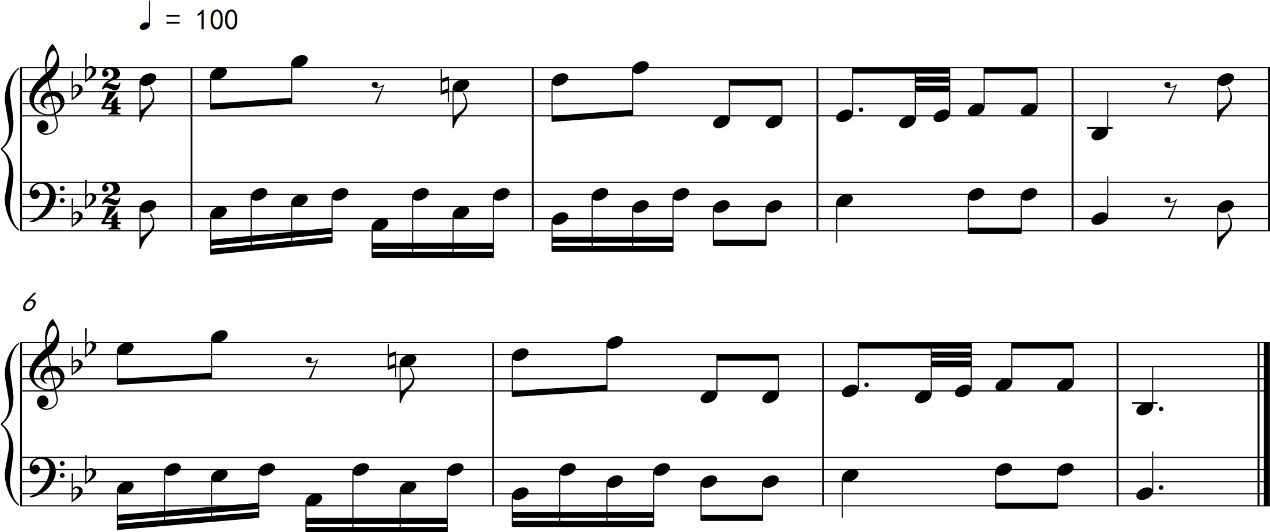
39
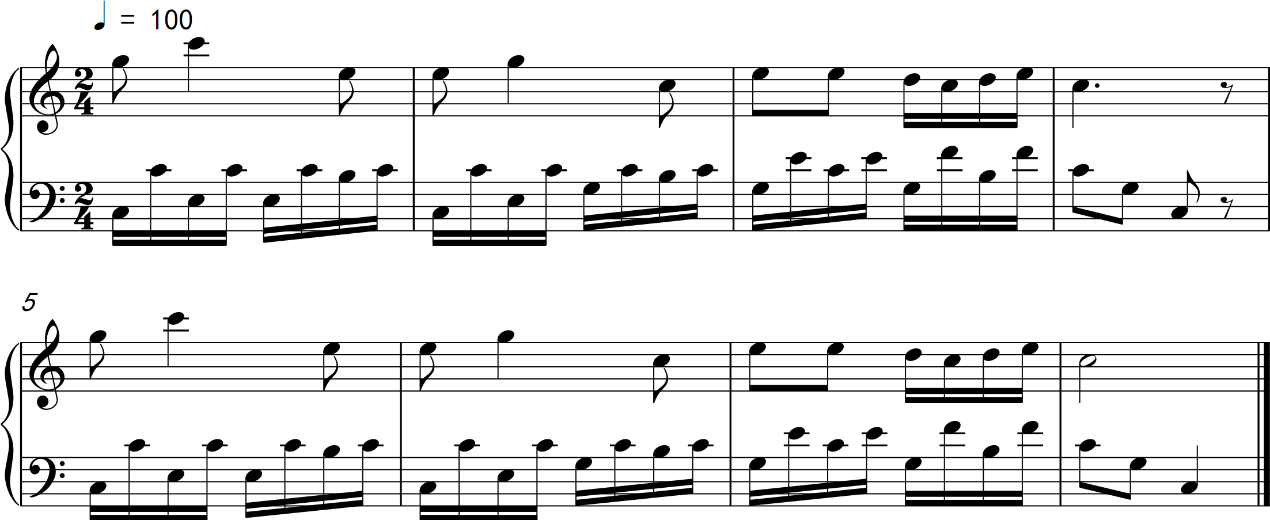
40
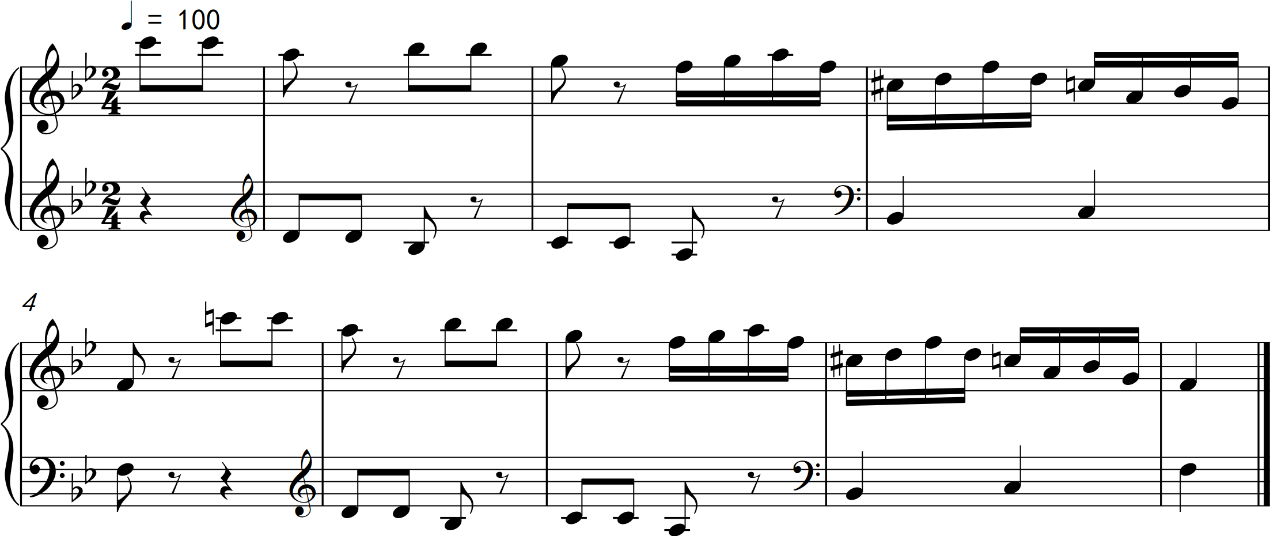
41
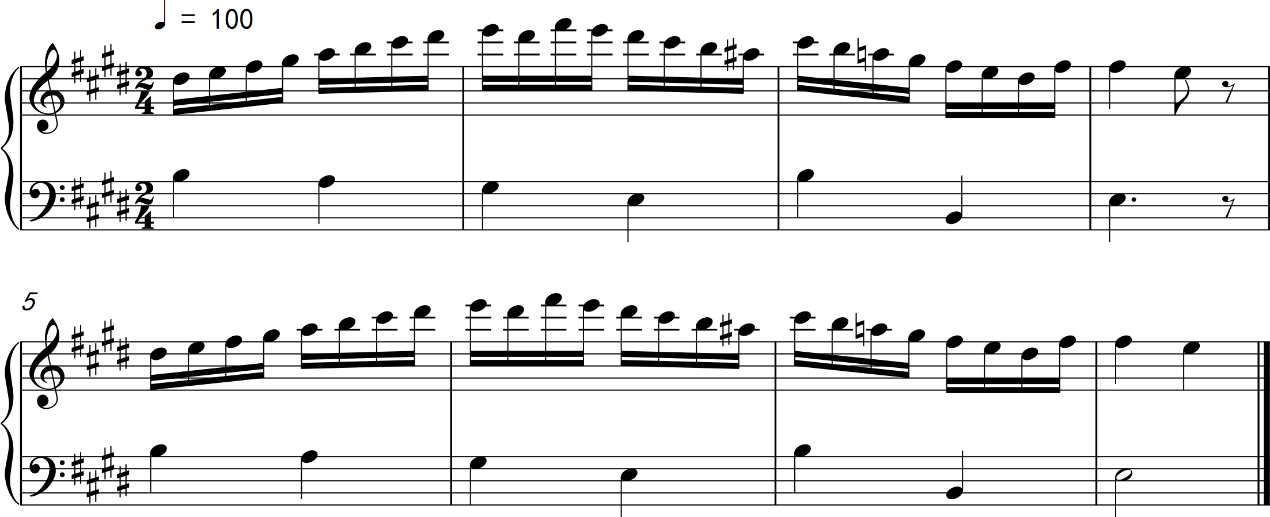
42
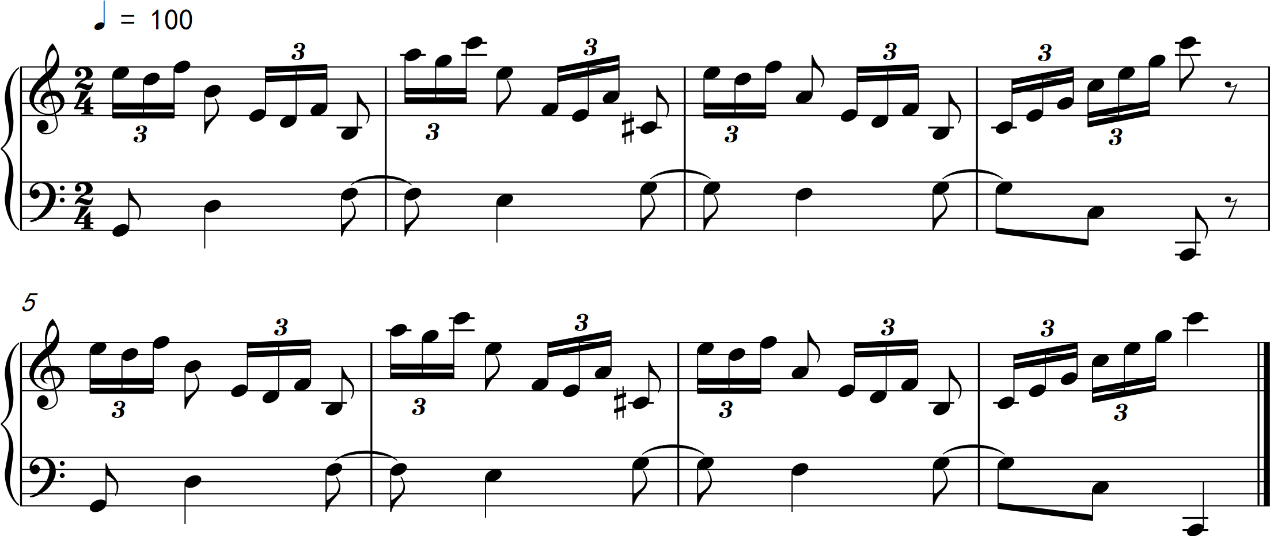
43
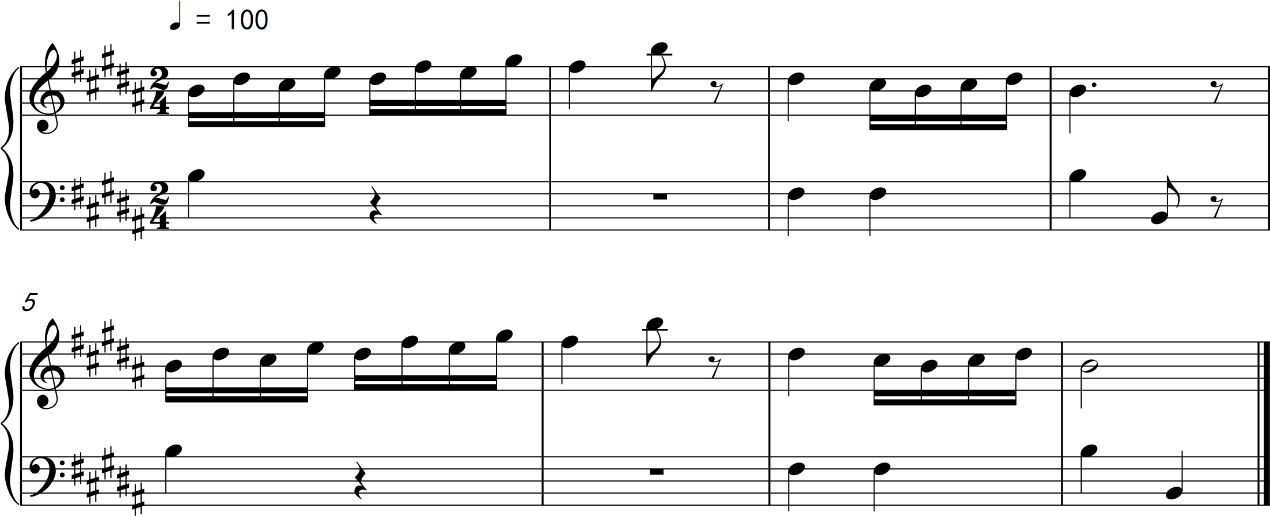
44
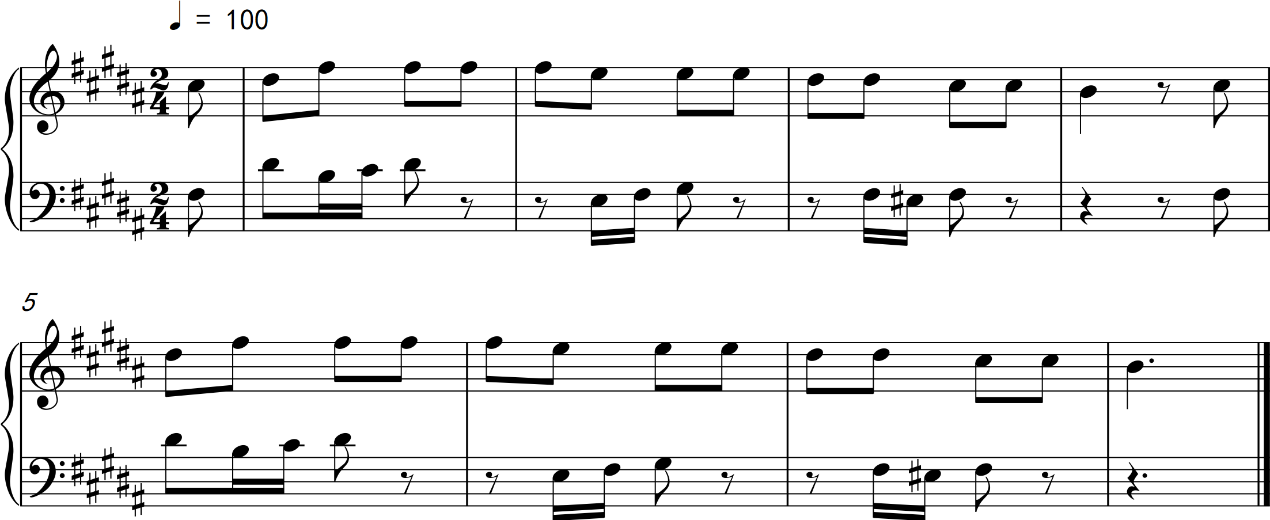
45
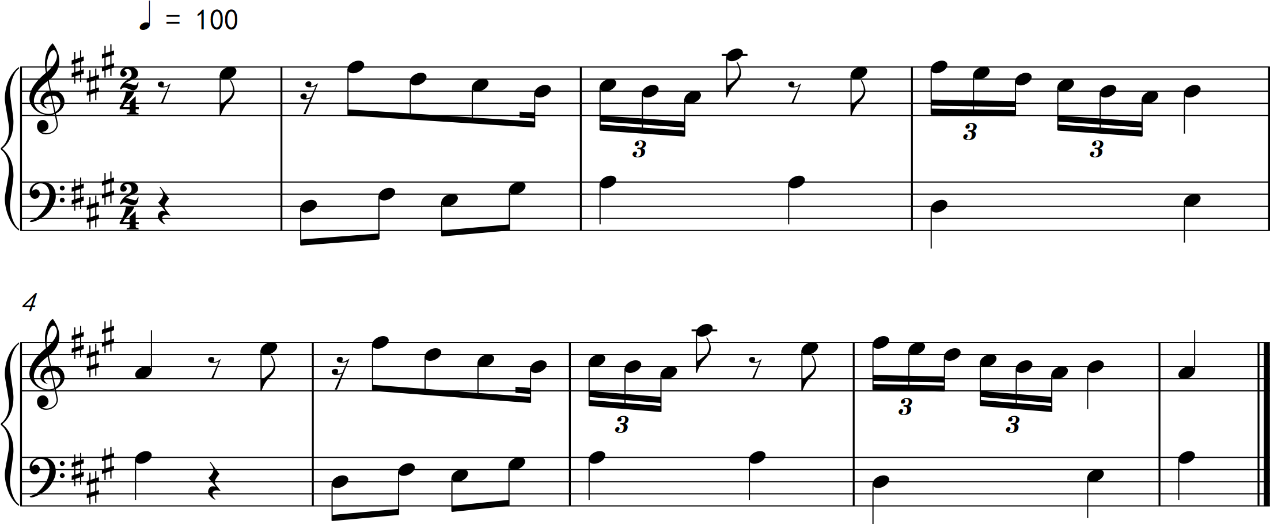
46
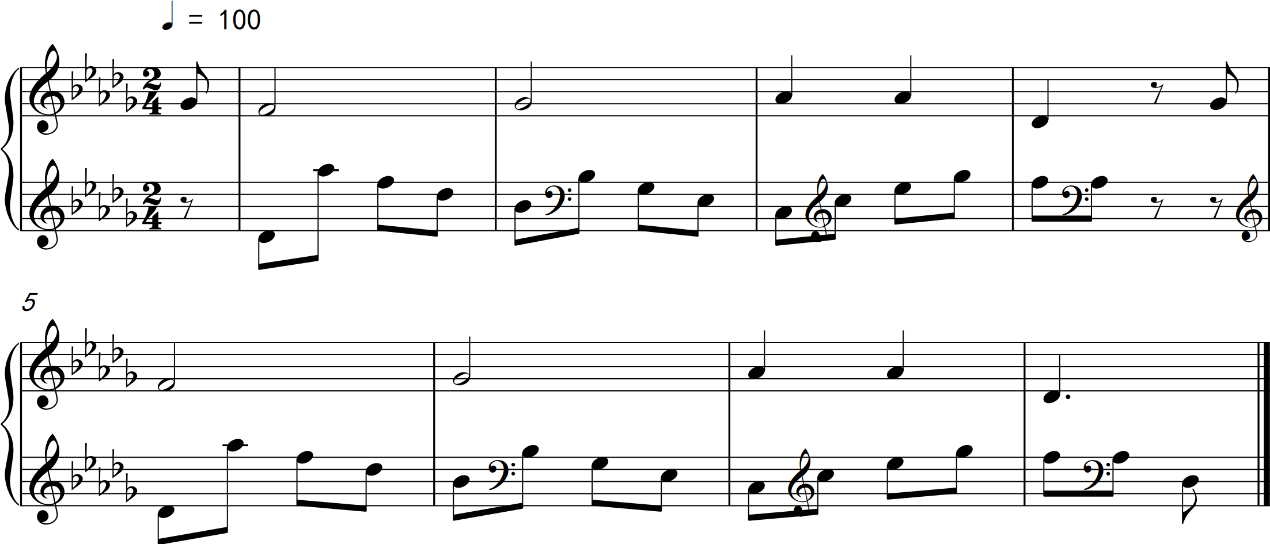
47
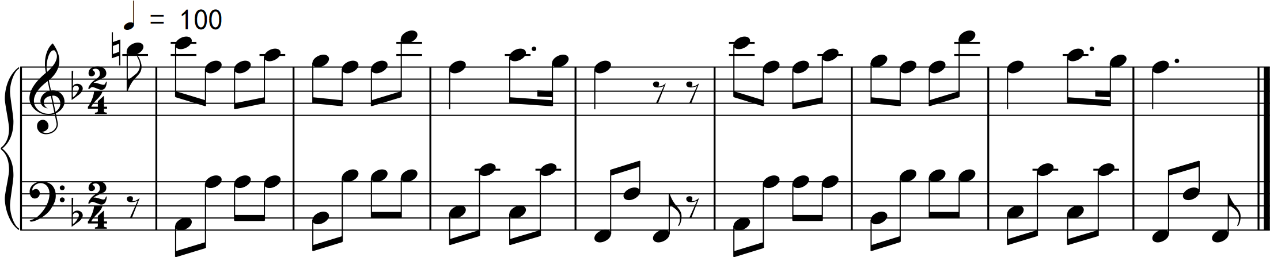
48
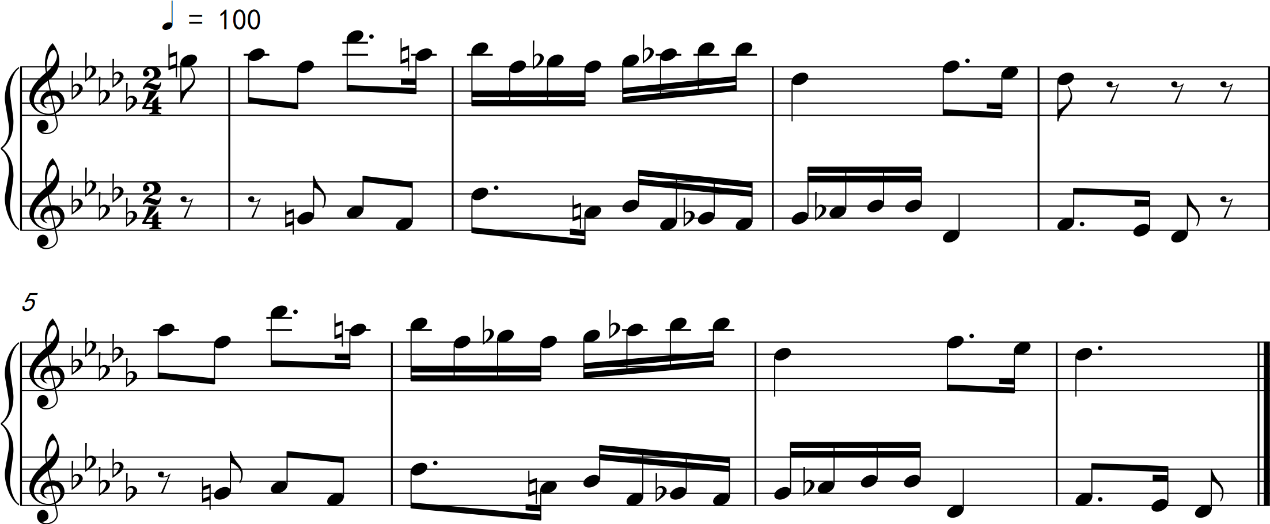
49
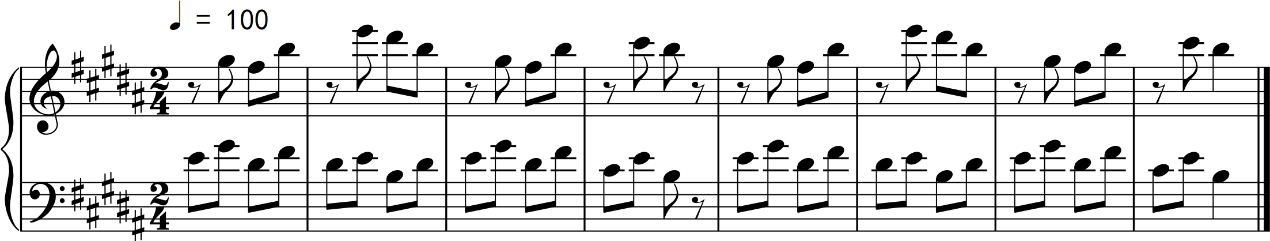
50
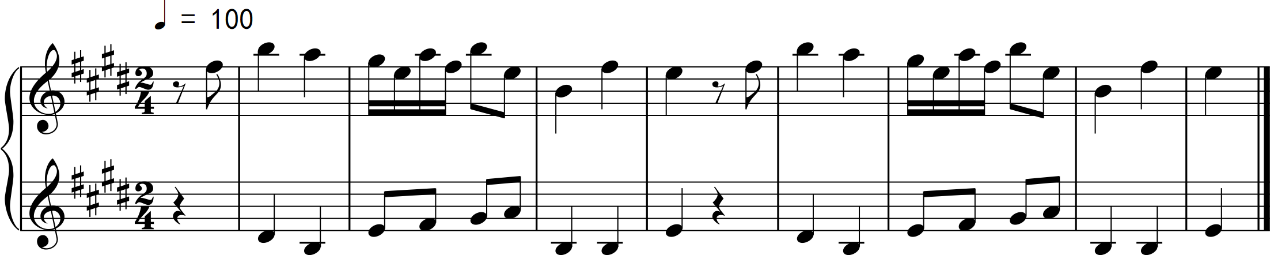
51
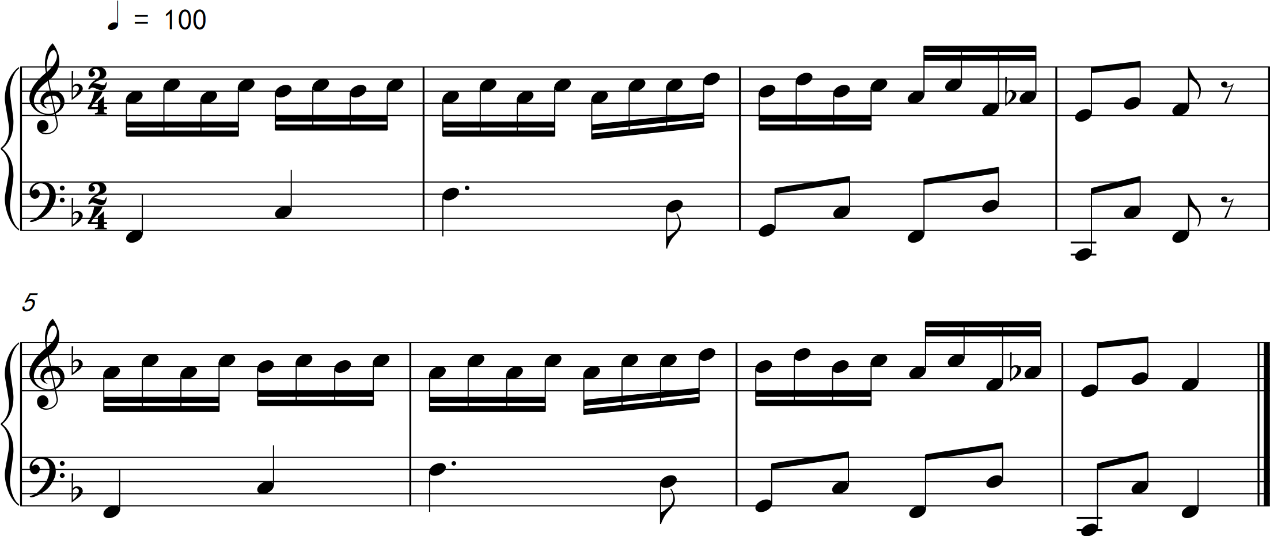
52
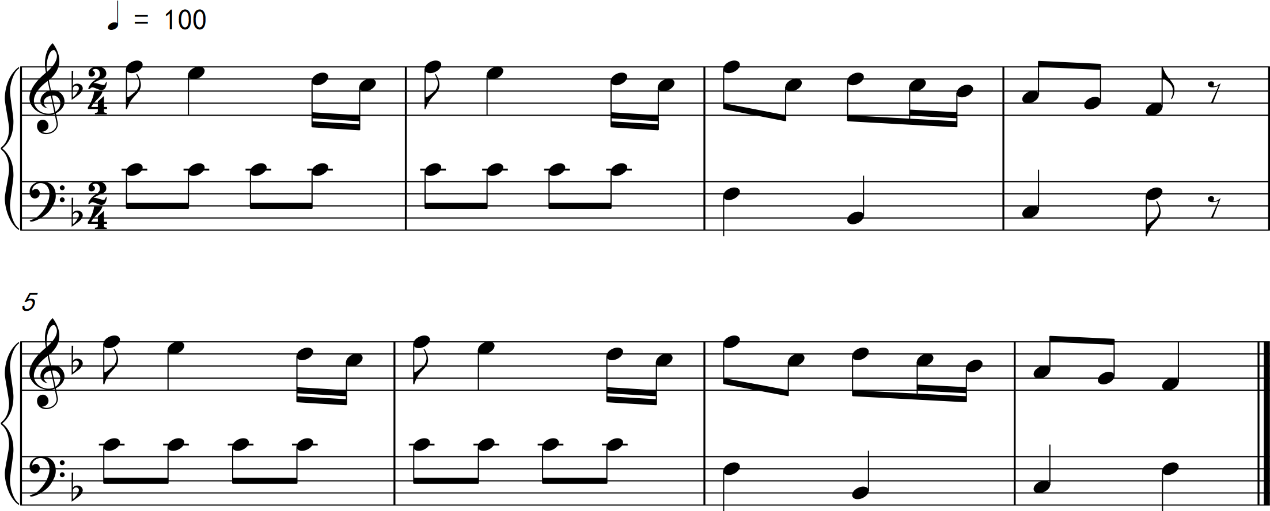
53
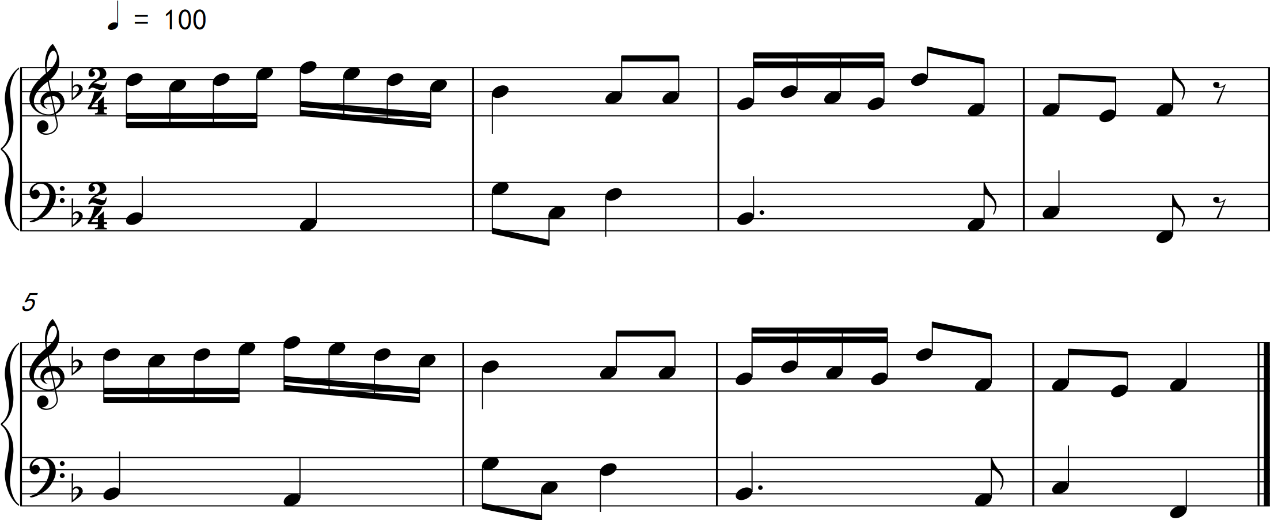
54
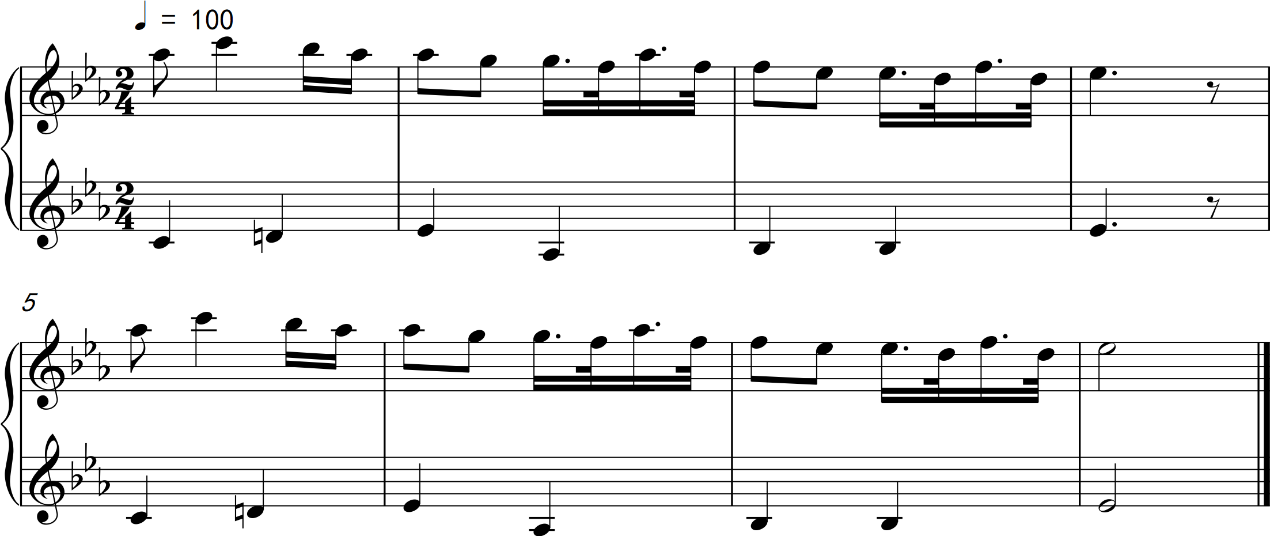
55
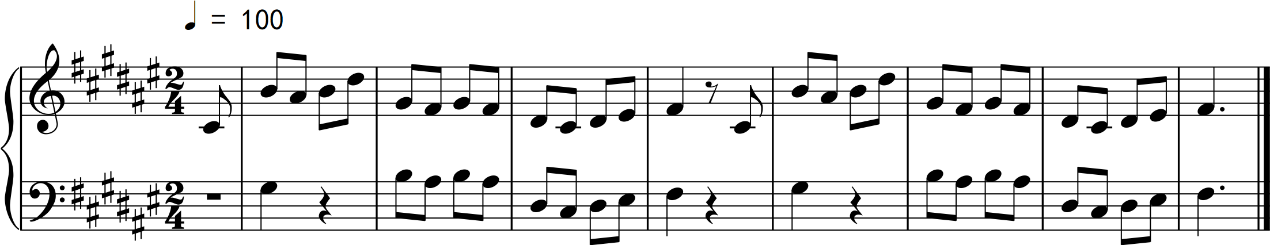
56
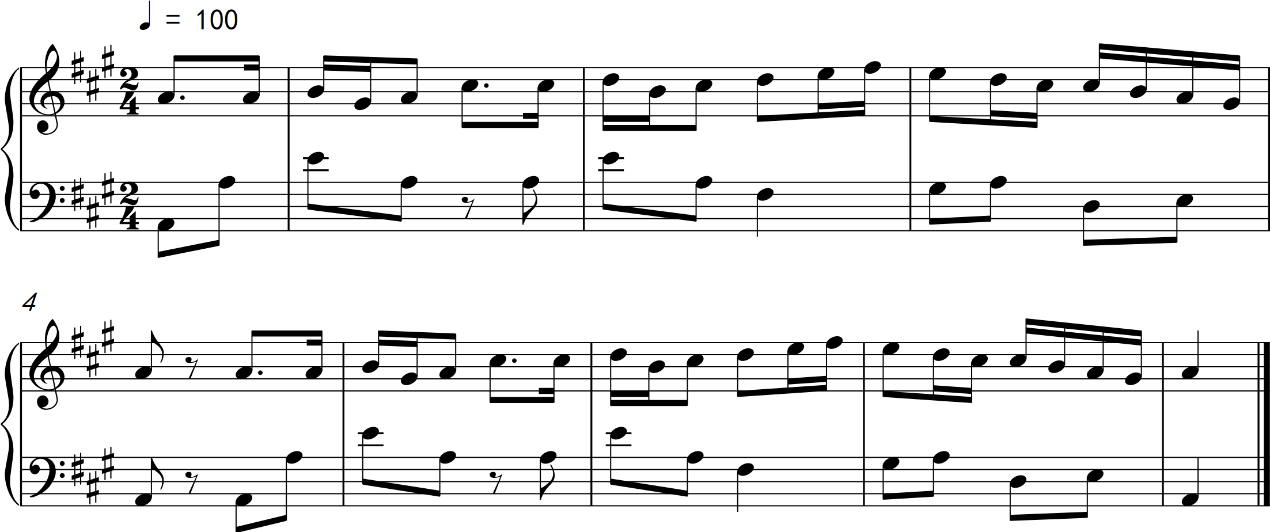
57
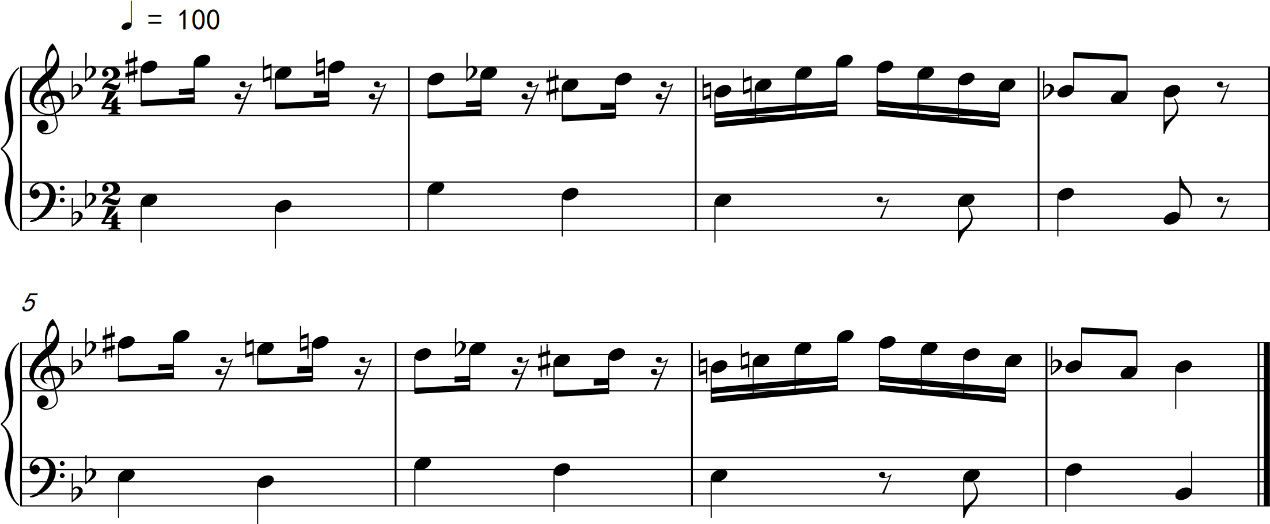
58
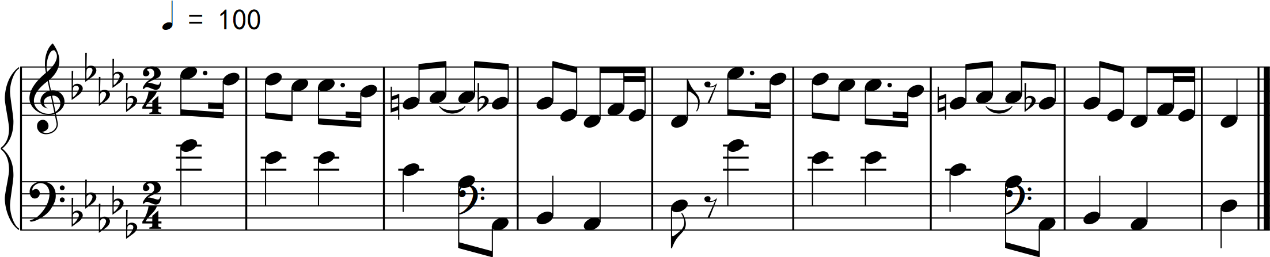
59
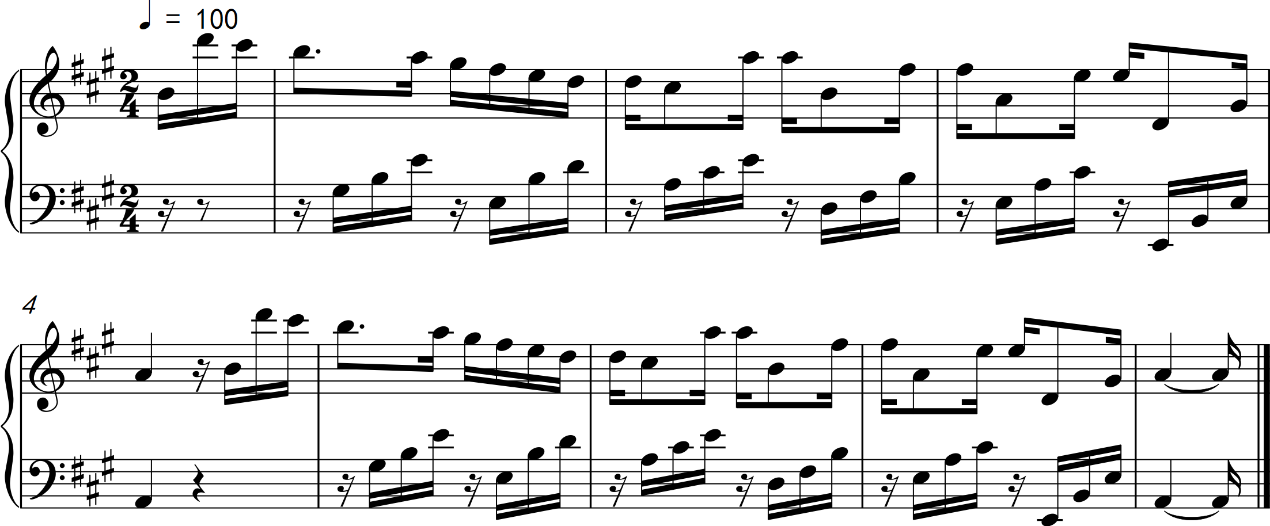
60
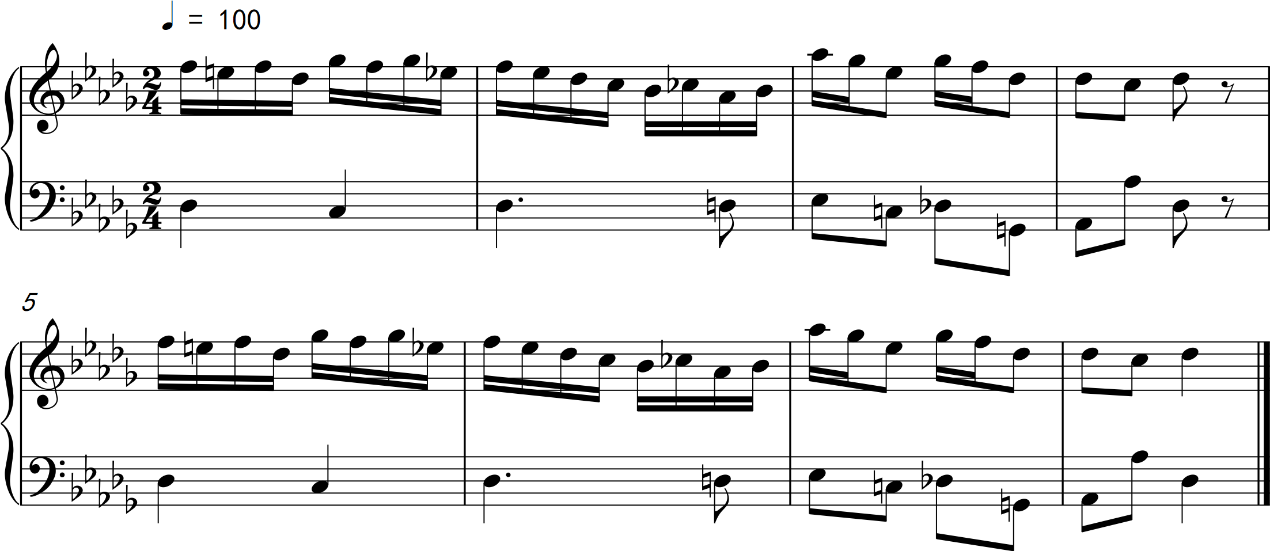

Supplement: Supplementary file 2 — Supplementary Information 2. [file 41598_2022_11949_MOESM2_ESM.docx]

1
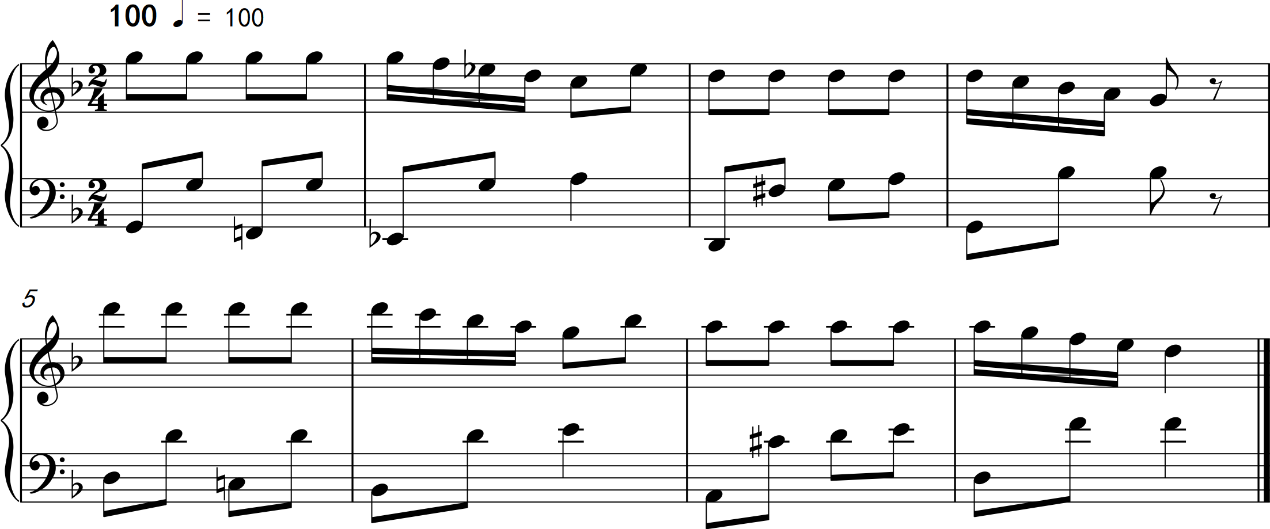
2
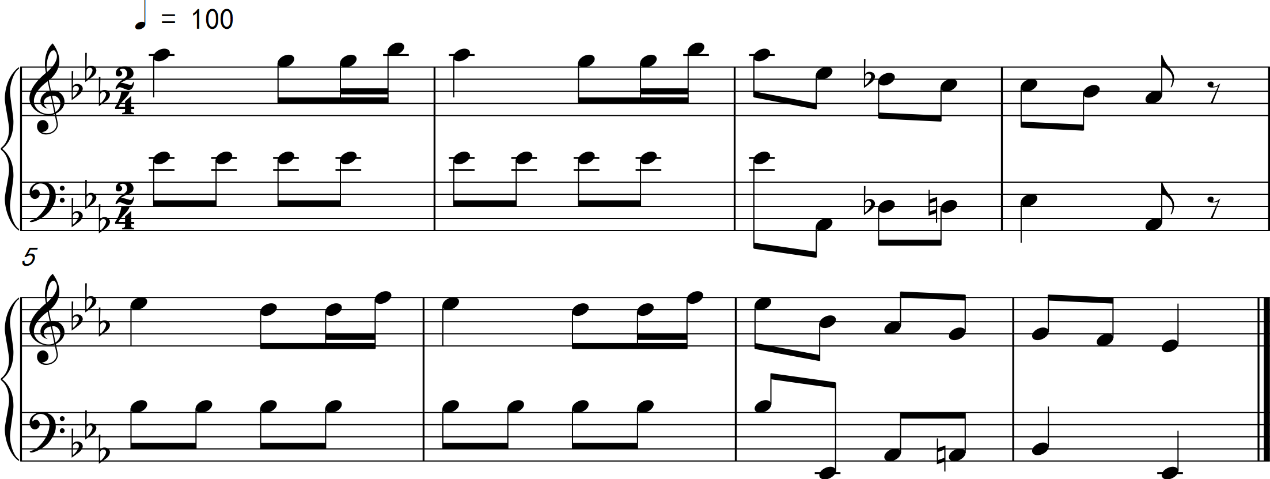
3
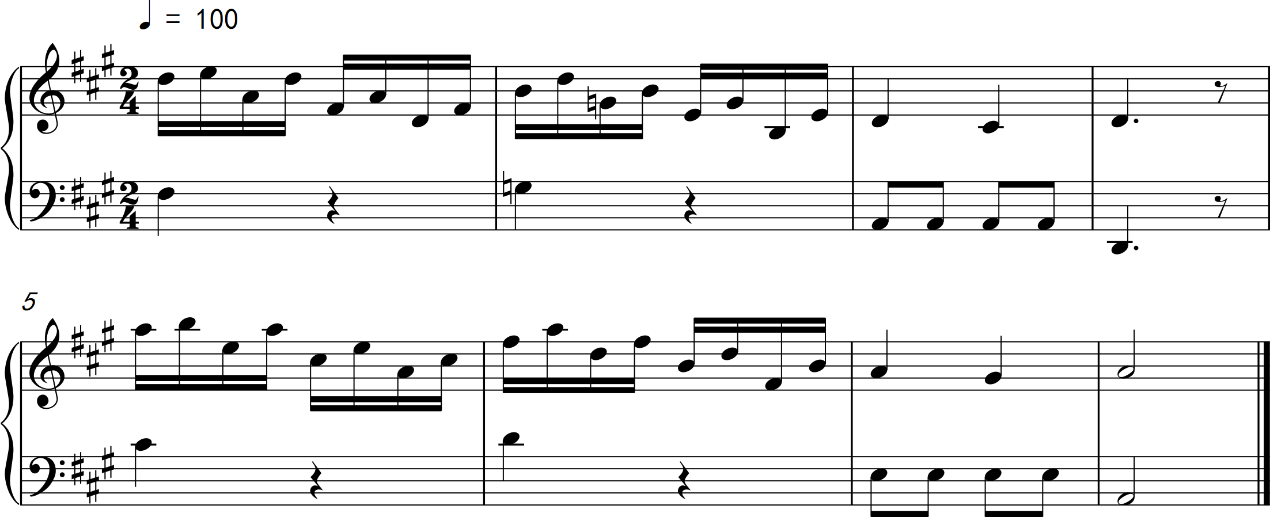
4
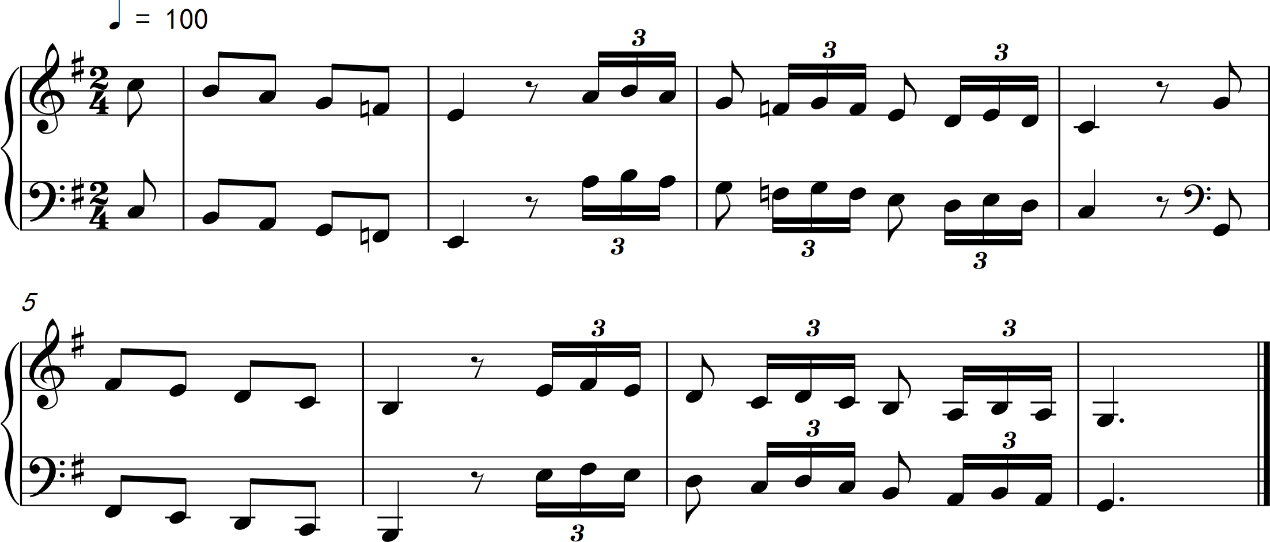
5
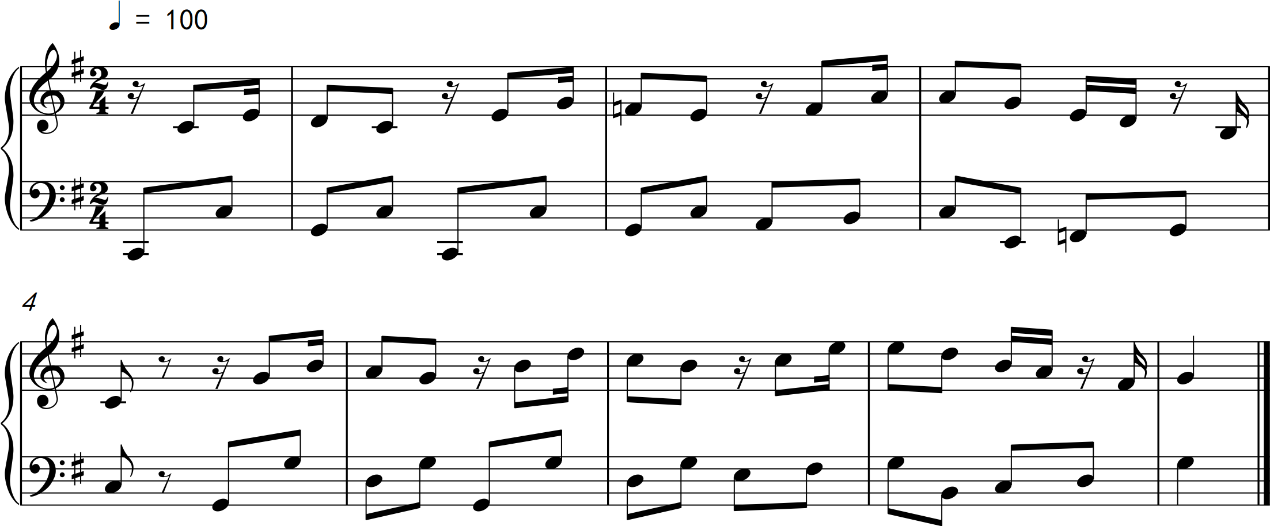
6
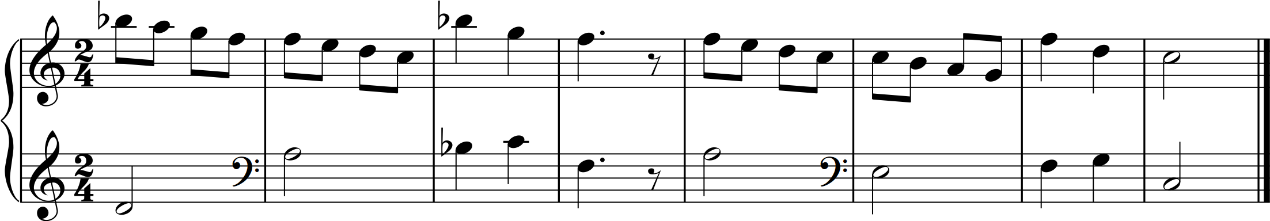
7
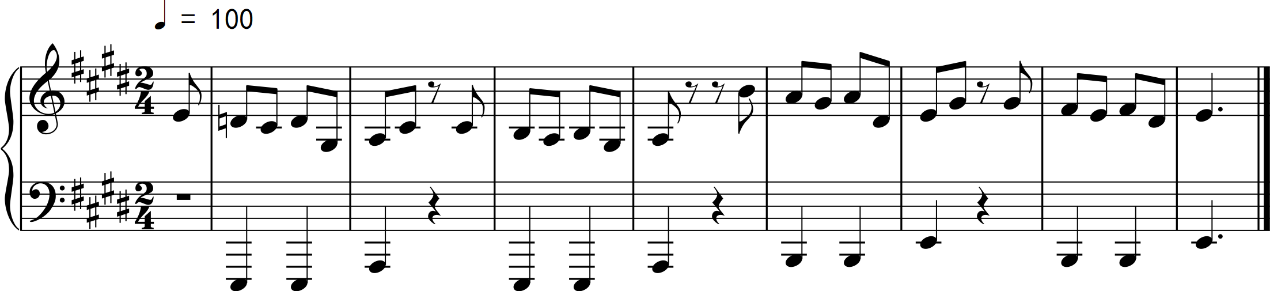
8
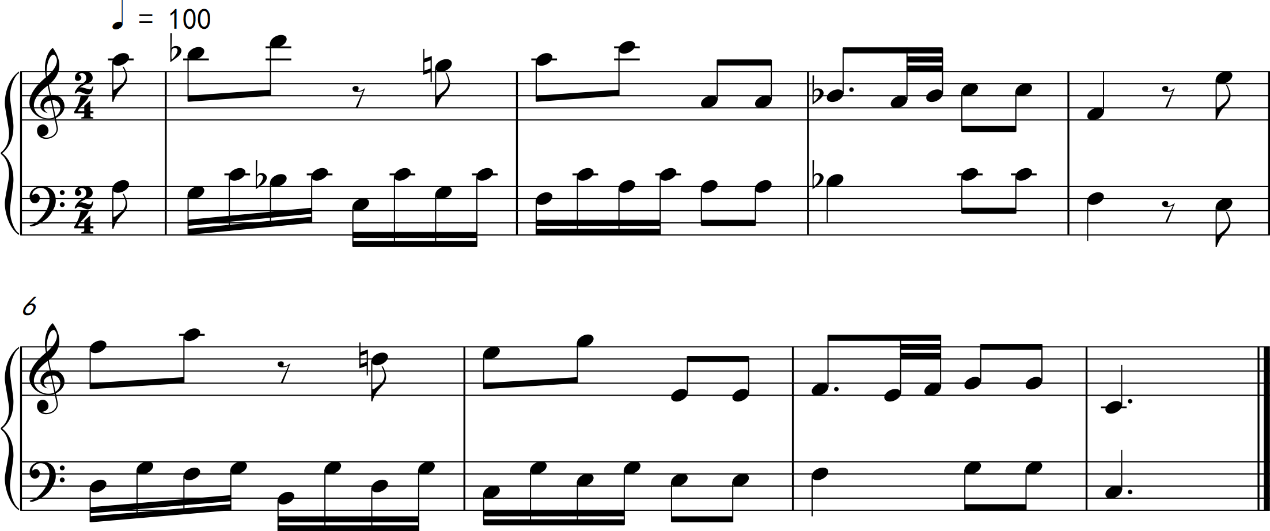
9
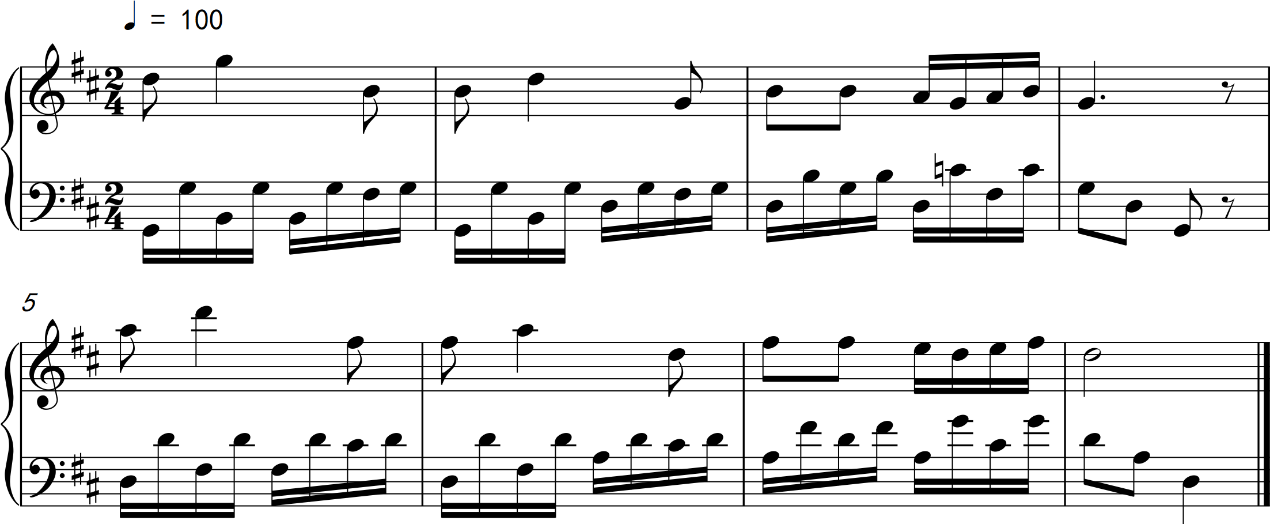
10
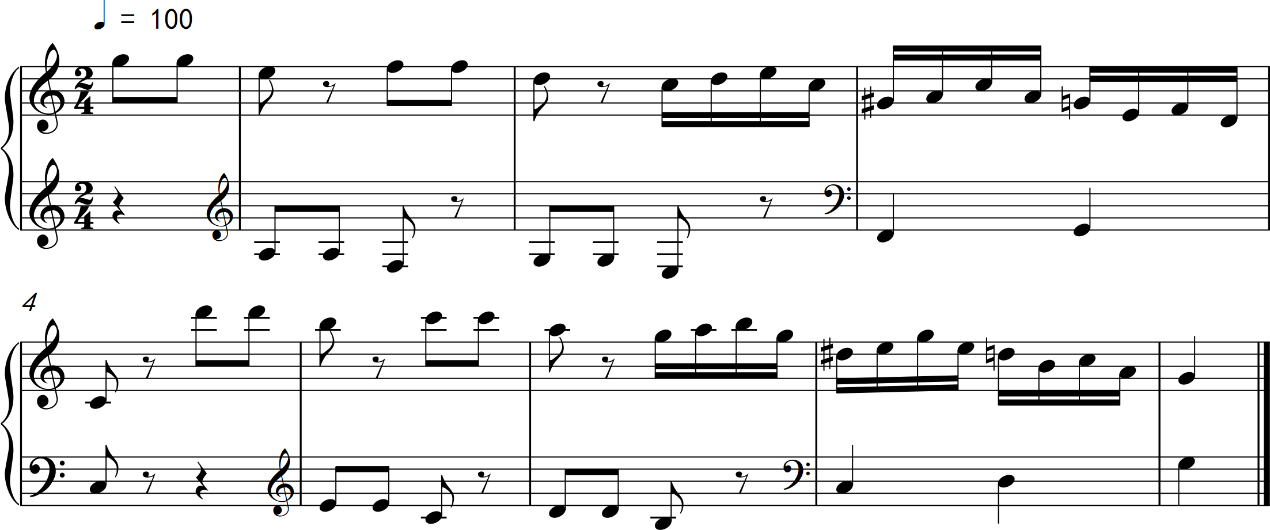
11
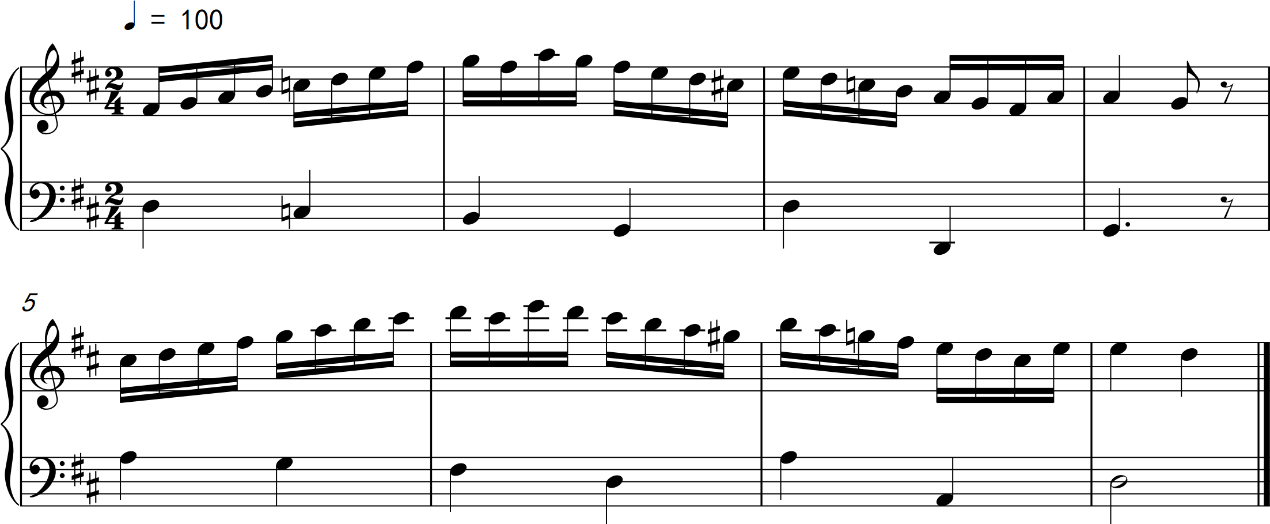
12
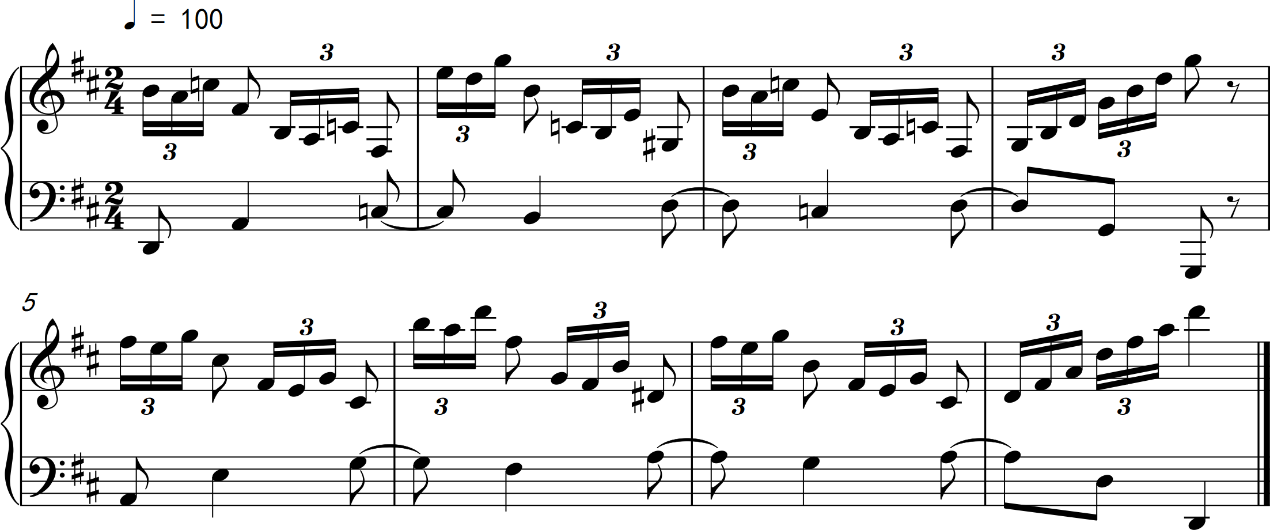
13
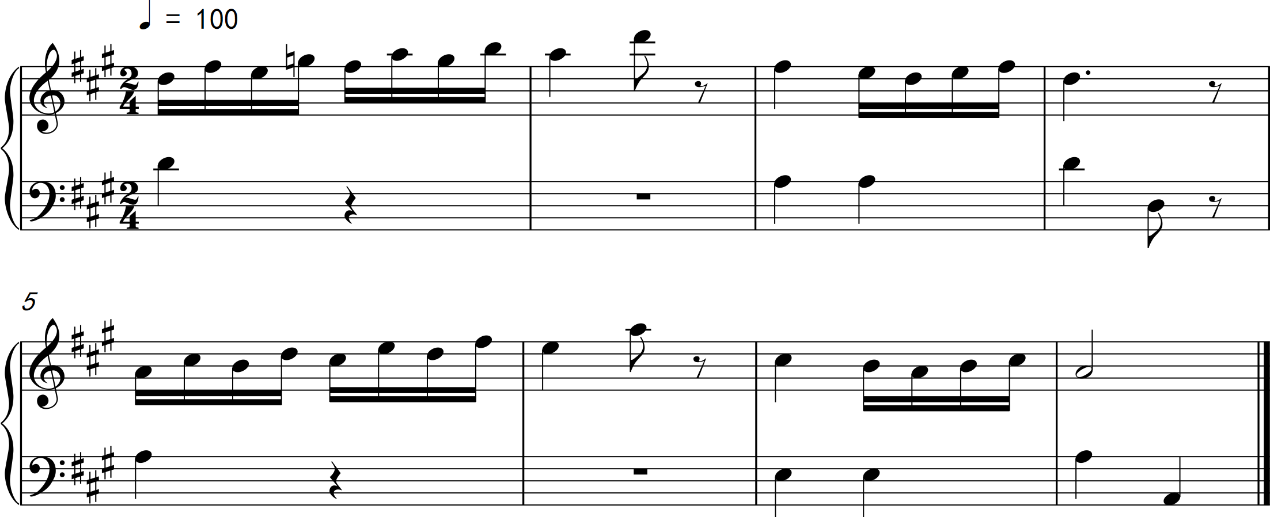
14
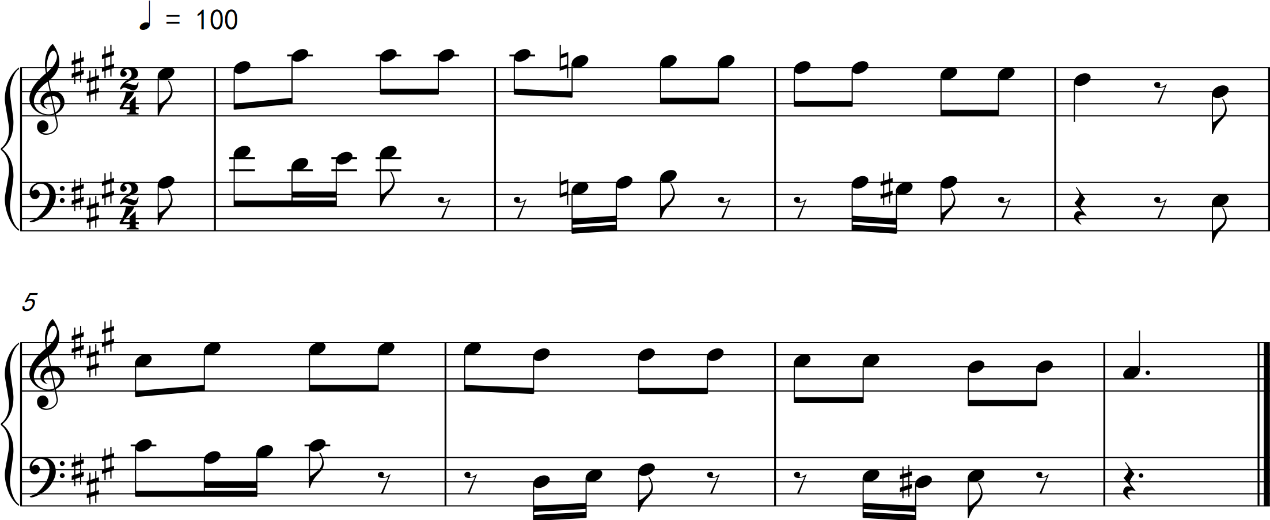
15
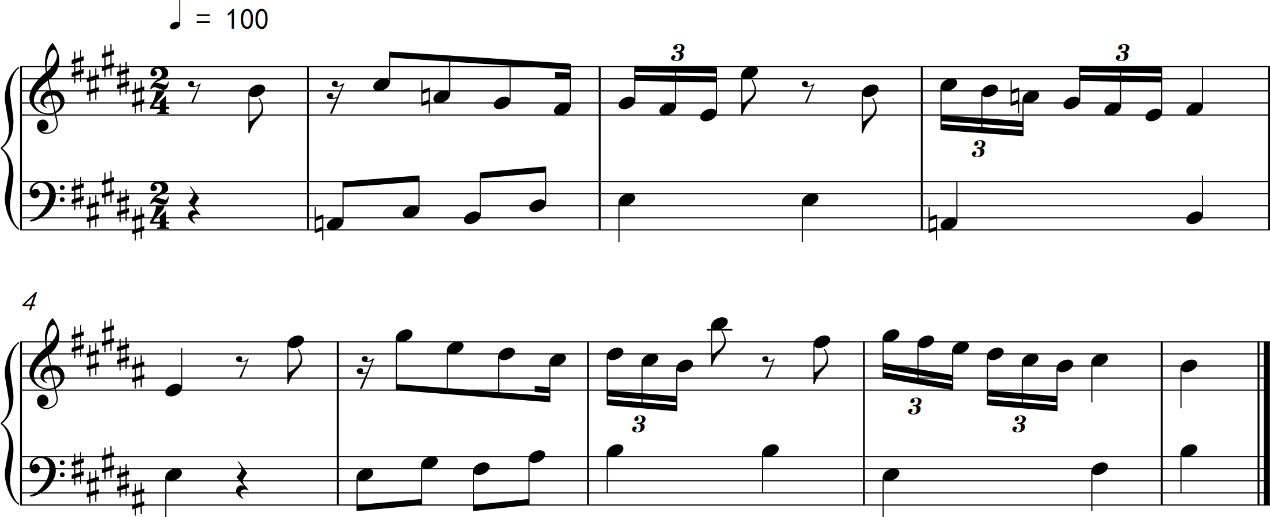
16
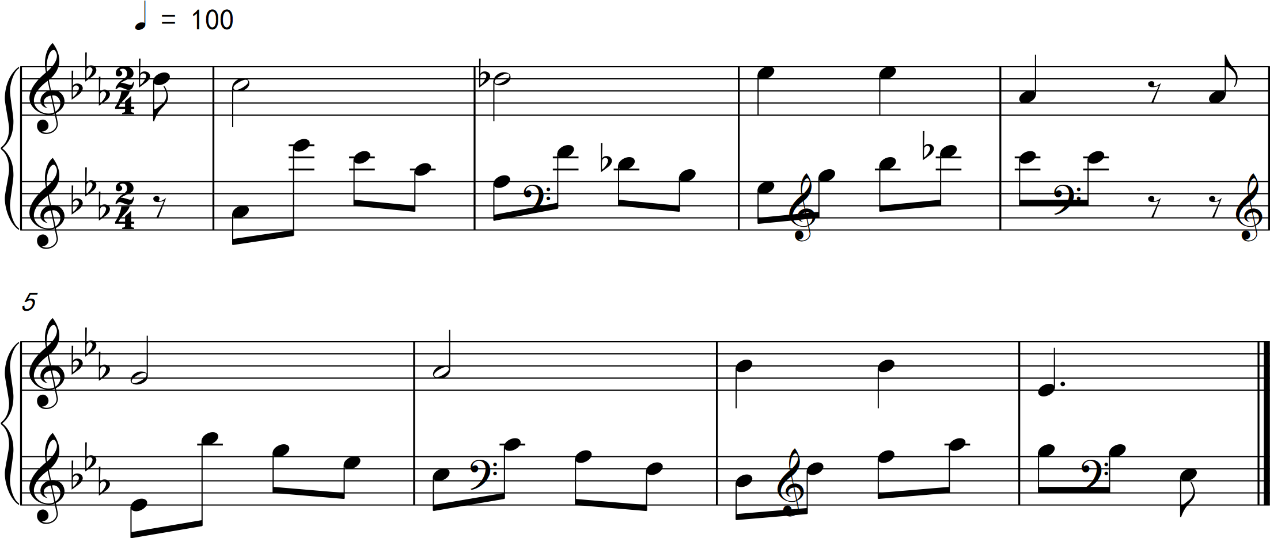
17
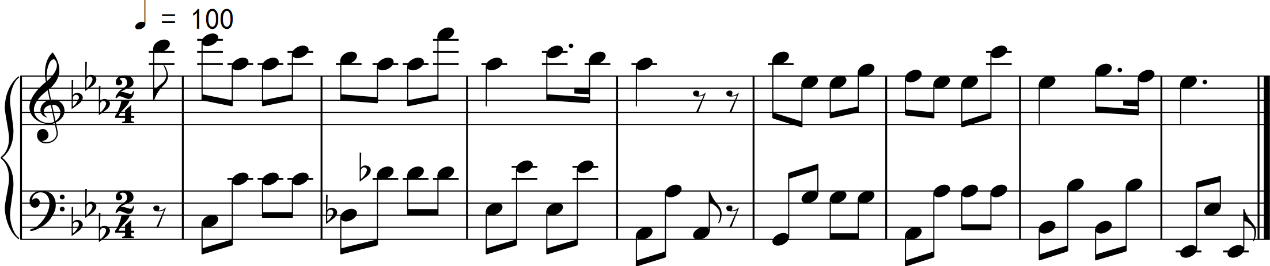
18
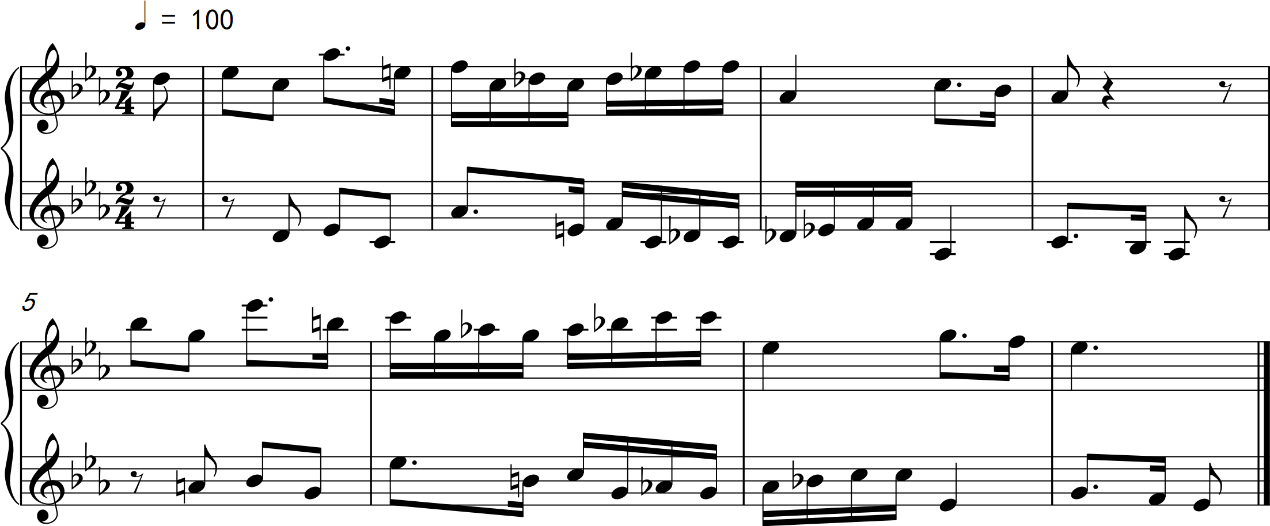
19
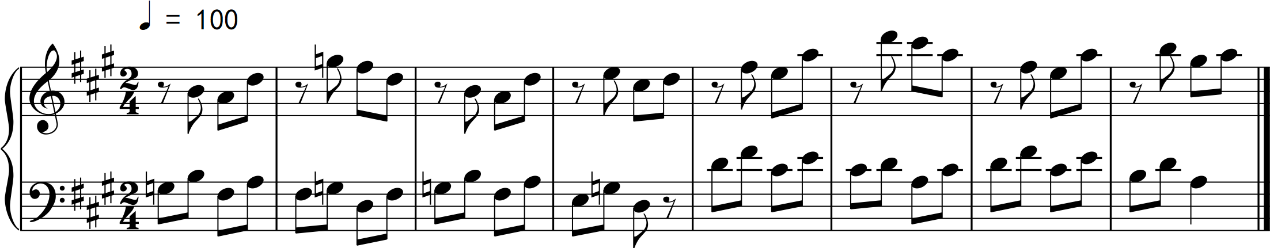
20
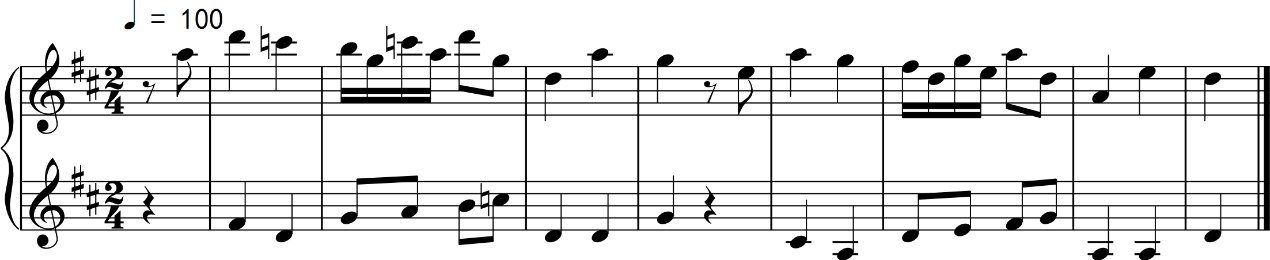
21
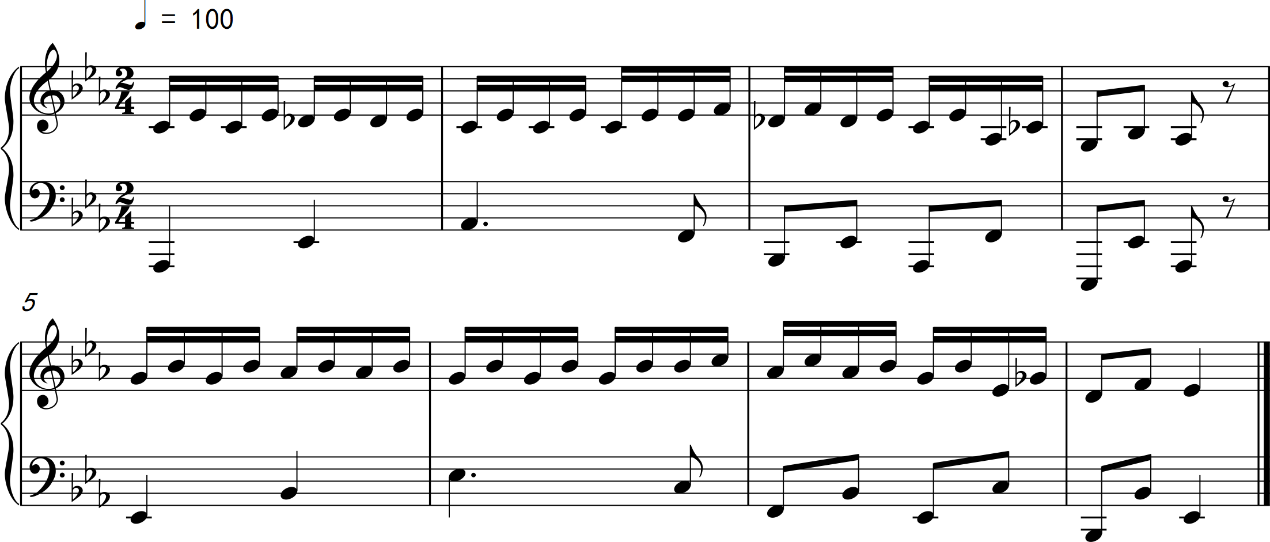
22
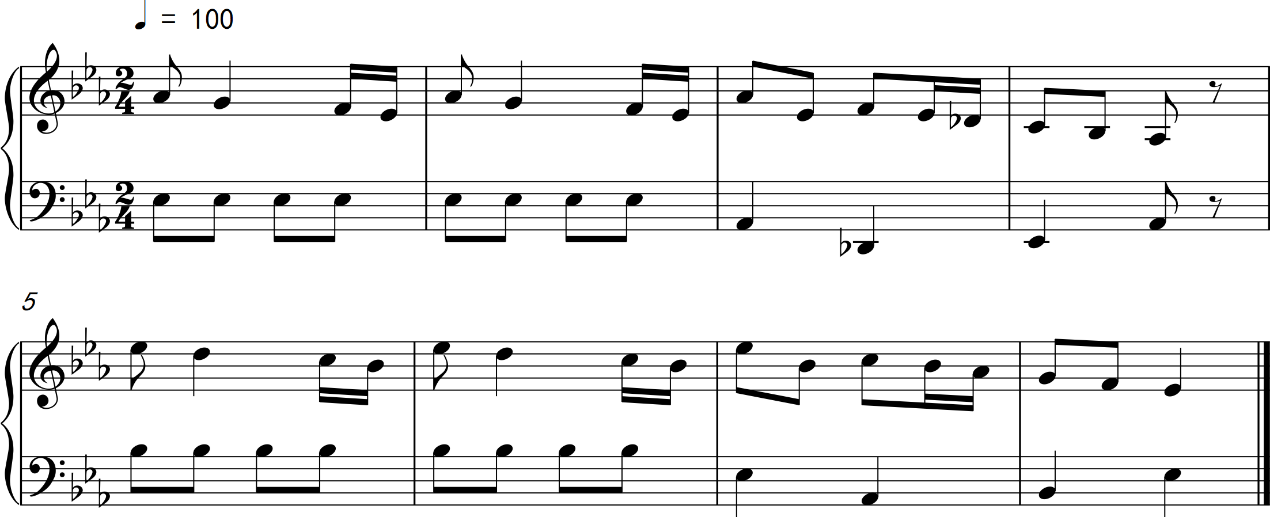
23
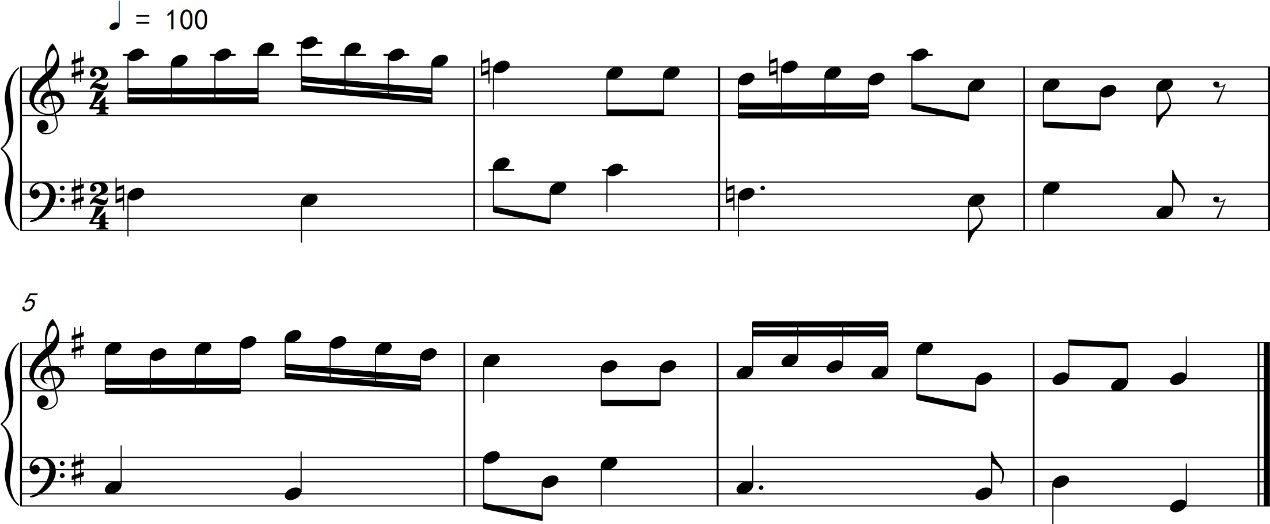
24
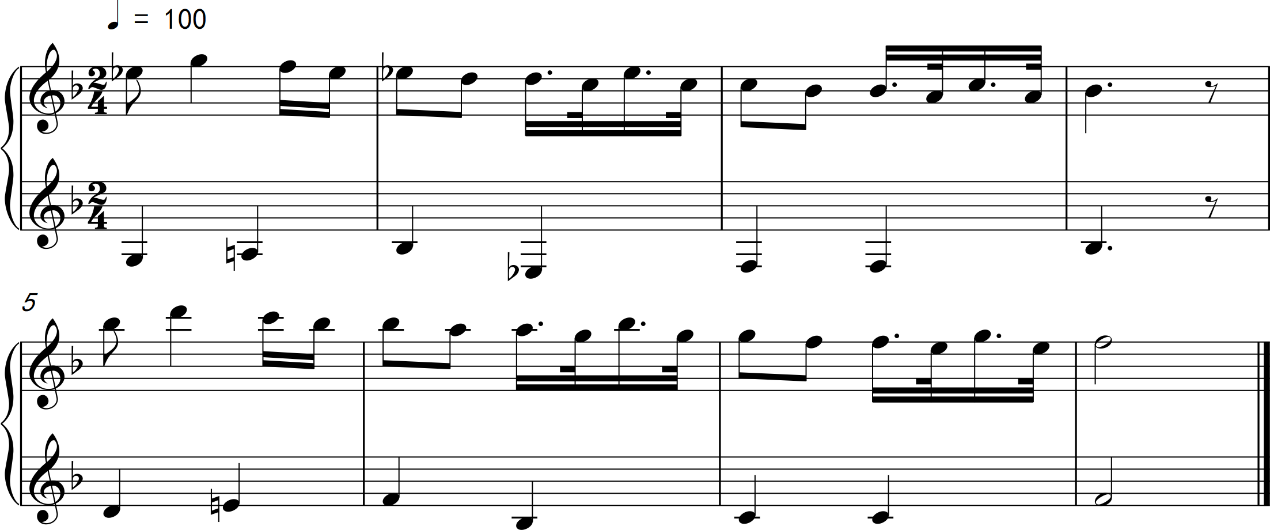
25
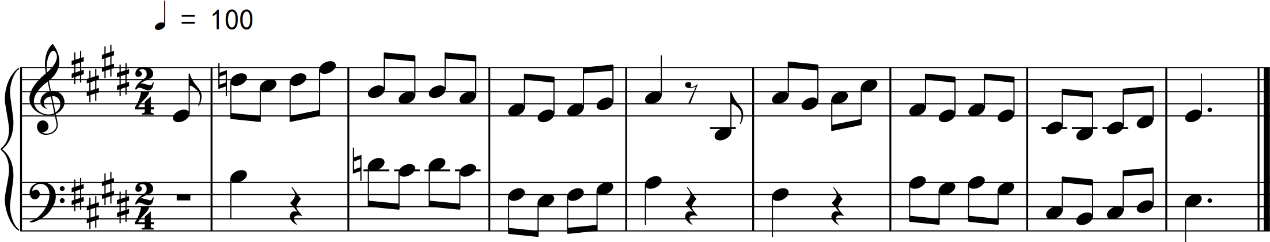
26
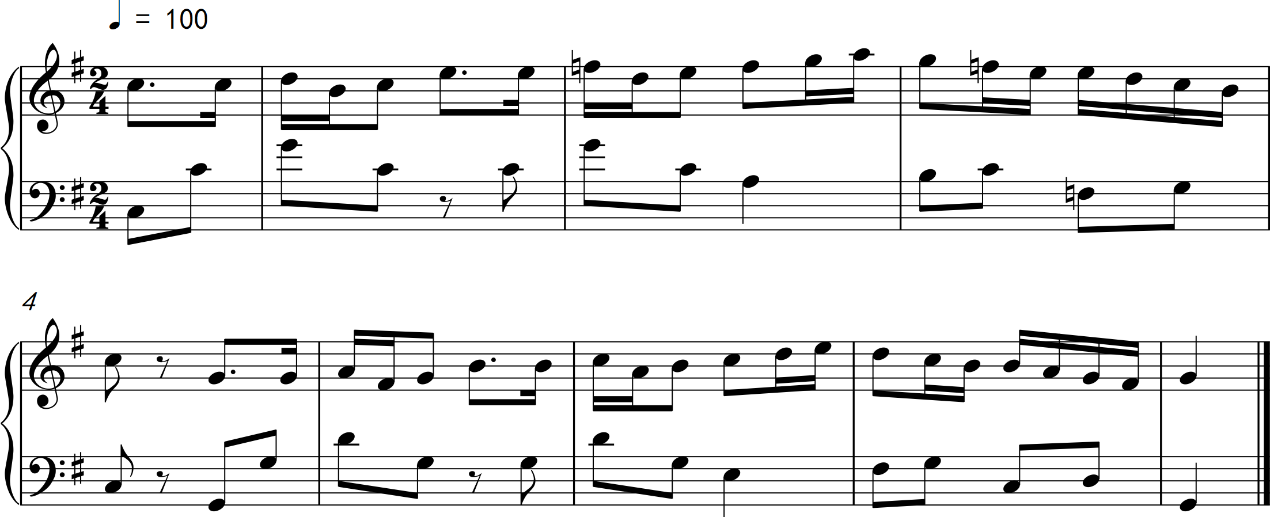
27
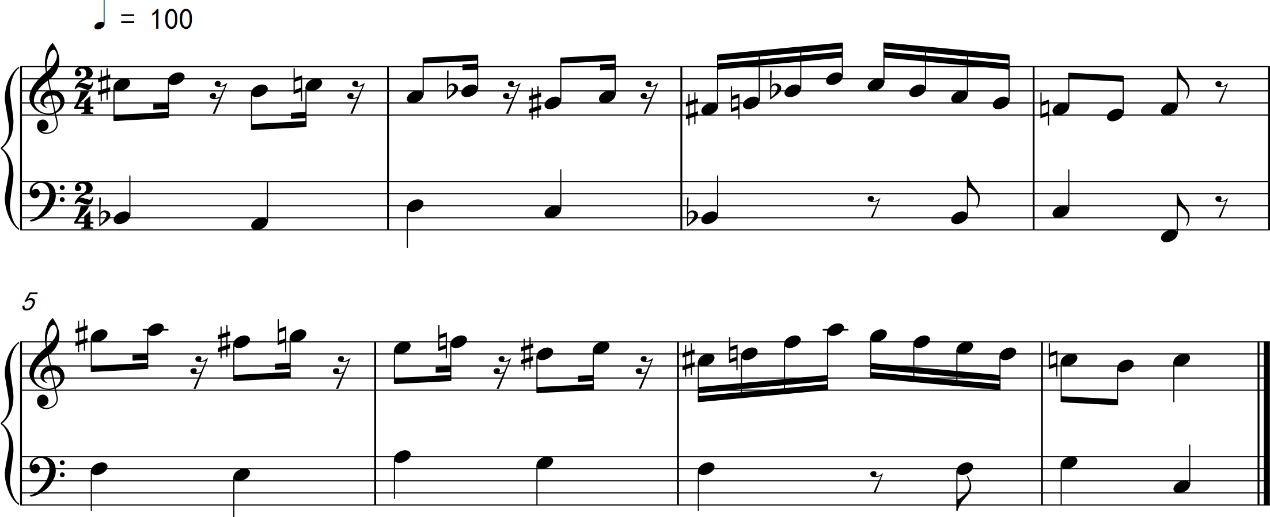
28
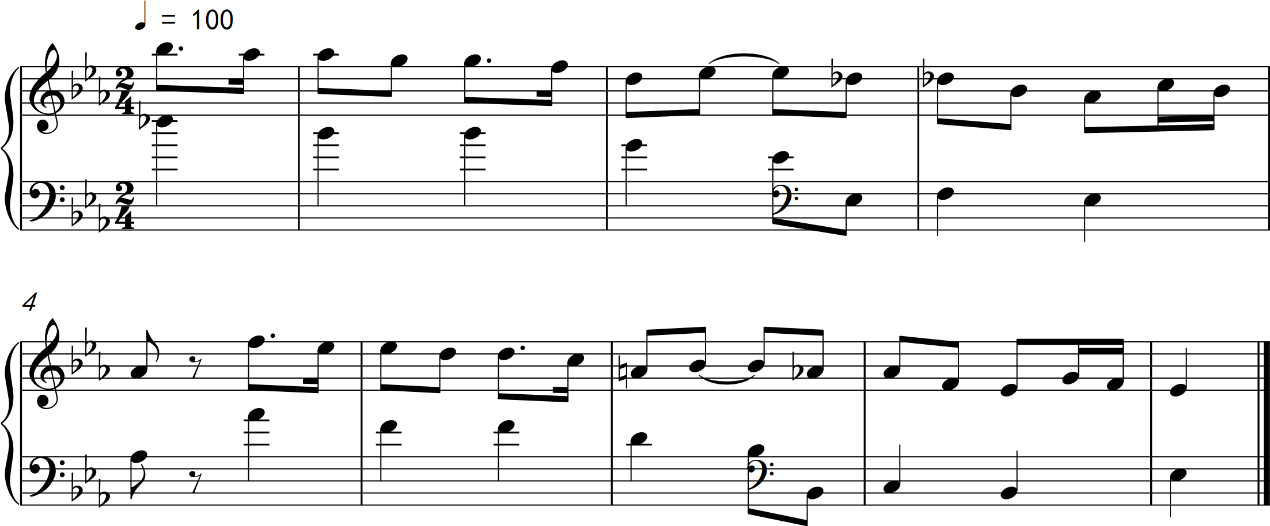
29
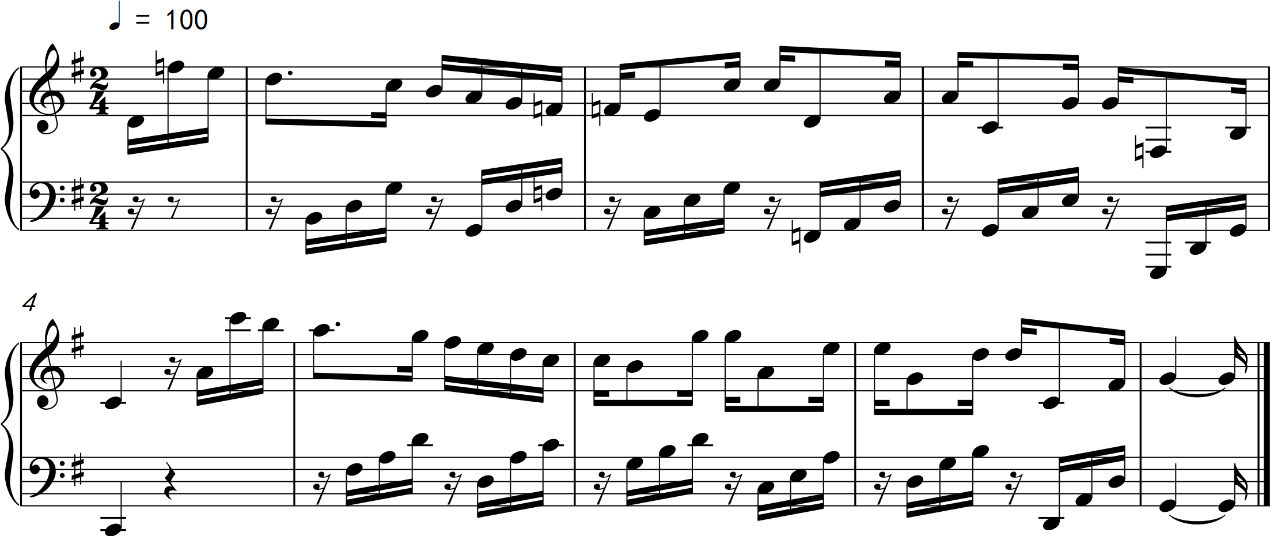
30
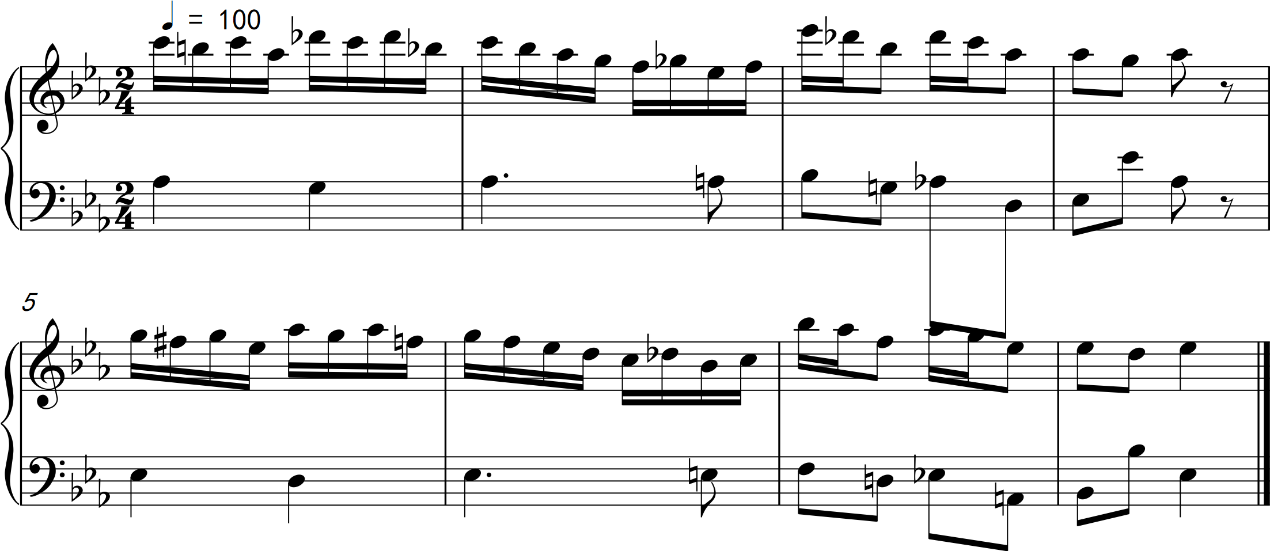
31
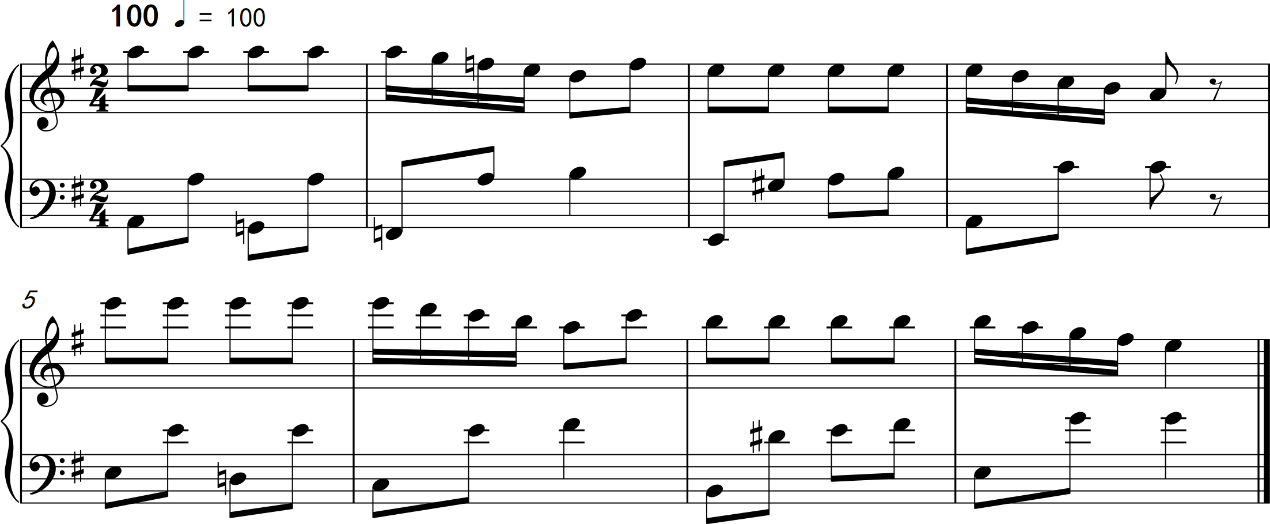
32
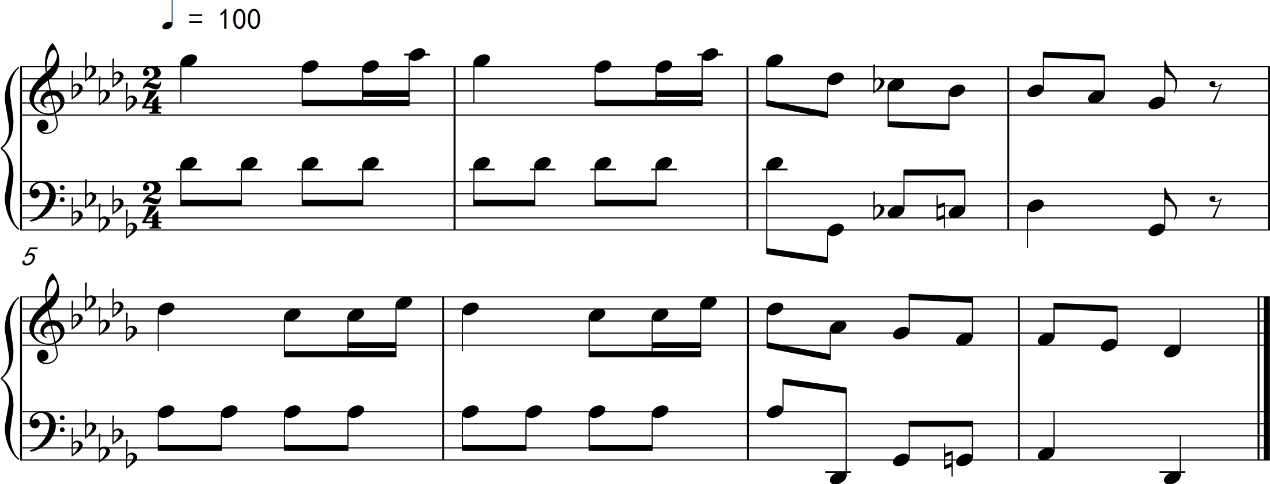
33
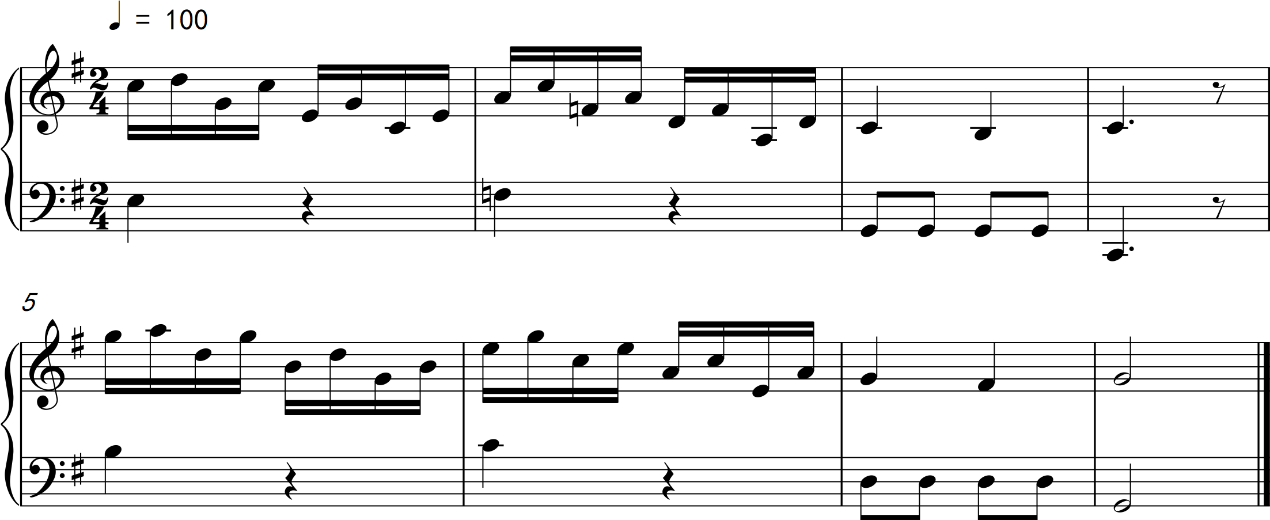
34
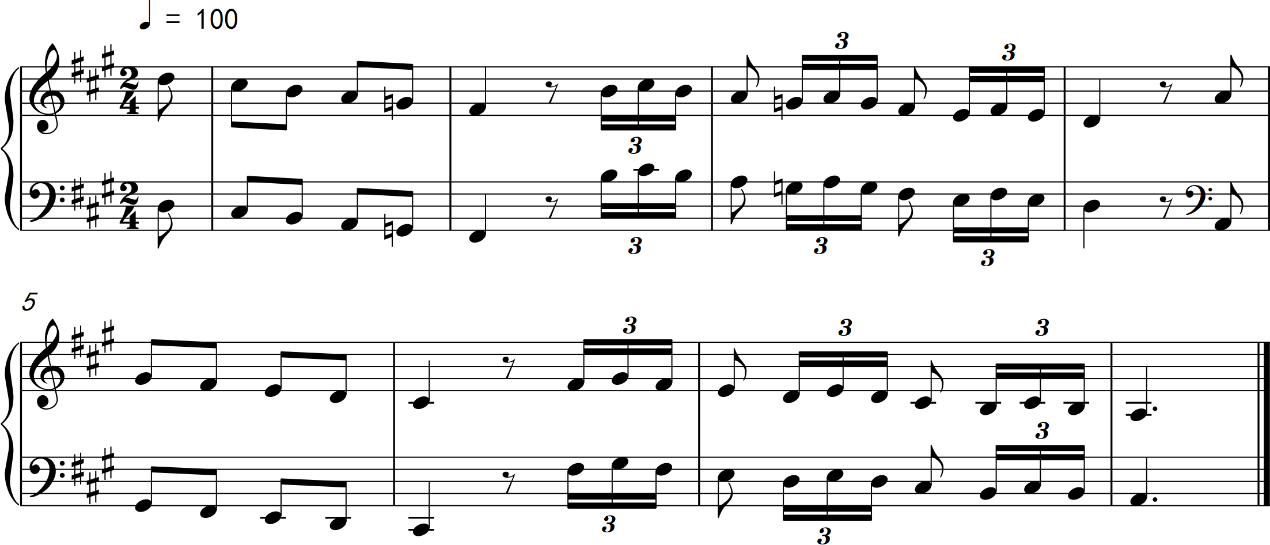
35
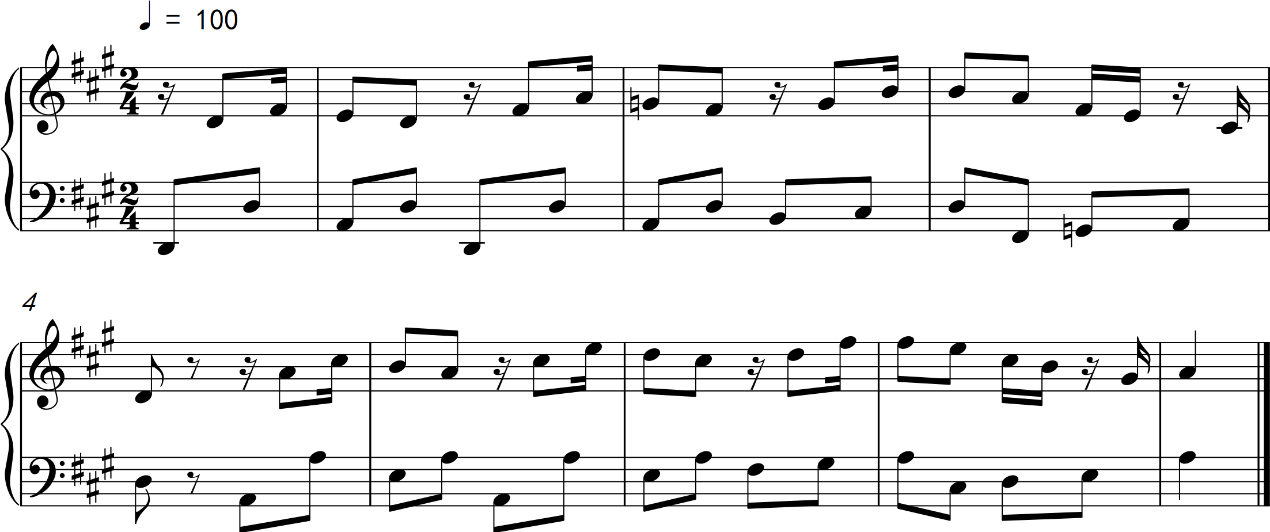
36
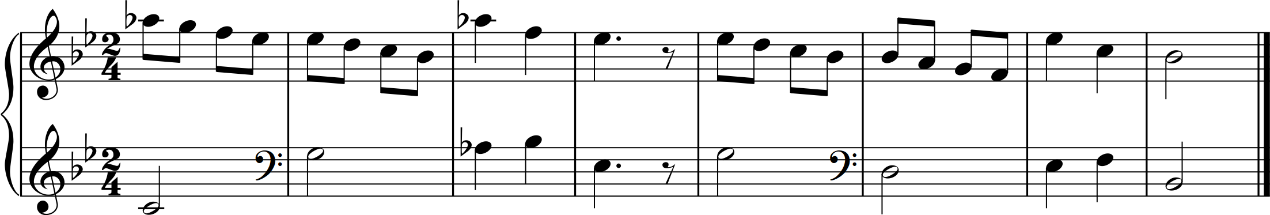
37
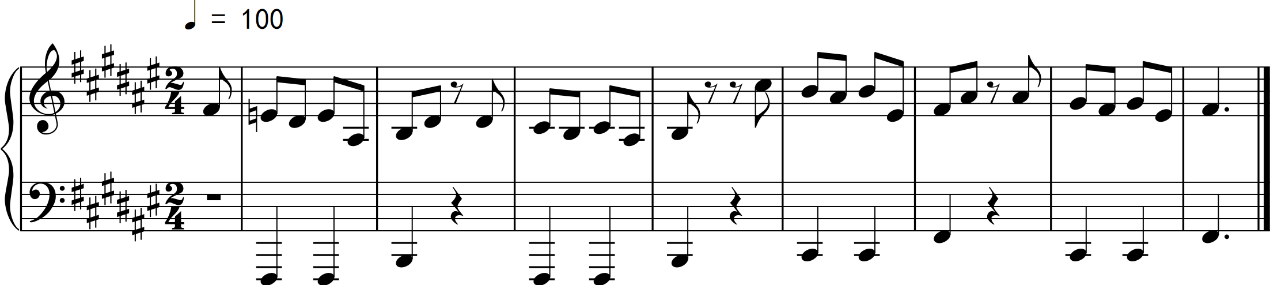
38
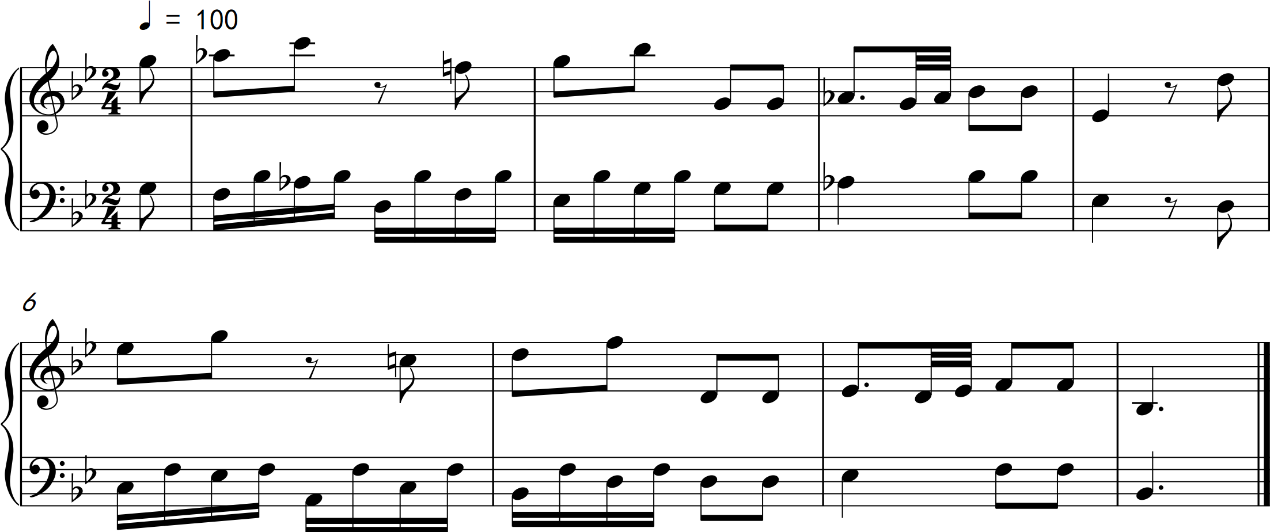
39
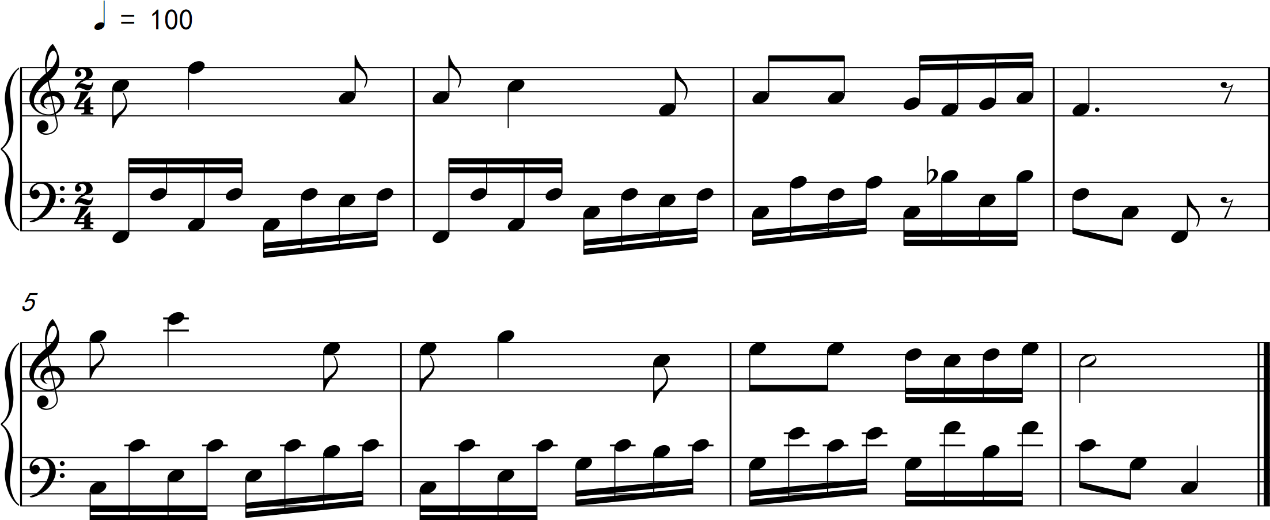
40
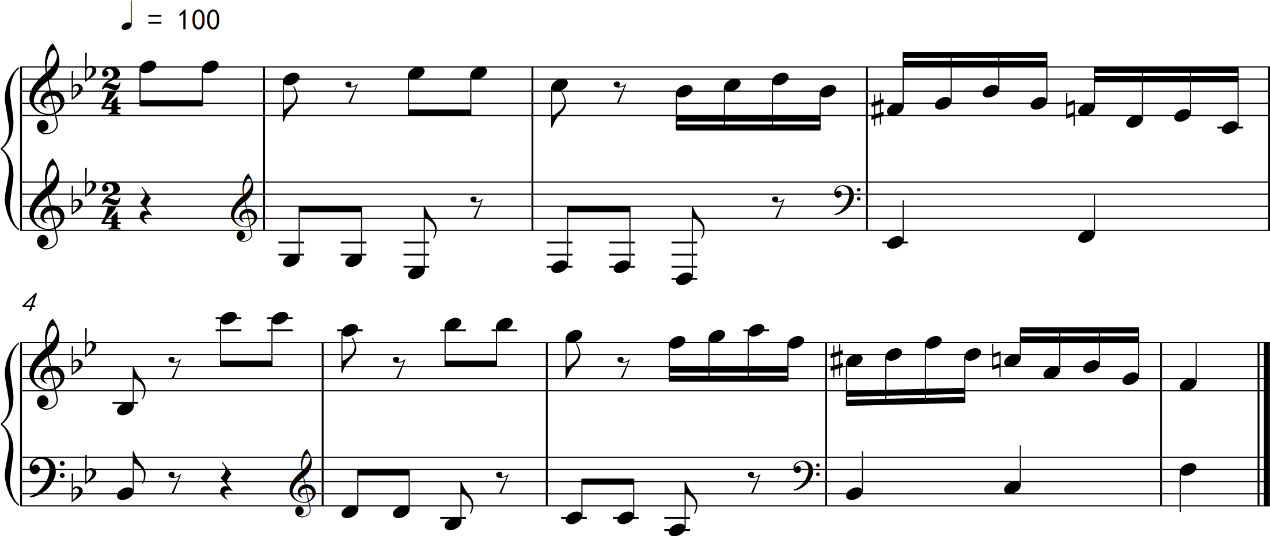
41
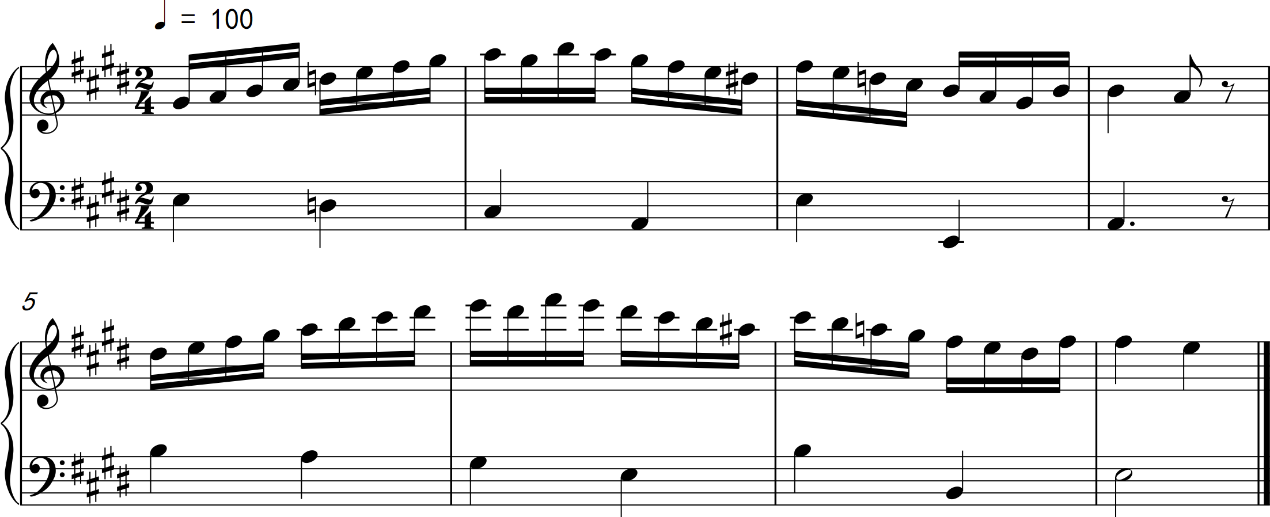
42
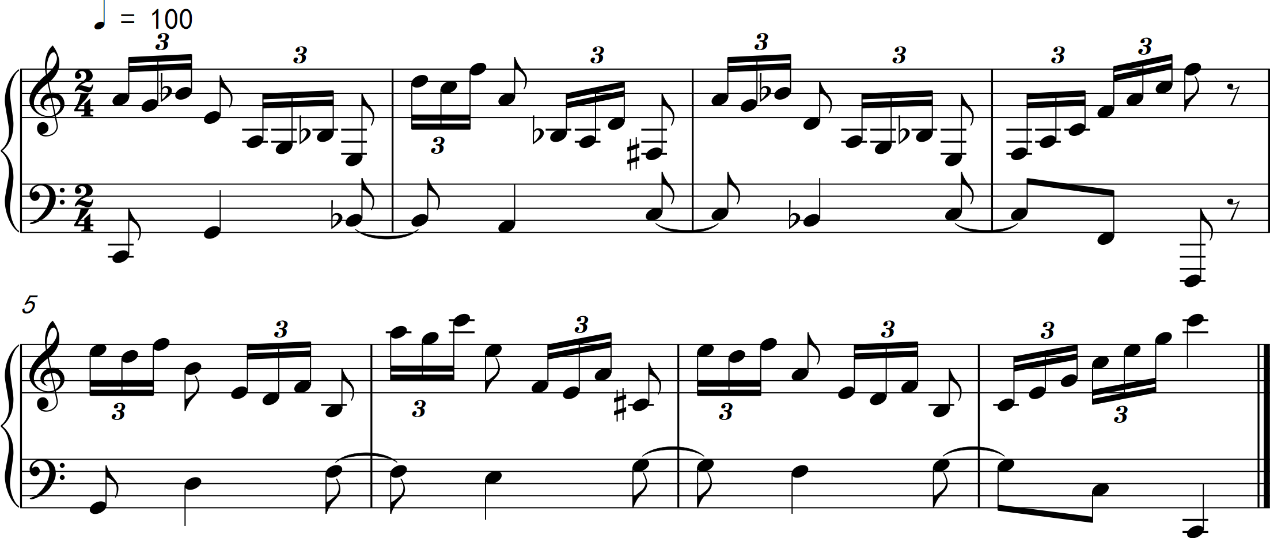
43
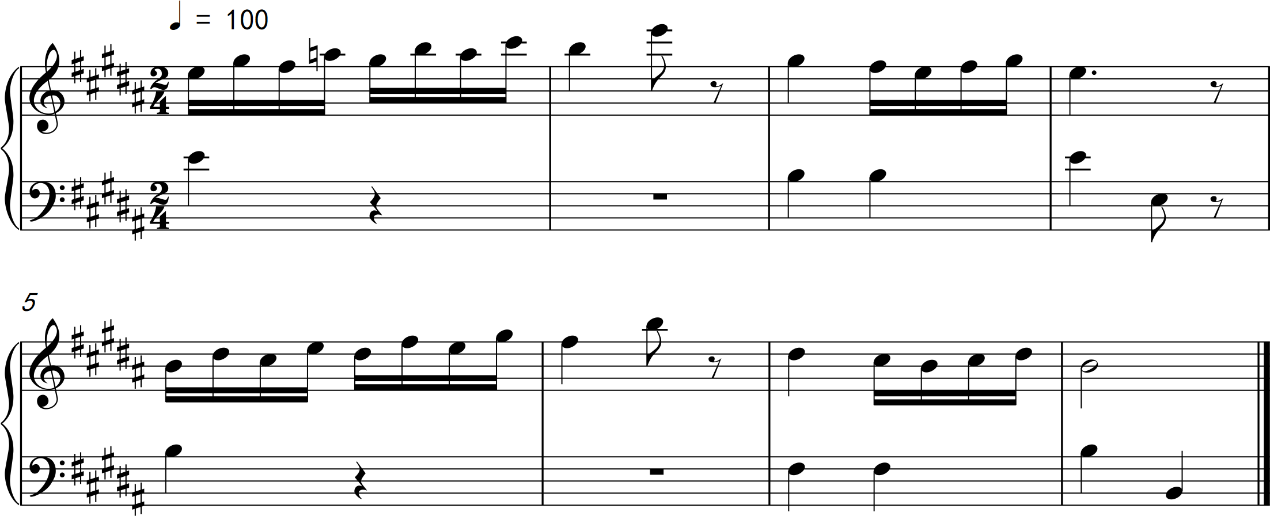
44
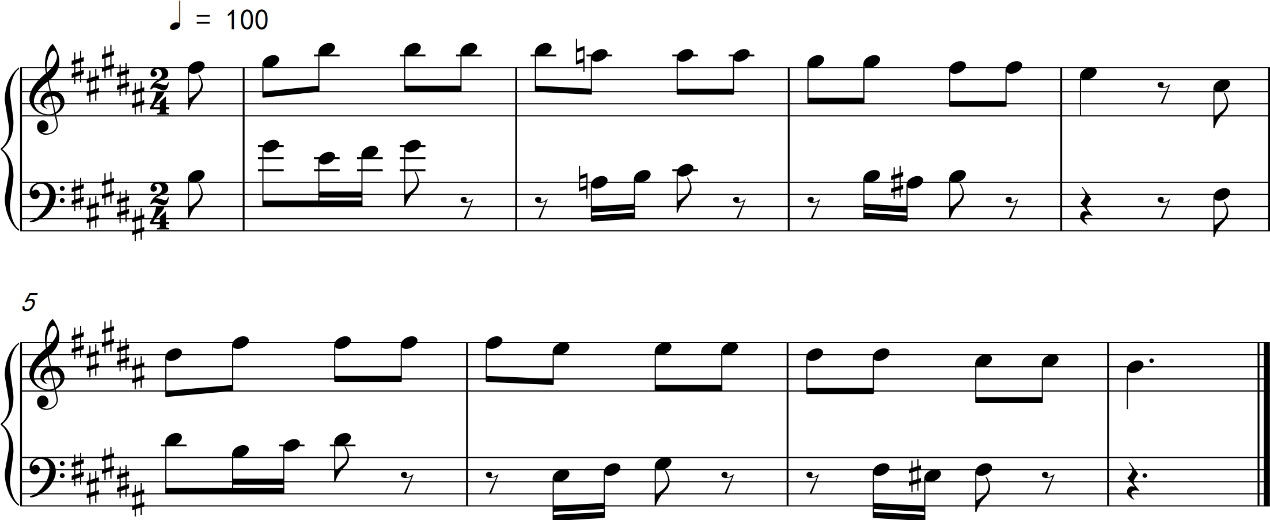
45
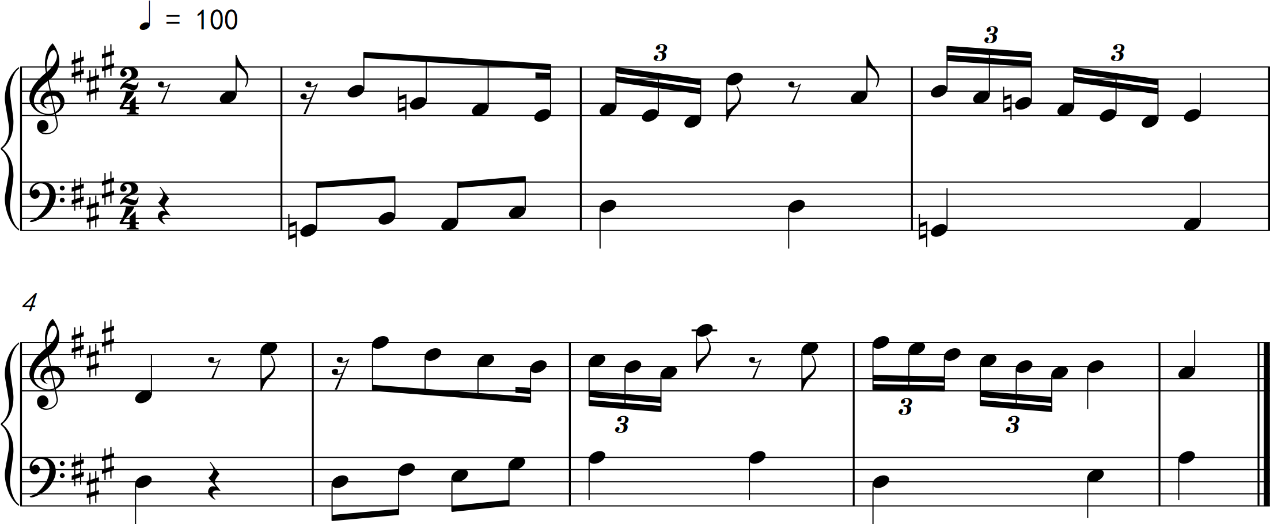
46
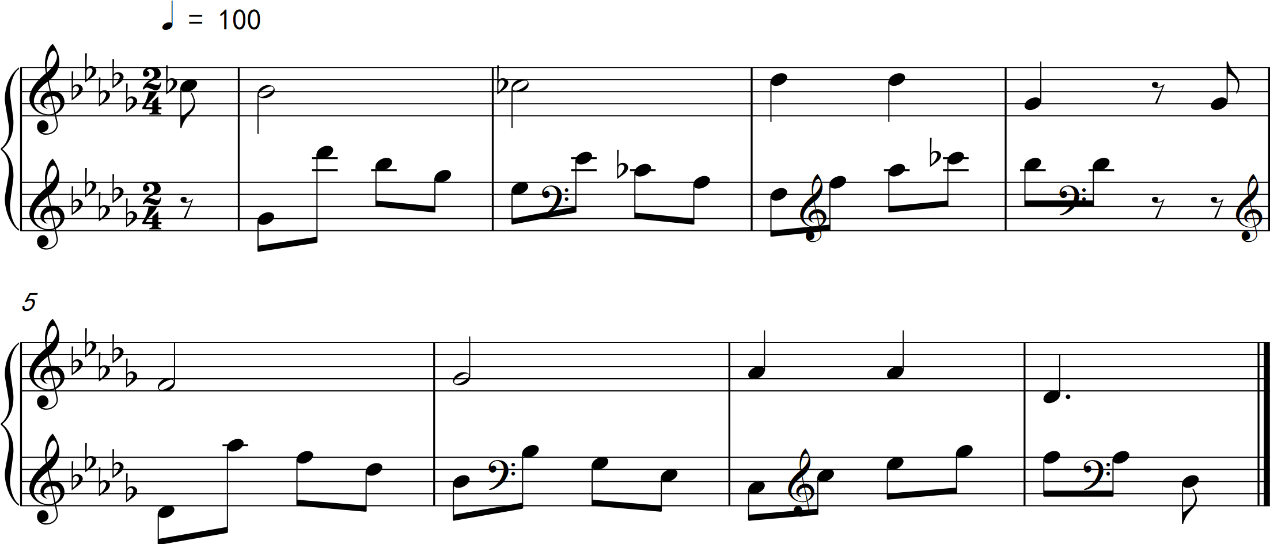
47
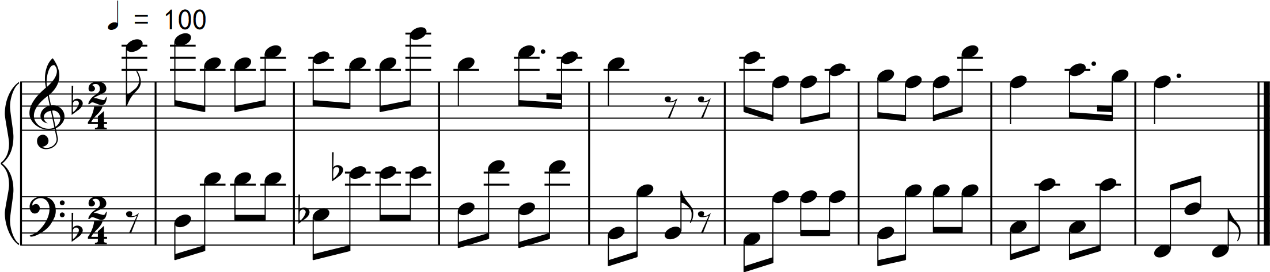
48
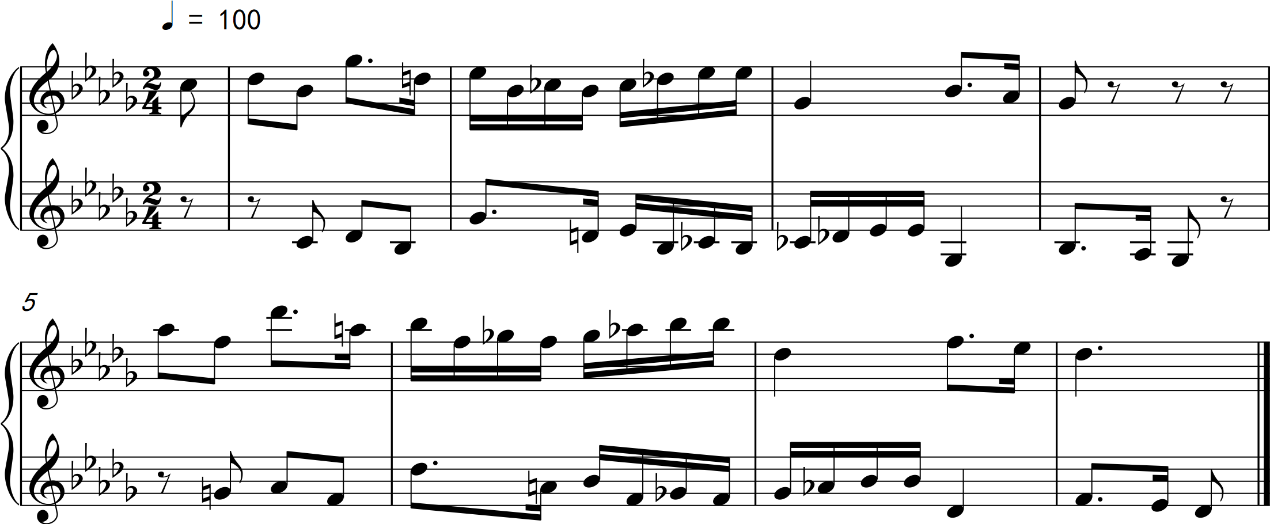
49
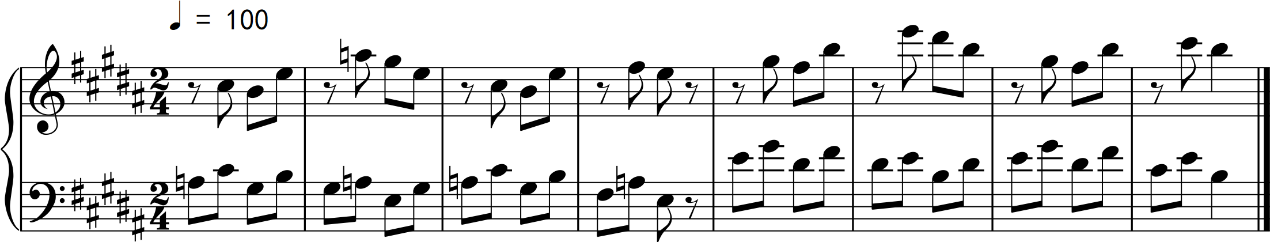
50
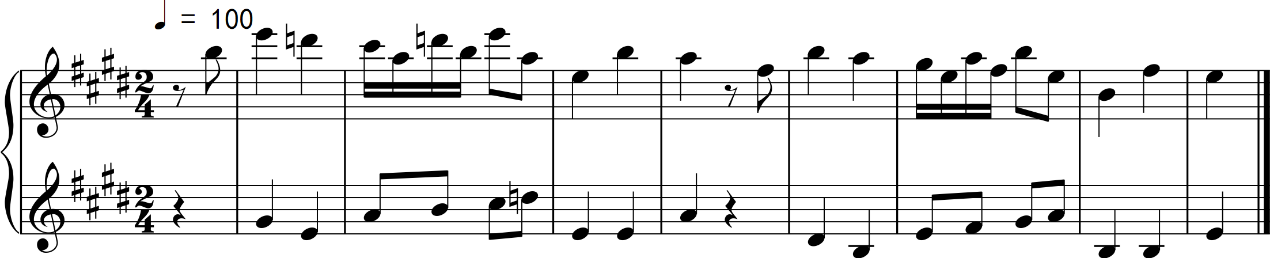
51
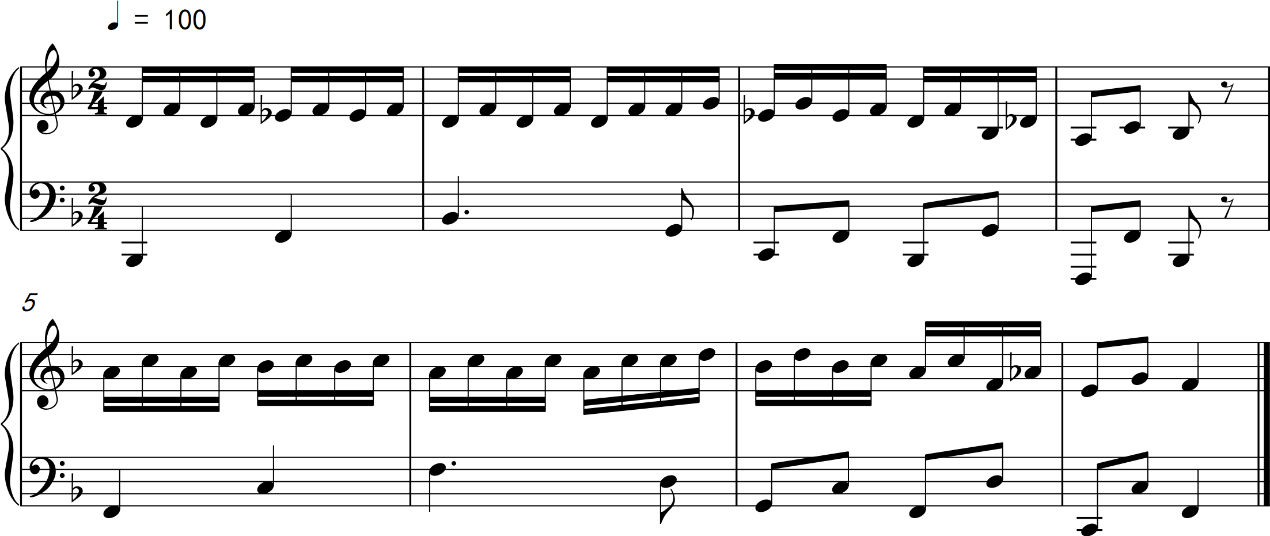
52
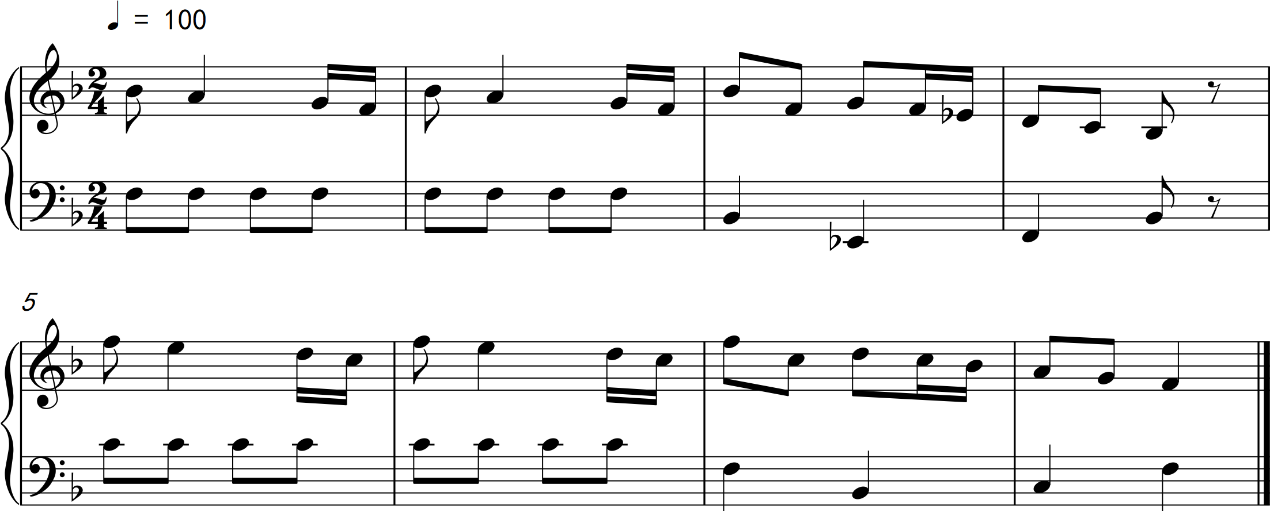
53
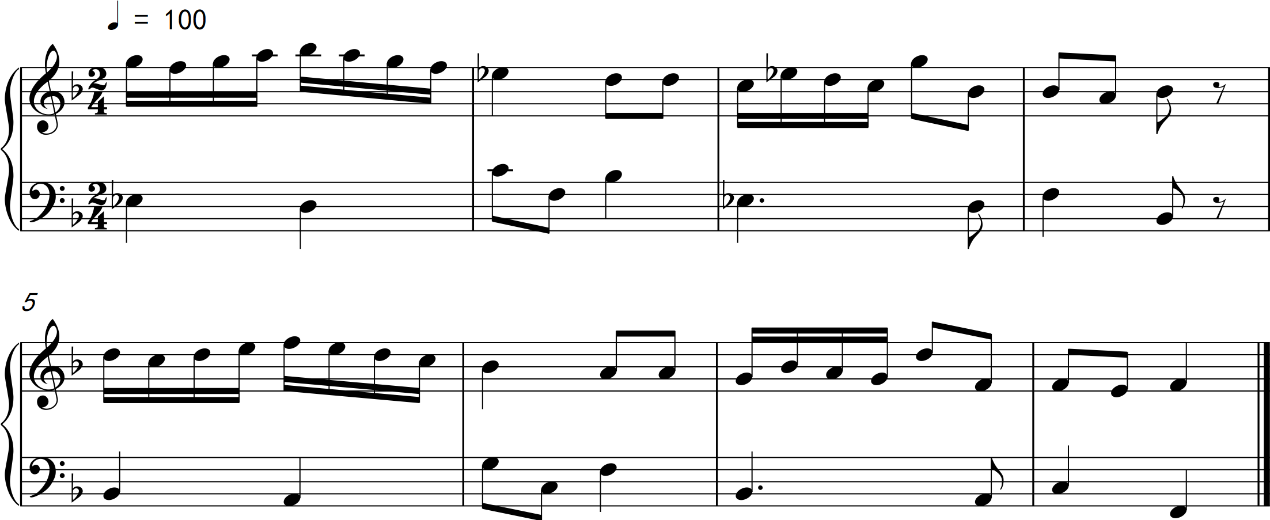
54
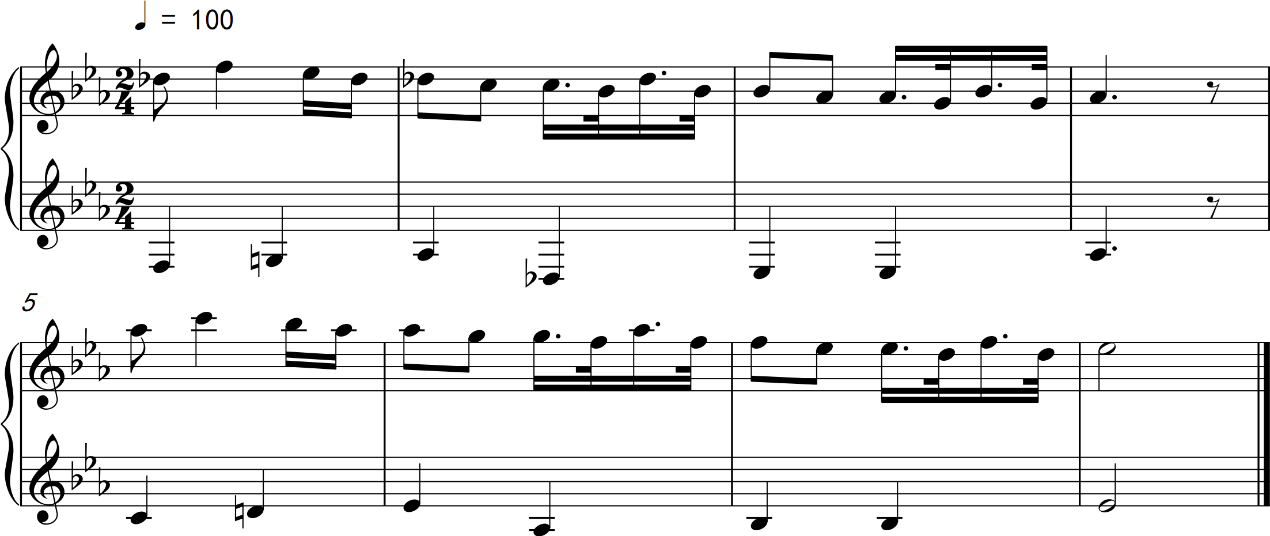
55
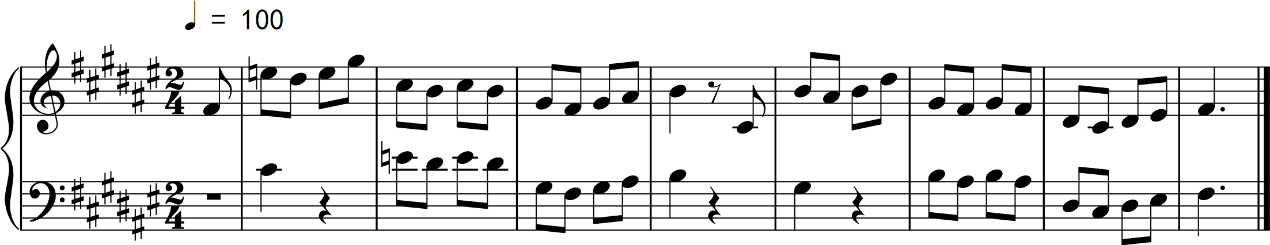
56
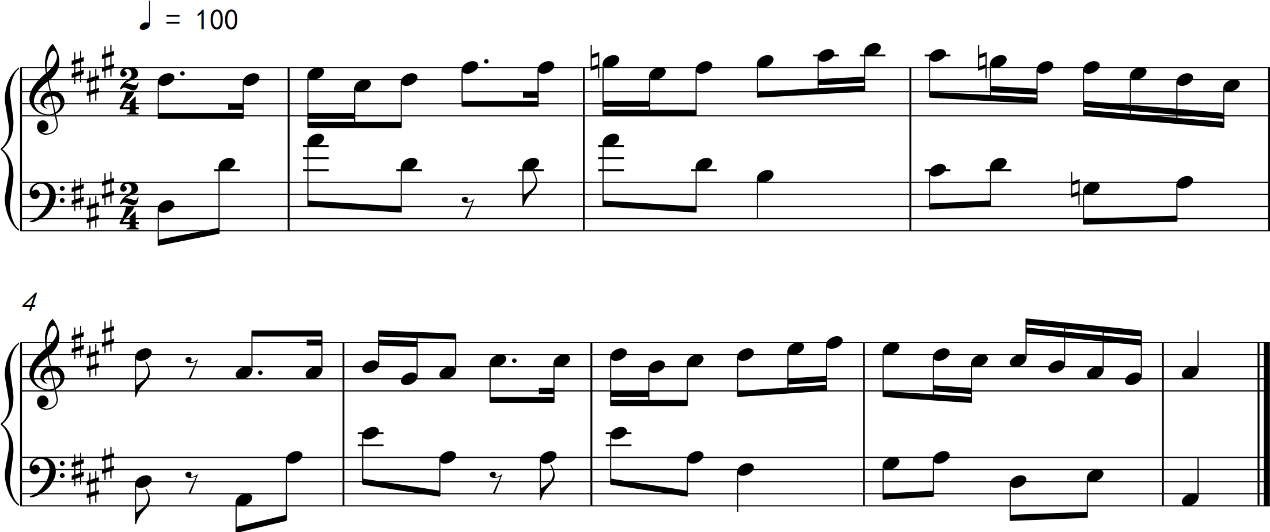
57
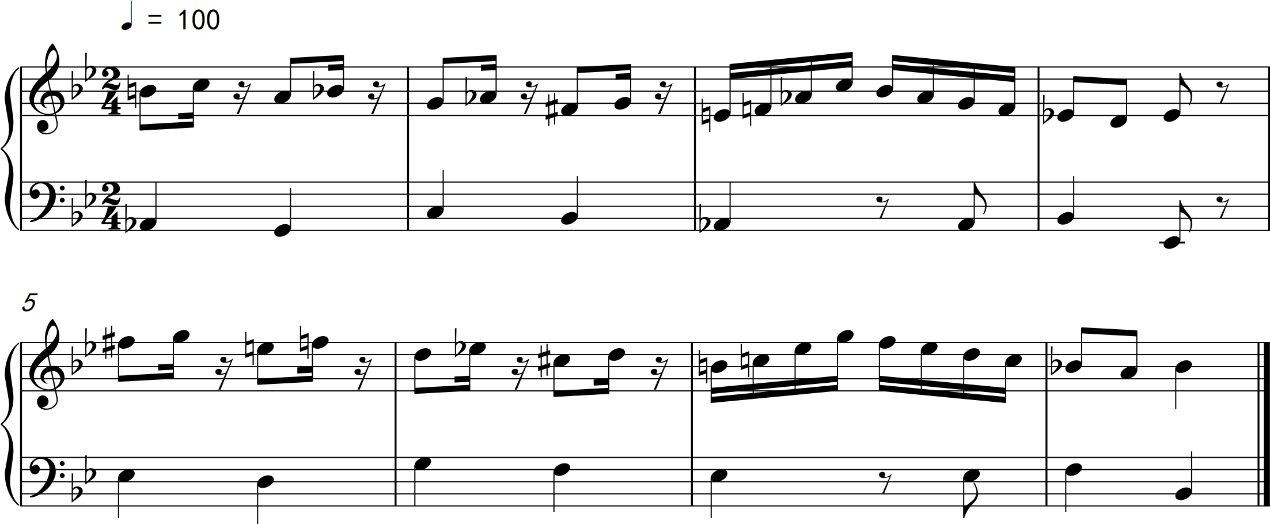
58
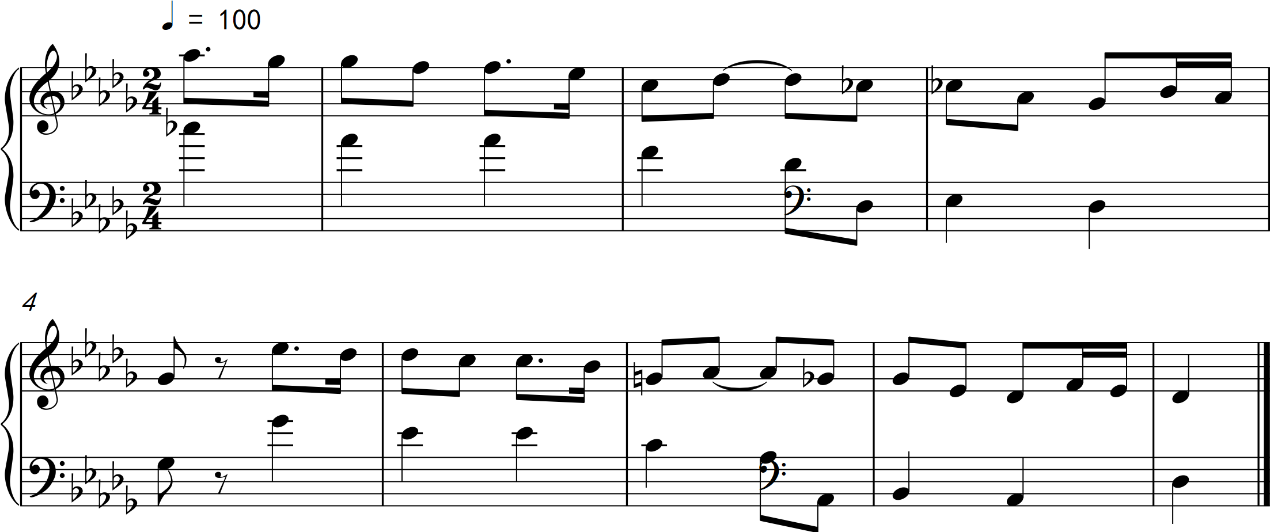
59
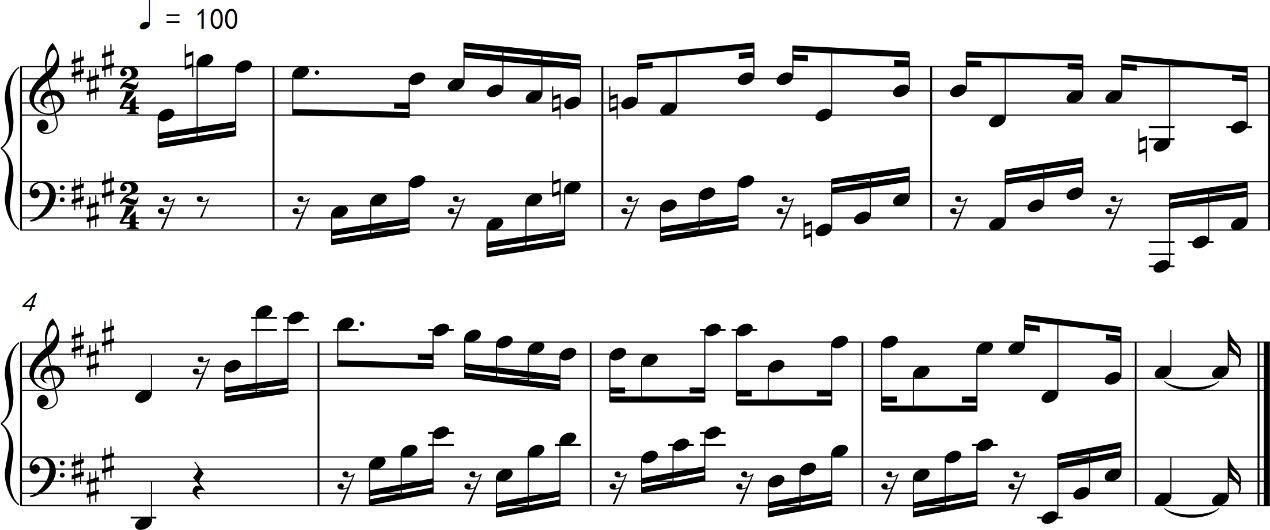
60
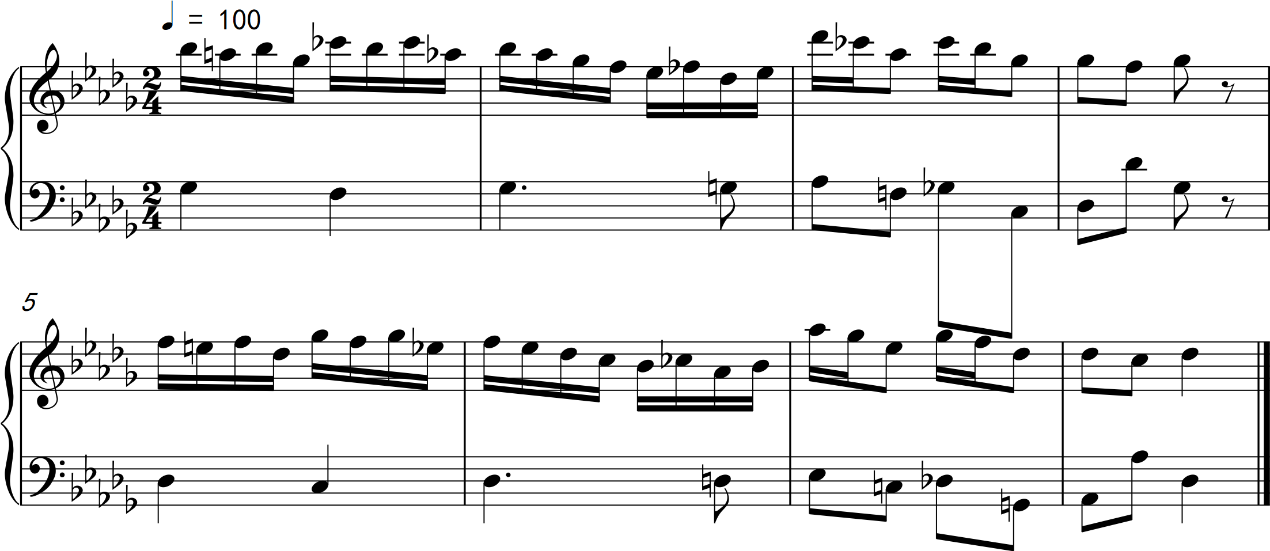

Supplement: Supplementary file 3 — Supplementary Information 3. [file 41598_2022_11949_MOESM3_ESM.docx]
